# Optimising Regionalisation Techniques: Identifying Centres of Endemism in the Extraordinarily Endemic-Rich Cape Floristic Region

**DOI:** 10.1371/journal.pone.0132538

**Published:** 2015-07-06

**Authors:** Peter L. Bradshaw, Jonathan F. Colville, H. Peter Linder

**Affiliations:** 1 Park Planning and Development Unit, South African National Parks, Port Elizabeth, South Africa; 2 Department of Botany, Nelson Mandela Metropolitan University, Port Elizabeth, South Africa; 3 Kirstenbosch Research Centre, South African National Biodiversity Institute, Private Bag X7, Claremont, Cape Town, South Africa; 4 Statistics in Ecology, Environment and Conservation, Department of Statistical Sciences, University of Cape Town, Rondebosch, South Africa; 5 Institute of Systematic Botany, University of Zurich, Zollikerstrasse 107, Zurich, Switzerland; Institute of Botany, CHINA

## Abstract

We used a very large dataset (>40% of all species) from the endemic-rich Cape Floristic Region (CFR) to explore the impact of different weighting techniques, coefficients to calculate similarity among the cells, and clustering approaches on biogeographical regionalisation. The results were used to revise the biogeographical subdivision of the CFR. We show that weighted data (down-weighting widespread species), similarity calculated using Kulczinsky’s second measure, and clustering using UPGMA resulted in the optimal classification. This maximized the number of endemic species, the number of centres recognized, and operational geographic units assigned to centres of endemism (CoEs). We developed a dendrogram branch order cut-off (BOC) method to locate the optimal cut-off points on the dendrogram to define candidate clusters. Kulczinsky’s second measure dendrograms were combined using consensus, identifying areas of conflict which could be due to biotic element overlap or transitional areas. Post-clustering GIS manipulation substantially enhanced the endemic composition and geographic size of candidate CoEs. Although there was broad spatial congruence with previous phytogeographic studies, our techniques allowed for the recovery of additional phytogeographic detail not previously described for the CFR.

## Introduction

### Centres of Endemism as building blocks of Biogeographic Regions

Regionalisation is a fundamental starting point in many fields of biogeography [1, 2]. Simplifying numerous and often complex species distribution data into biogeographically meaningful regions allows for spatial representation [3–5], historical and ecological interpretation [1, 6–9] and conservation planning [10, 11]. The two most common regionalisation categories are Biogeographic Regions (BR)—sometimes referred to as "choria" [4]—and Centres of Endemism (CoEs) [12, 13]. A third, less commonly used, category is Areas of Endemism (AoE) [8, 14, 15].

BRs are areas generally defined by similarity of biotic composition, and diverse classifications have been prepared at global [2, 3, 5, 16], continental [4, 8, 17–25], as well as regional scales [9, 26–28]. These are spatially “complete” in that all operational geographic units (OGUs) *sensu* Crovello [29] or cells *sensu* Crisp *et al*. [8] are assigned to a BR. BRs are also based on near-complete taxon assemblages or species combinations, incorporating both widespread and range-restricted taxa, which usually contribute equally to pattern retrieval.

By contrast, CoEs are defined as areas which are not only rich in (strictly) endemic species, but where endemic species are mostly common to the whole centre [13], thus “delimiting” the centre in question. Typically, CoE studies (e.g. *Sciobus* weevils [12, 30] and African Restionaceae [13]) are clade specific and constitute geographic units defined solely by endemic species, with at least two taxa being endemic [31]. An advantage of defining biogeographic areas using endemic taxa is that local endemic taxa are more likely to be indicative of local contemporary and historical conditions and processes, as opposed to widespread, easily dispersed or adaptive taxa. Perhaps intuitively, there is a presumption that CoEs should be nested within BRs, despite differences in optimality criteria (BR = taxon similarity; CoE = maximising endemism), but in practice this nestedness is not tested. As endemic taxa might be more indicative of local contemporary or historical environmental conditions, we advocate that CoEs should be identified first, followed by the assignment of the remaining OGUs to these CoE areas to form BRs. This approach would ensure that CoEs form the core areas of biogeographic regionalisation analysis, and lessen the likelihood of potential conflicts in biogeographic boundaries between CoE and BR approaches.

AoEs, by definition, are rich in range-restricted taxa [8, 14, 15] and are conceptualised as foci of these taxa. AoEs are indicated by calculating the sum of the inverse range weights of species in an OGU [8, 19, 32, 33], summing some other metric of relative endemism [8, 14, 34], or by summing the numbers of range-restricted taxa occurring in an area [15]. Whereas AoEs highlight areas with high numbers of range-restricted taxa, they do not necessarily constitute areas with clearly defined boundaries, and taxa do not necessarily have to have congruent distributions, or be strict endemics, in contrast to CoEs [13].

### Old problems echoed in modern techniques

In the past, most biotic regionalisations and delimitations of CoEs were based on intuition and expert opinion using a few well-known taxa [5, 35, 36] or collated lists of targeted species [26, 27, 36–38]. Many of these delimitations were therefore informed by the taxonomic knowledge of the authors. Moreover, these authors did not use precisely defined analytical protocols, precluding replication of their methods. Further, with intuitive techniques, it is difficult to objectively minimise the contribution of widespread species that may have limited or conflicting biogeographic information [39, 40]. Numerical methods and increased computational power now allow for the analysis of larger datasets and the clustering of predefined OGUs into biogeographic regions based on shared species [7, 12, 13, 41–47]. These analytical approaches, however, still employ subjective decisions, in particular the nature of the input OGUs, the choice of coefficient to calculate similarity (or dissimilarity) between OGUs, the choice of clustering algorithm to generate dendrograms, and in delimiting clusters on the dendrograms. Essentially, OGUs should be small enough not to lose critical resolution, but large enough not to have spurious absence data [8, 13]. In reality, however, OGU resolution is usually determined by data availability. The search for the optimal similarity coefficient which accurately reflects differences in species composition continues, with three coefficients advocated recently: Jaccard, Simpson and Kulczinsky’s second measure [2, 13, 48, 49]. A general consensus has emerged concerning the use of Unweighted Pair Group Method with Arithmetic Mean (UPGMA) as the preferred clustering algorithm [2, 48]. Several non-hierarchical methods have also been explored, including ordinations and testing congruent distributions (NDM—eNDeMism—[44, 45]). Aside from the commonly employed subjective phenon-line [50] and the fairly complex and somewhat subjective L-method [2, 51], cluster demarcation on hierarchical agglomerative dendrograms has received little attention, hindering reproducibility of studies. We explore some of these issues in more detail below.

### Reducing the noise of wide-spread taxa—removal and/or weighting

Uninformative “noise” from widespread taxa [39, 40] can be decreased by removing conflicting taxa—i.e. those that do not co-occur. This approach, proposed in earlier studies, is based on the concept that species that contribute most meaningfully to biogeographic regions should have largely congruent distribution ranges [39, 52]. This concept has enjoyed a recent revival [30, 46]. Alternatively, widespread species can be down-weighted [13] as these taxa usually do not contribute much to delimiting CoEs. Similarly, taxa occupying a single OGU do not contribute to clustering, and can be removed from clustering analysis [49]. Established down-weighting methods include "inverse weighting" [8, 13, 19, 32, 33] and "Bell shaped weighting" [13]. Here we introduce Integration Weighting, which in contrast to the above techniques directly adjusts taxon weighting in accordance with taxon distributional range properties. Previous clustering approaches using weighted taxon matrices were restricted to Parsimony Analysis of Endemism (PAE) [13], despite UPGMA approaches outperforming PAE on unweighted data [2, 13].

### Candidate CoE cluster demarcation

There are many approaches employed across a variety of disciplines for identifying groups or clusters from a dendrogram (reviewed by Salvador and Chan [51] and Peng *et al*. [53]). Salvador and Chan [51] divide clustering algorithms into four categories: 1) hierarchical; 2) partitioning; 3) density-based; and 4) grid-based. Recently, partitioning techniques which avoid the use of subjective phenon-lines [50] have been employed in biogeographic analysis. An example of such methods is *K*-means analysis [21, 54, 55], but this method requires an *a priori* specification of the number of *K* clusters to be found by the algorithm [22]. Although the number of *K* clusters can be determined using the L-method [2, 51], or a related method [53, 56, 57], it is not always readily apparent which is the most optimal method or optimal number of clusters. Alternatively, the number of *K* clusters can be derived from independent data such as a predefined number of (*K*) environmental zones [55]. However, Salvador and Chan [51] caution that the *K*-means approach does not scale well for larger datasets, although this may pose less of a problem with recent computational advances [58]. Another limitation is that it does not yield topological relationships of clusters [2]. For these reasons, neither the *K*-means nor the L-method are pursued further here. Instead, we introduce an approach adapted from a simple measure of stream complexity (*sensu* Borchert and Slade [59]; Strahler [60]) to efficiently identify appropriate clusters on dendrograms. We refer to this approach as branch order cut-off (BOC).

### Alternative techniques not employed

Although the application of null models has proved successful, analyses to date have been undertaken on relatively small datasets [30, 46, 61]. A literature survey by Fayle and Manica [62] revealed that matrices analyzed using null models typically have ≤ 100 species and ≤ 100 sites. Although advances in computational power and sparse matrix implementation techniques allow for the handling of large, sparse matrices (e.g. Furrer and Sain [63]), there are sufficient statistical uncertainties (high Type I and II error rates) in current null model and species co-occurrence techniques to preclude their use at this stage on large data matrices [61].

Another potential technique is NDM (eNDeMism) [44, 45], which has been shown to outperform both UPGMA [64] and PAE [65]. However, as NDM allows for overlapping CoEs, it is hardly surprising that NDM retrieves more CoEs and CoE endemic taxa than techniques where CoEs are mutually exclusive. While the identification of overlapping CoEs may be useful for taxon based studies, its implications for broader biogeographic regionalisation have yet to be explored, and the results may prove complex to interpret in large datasets due to the relative lack of reductionism. Kreft and Jetz [2] found Non-Metric Multidimensional Scaling (NMDS) ordination to be of limited value, and a spatial autocorrelation approach [8] did not delimit biogeographic boundaries, thus these techniques are not explored here.

### Testing existing and novel techniques on the endemically-rich Cape flora

The Cape Floristic Region (CFR) has 9 383 species of vascular plants in an area of *ca*. 90 760 km^2^, of which just over 68% are endemic [27]. The flora is taxonomically defined by a small number of speciose, largely endemic clades, which are usually absent or unimportant in other floras [66], e.g. *Aspalathus*, *Phylica*, Restionoideae and Diosmeae, or are largely concentrated in the CFR, e.g. *Erica* and Proteae [27, 66]. The floristic uniqueness of the CFR has long been recognised [67, 68], sometimes as a phytogeographic region [4, 69] and sometimes as a floral kingdom [3, 5]. The CFR has numerous range-restricted taxa, and is characterised by high beta and gamma diversity [70–74], providing an ideal natural laboratory to assess the efficacy of weighting techniques for the retrieval of CoEs. Moreover, the region has a rich history of biogeographic analyses using both intuitive [26, 27, 42, 68, 75] and more quantitative approaches [13, 42, 76, 77] which can be used as benchmark comparisons with newly developed techniques. The current biogeographic subdivisions used for the CFR [27] are spatially coarse, thus potentially missing much of the rich phytogeographic detail and concentrations of endemics in the Cape flora.

### Aims

Our primary aim is to improve the quantitative methods for the regionalisation of biotas, by optimising various intermediate steps in the analytical approach. Firstly, we assess preferential weighting systems of range-restricted taxa (Unweighted, Bell, Inverse, and Integration weighting), and test whether weighting improves UPGMA performance for CoE retrieval, and if so, which weighting approach is optimal. Secondly, we assess three similarity coefficients (Jaccard, Simpson (≡ β_sim_) and Kulczinsky2), and two clustering algorithms (UPGMA and PAE). Thirdly, we develop a more objective alternative to the phenon-line commonly used to demarcate clusters by employing the novel BOC technique, designed to delimit each cluster on its intrinsic topological attributes. Finally, we investigate the potential benefits of post clustering GIS manipulation as a means of expanding CoEs to increase their size and endemism, ultimately bridging the gap between CoEs and BRs. Robustness of biogeographic pattern is determined by consensus of the three most optimal clustering techniques. We used a plant distribution dataset from the CFR to test the optimality of our recommendations.

## Methods

### Analysis workflow

**Fig 1:**
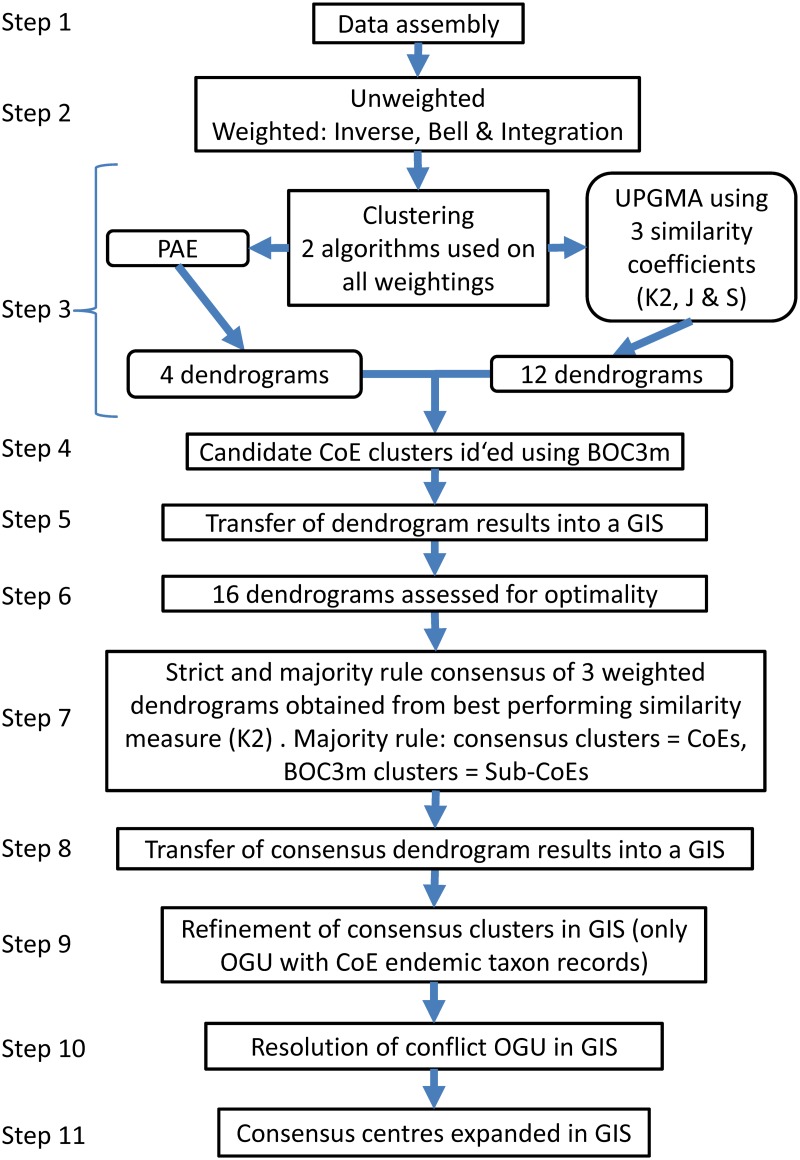
Flow Chart of methods. This flow chart should be read in conjunction with the methods section. [K2 = Kulczinsky2; J = Jaccard; S = Simpson; Clusters delimited using modified branch order cut-off (BOC3m; step 4 and see methods)].

We developed an analysis flow chart that leads through the combinations of four weighting types, three similarity coefficients and two clustering techniques (Fig 1). The consensus (strict and majority rule) of the three best similarity-clustering techniques on weighted datasets was used to derive the final phytogeographic map and to indicate robustness of patterns.

### Database assembly (Fig 1, Step 1)

A dataset comprising a representative floristic sample of the flora of the CFR was assembled. Most of the major clades in the Cape flora (*sensu* Linder [66]) were represented, as well as different growth forms and large numbers of range-restricted species. A detailed taxonomic / functional breakdown of the dataset is provided in Table 1. All species and infraspecific taxa (henceforth “taxa”) were scored as present to Quarter Degree Squares (QDS = *ca*. 640 km^2^ [78]), a longitude-latitude based cell or OGU designation system, used in this study. Although diverse habitats may be included within a single QDS, this scale is commonly used for regional level analysis [79]. Furthermore, due to the long use of the QDS (hereafter cells, *sensu* [8]) by collectors of Cape flora (e.g. Germishuizen and Meyer [80]), this is the finest level at which the data are relatively complete. Collector effort was not formally investigated. Under collecting may result in an overestimation of the levels of endemism, and lead to the recognition of more but smaller CoEs than in a well collected area. It is hoped that the post clustering GIS investigation of CoEs will mitigate some of the potential problems associated with under-collection.

**Table 1:** The size of taxon datasets analysed in the study.

Dataset	Family	Total species in CFR	Taxa analysed	Cape clade	Source^b^
Combined Dataset	All	9 383	4 303	Several	Various, see taxa below in this table
Asteraceae	Asteraceae	1 077	432	Several	Various revisions, BOL
Bruniaceae	Bruniaceae	78	78	Bruniaceae	[42]
Ericaceae	Ericaceae	680	806	*Erica*	[42]
Fabaceae	Fabaceae	764	328	Several	[42]
Geophytes	Numerous	1 635	408	Several	Various revisions
Orchidaceae	Orchidaceae	234	391	Disinae	Herbaria
Poaceae	Poaceae	217	138	Danthoniae	Herbaria
Polygalaceae	Polygalaceae	142	120	*Muraltia*	[42]
Proteaceae	Proteaceae	333	389	Proteae	[42]; Protea Atlas Project (http://www.protea.worldonline.co.za/default.htm)
Red Data List Taxa	Numerous	3 087^a^	1 538	Several	South African RDL [81]
Restionaceae	Restionaceae	342	346	African Restionaceae	Various herbaria; [13]
Rosaceae	Rosaceae	131	121	*Cliffortia*	Revision
Rutaceae	Rutaceae	295	279	Diosmeae	Revisions

Taxon datasets are compared with species diversities from Manning and Goldblatt [27]. The numbers of taxa in the study may exceed those of Manning and Goldblatt [27] for two reasons. Firstly, Manning and Goldblatt [27] list only species, while infra-specific taxa are included here. Secondly, entire clades were analysed where possible, while Manning and Goldblatt [27] list only taxa in their geographically predefined CFR.

^a^ Fynbos biome taxa of conservation concern as reported by Manning and Goldblatt [27]

^b^ Contact details of dataset owners are provided in S1 Table

In order to avoid geographic circularity in determining the boundaries of CoEs along the margins of the CFR, no rigid geographic boundary was enforced to delimit the study area. The initial database covering 1033 cells was refined for weighting and subsequent similarity analysis using two criteria:

1. An adequate representation of range-restricted taxa; therefore all cells containing taxa with ranges ≤ 5 cells were selected.
2. Cells with fewer than 20 widespread taxa (defined here as taxa with range size > 5 cells) were excluded, as under sampled cells may be susceptible to mis-classification [49].

The refined dataset comprised 415 cells and 4 303 taxa, in 383 genera in 64 families. Some 93% of the taxa analysed were present in the CFR, comprising over 40% of the flora of the CFR.

### Weighting (Fig 1, Step 2)

Taxon weighting was employed to reduce the influence of widespread species [13, 40]. Three weighting approaches were compared, and all weighting was scaled from zero to nine (the scale range is arbitrary, but as PAUP can only use a scale of 0–9, other weighting approaches were limited to this scale for comparative purposes). However, as widespread taxa may contribute some useful information, a low weight score of one was assigned to them. An unweighted site by taxon matrix was also analysed for comparative purposes.

For **Inverse weighting (“Inv”)** each taxon was weighted by the inverse of the sum of its full range size, measured by the number of OGUs in which it had been recorded [8, 13, 32, 33]. In order to remove fractions, we multiplied this value by 20 and rounded off to the nearest integer. For example, a taxon in three cells receives an inverse score of 0.33, multiplied by 20, giving 7.

For **Bell shaped curve weighting (“Bell”)** [13] the weight (*y*) of each taxon was determined by the equation *y = e* ^*-axp*^, where *x* is the full species range size in OGUs (QDS cells in this study). The equation has two variables: *a* is an area modifier which affects the horizontal axis for weighting, while *p* affects the steepness of the slope, essentially the vertical axis component of weighting. Linder [13] evaluated the effects of various *a* and *p* values on Cape Restionaceae, and found that *a = -0*.*005* and *p = 3* produce the greatest number of CoEs, but not the greatest numbers of endemic species; whereas using *a = -0*.*005*, and *p = 2* resulted in higher numbers of endemics, but fewer CoEs. As Restionaceae shows similar distributional properties to other Cape clades [42, 66, 82, 83], *a = -0*.*005* and *p = 3* was used here, to optimise the number of CoEs retrieved.

**Fig 2:**
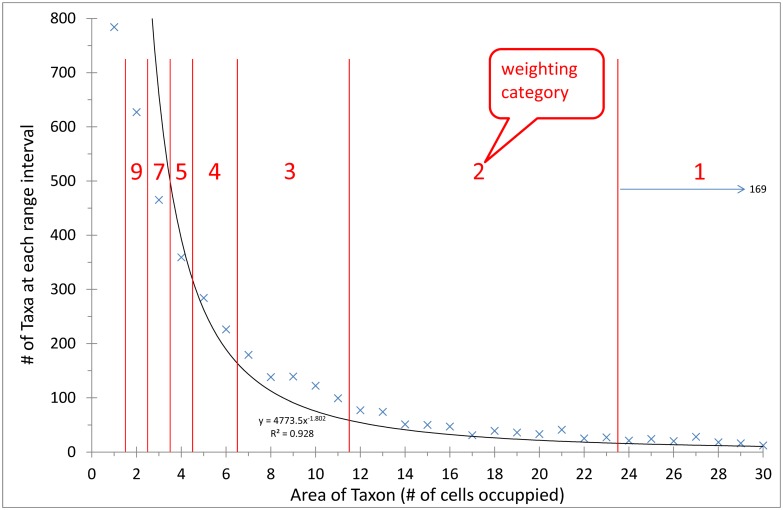
A histogram of the number of taxa in each of the taxon area sizes. The 4 304 taxa analysed were divided into bins dependent on the number of cells they occupied, and the frequency in each bin was calculated. A regression line was then plotted between the bin size and its taxon number. The regression line, graph equation (used for integration), and the goodness of fit value (R^2^) are indicated on the graph.

A novel weighting technique, **Integration weighting (“Int”)**, which exploits the relationship between species range and the frequency of species in these ranges was developed here, and compared to Bell and Inv Weighting. The relationship between species area (range; x-axis) and the frequencies of species of that particular area (y-axis) was used to plot a line of best fit (y = 4773.5x^-1.802^; R^2^ = 0.928), which is specific for the dataset analysed (Fig 2). The area under the curve was then divided into nine equal area portions (the nine weighted area categories), beginning at taxon range size of two cells (the minimum range size contributing to clustering) and ending at the most widespread taxon with a range of 169 cells. The nine equal area portions were given descending weighting along the x-axis (Fig 2). Species with ranges in these nine weighting area categories were weighted accordingly. This weighting technique adapts to individual datasets (whether entire floras, samples of floras, or taxonomic groups) *a priori*, without having to adjust the two variables (*a* and *p*) of Bell weighting for optimisation. Int takes into account the relative distributional sizes of species as a proportion of the entire clade or dataset, as well as the relative frequencies of the species in each distribution category, by integrating the relationship between these two properties.

### Similarity measures (Fig 1, Step 3)

Possibly the most important attribute of a similarity coefficient used for biogeographic analysis is that shared absences must not be taken into account [13, 48, 84–87]. Our analyses were restricted to three (Fig 1) of the more commonly used or advocated measures in biogeographic analysis. Variables of the similarity equations follow the standard format of: *a* = taxa shared between OGUs, *b* = taxa unique to first OGU, *c* = taxa unique to second OGU. Jaccard ($J=\frac{a}{a+b+c}$) is arguably the most frequently used similarity measure in biogeographic studies [13, 48, 77, 88–90] and constitutes a good baseline for comparison. Here it is compared with two coefficients that are less affected by relative differences in richness of the pairwise OGUs being compared—a criticism of Jaccard [49]. β_sim_—a derivative of Simpson [91] ($S=\frac{\text{min}(b,c)}{\text{min}(b,c)+a}$ or alternatively $S=1-\frac{a}{\text{min}(b,c)+a}$; hereafter referred to as “Simpson”)–has been used as a measure of taxon turnover [92, 93]. Recently it has been applied to identify BRs [2, 22]. Kulczinsky’s second measure (hereafter referred as “Kulczinsky2”) ($K2=\frac{\frac{a}{2}(2a+b+c)}{\left(a+b)(a+c\right)}$) [48, 49, 76, 94], has been used less frequently. The three coefficients (Simpson, and the one-complement (dissimilarity = 1 minus similarity) of Jaccard and Kulczinsky2) were used to calculate dissimilarity matrices for the four weighting schemes (Fig 1: Step 3), and these clustered using UPGMA. To simulate weighting, taxa were duplicated in the site by taxon matrix as dictated by their weighting score, and given a unique identifier. The *sim* function in the simba library [95] was used to produced 12 dissimilarity matrices (from the combinations of similarity coefficients and weighting schemes). The degree of correlation between the 12 dissimilarity matrices was assessed with pair-wise Mantel tests, using the *mantel* function in the vegan library [96] with the default 999 permutations and Pearson product moment correlation. The result was plotted as a dissimilarity dendrogram of the degree of correlation between dissimilarity matrices using the *hclust* function in the stats library [97] with the average grouping method (≡ UPGMA). These analyses were conducted in R [97].

### Clustering algorithms (Fig 1, Step 3)

PAE [12, 88, 98] is included for comparison with similarity / UPGMA, as weighted site by taxon matrices have not previously been clustered with UPGMA. Clustering was undertaken in R [97] using the *hclust* function in the stats library [97] with the average grouping method (≡ UPGMA). PAE is used frequently in the search for CoE [7, 9, 12, 13, 43, 99–101], but has been criticised as a historical biogeographic technique [102–104]. Here we use PAE only for pattern retrieval and not for historical interpretation. Further, due to the relatively short dispersal distances in fynbos [105–107] and the regularity of fire as a potential vicariance mechanism [108], the criticism of zero rooting [103] may prove less of a theoretical hindrance in the CFR, where dispersalistic interpretations are less frequently invoked. PAUP4.0b10Win [109] was used to locate the set of most parsimonious trees. Following Linder [13], 500 random addition sequences were performed and the set of shortest trees kept, followed by tree bisection and reconstruction, until completion or an upper limit of 10 000 trees was reached. A strict consensus tree was then calculated.

### Candidate CoE cluster demarcation on dendrograms using the Branch Order Cut-Off (BOC) method (Fig 1, Step 4)

Candidate CoE clusters were identified using a branch order technique, which was applied to all dendrograms. The premise of this technique is nearly identical to Strahler stream order calculations [60], where dendrogram branch orders are assigned similar to Strahler stream orders, with the “cut-offs” for clusters (CoEs) determined by the branch order level. We refer to this approach as Branch Order Cut-off (BOC). Conceptually, a core CoE should act as a nucleus, and “pull in” neighbouring OGUs, until they encounter another expanding “CoE nucleus”. An alternative analogy is that each “branch” or CoE is a distinct “sub-catchment” area. This should intrinsically allow the size of each of the CoEs to vary according to the ranges of the endemic taxa present (taxa in the western CFR generally have narrower ranges than those in the eastern CFR [110]).

**Fig 3:**
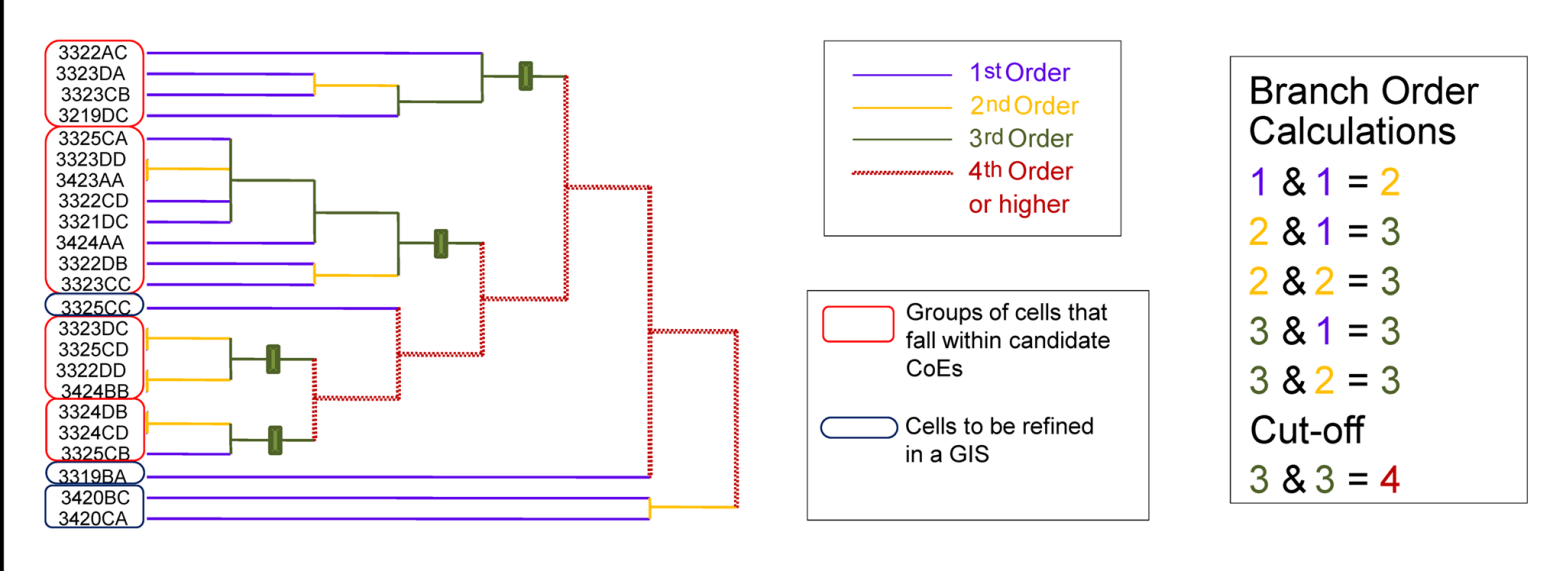
A diagrammatic example of the modified third order branch cut-off (BOC3m). The cut-offs are displayed as bold vertical lines (green), and the resulting candidate CoE clusters are indicated with red blocks around the cells.

For the identification of CoEs in the CFR, standard second order Strahler branches were still too terminal (CoE too small and disjunct), and standard third order Strahler branches were often too basal / deep to allow for meaningful cut-offs (i.e. CoE too large, thus losing phytogeographic detail). Therefore, the technique applied here for CoE identification deviated from a pure Strahler approach in that a third order branch could be formed where either a first or second order branch joined another second order branch, and was thus referred to as modified third order branch cut-off (BOC3m). This change was applied to allow the formation of a more intermediately placed cut-off, optimising the number as well as the size of CoEs. The rest of the branch order assignment was identical to that of Strahler’s. Dendrogram terminals are first order (Fig 3, blue branches), which link together to form second order branches (Fig 3, orange branches); two second orders or a second and a first order join together to form a third order grouping (Fig 3, green branches), with the cut-off being placed on the most basal parts of the third order branches—forming a nested candidate CoE cluster (Fig 3, green block) —before they become a fourth order branch (Fig 3, yellow branches). This approach was applied to all 16 dendrograms (Fig 1: Step 4).

The candidate CoE clusters from these 16 analyses were then plotted as candidate CoEs on maps in a GIS (Fig 1: Step 5). These potential CoE clusters were further refined by firstly removing potential CoE clusters that have no endemic species, and pruning cells from the candidate CoE clusters that did not include any of the endemic species of the CoE. Single cell candidate CoEs (with single cell endemics) were also removed, as these candidate CoEs were not necessarily retrieved by the clustering technique. The performance of the 16 analyses was then assessed.

### Assessment of the performance of different techniques (Fig 1, Step 6)

There are no universally accepted criteria for assessing the performance of different regionalisation techniques. The assessment criteria not only influence the selection of regionalisation technique, but may also influence the setting of optimality parameters, especially whether to maximise numbers of endemics or of CoEs [13], which may be conflicting objectives. For the assessment of our regionalisation techniques, we considered the most important optimality criteria to be: 1) numbers of endemic taxa; 2) numbers of CoEs; 3) the numbers of cells assigned to CoEs; and 4) CoE taxon richness. Area, endemism and richness of CoEs should in theory all be positively correlated with each other [6, 111, 112], and all three negatively correlated to the number of CoEs [13]. For example, in a finite area, if more CoEs are delimited, then the average area of the CoEs is smaller, and consequently there are fewer endemic species in each CoE. The 16 regionalisation techniques were ranked by the sum of the rankings for the criteria we used for assessment (Fig 1: Step 6).

### Establishing a support hierarchy for CoE delimitation (Fig 1, Step 7)

In order to determine the robustness of CoE patterns, a strict consensus and majority rule consensus were computed from the three dendrograms from the Kulczinsky2 similarity analysis, and the results depicted on a map. We treated the smaller clusters delimited from the majority rule consensus tree using BOC3m as consensus Sub-CoEs (*sensu* Weimarck [26]), whereas the larger nearly basal clusters were designated as consensus CoEs (see S1 Fig). This established two floristic unit levels, with a hierarchical level below CoE (*sensu* Weimarck [26]). These results where then transferred to a GIS [113] (Fig 1, Step 8), and refined manually by the removal of cells without endemic taxa (Fig 1, Step 9).

### Manual GIS expansion of CoEs

After refining the consensus floristic units (CoEs & Sub-CoEs) in GIS, by firstly removing potential floristic unit clusters that have no endemic species and pruning cells from the candidate floristic unit clusters that did not include any of the endemic species of the floristic units. The GIS was then used to manually assign remaining cells to CoEs / Sub-CoEs using maximisation of endemism in floristic units as the optimisation criterion. This firstly focused on investigating whether single cell candidate floristic units (with single cell endemics) could be combined with larger neighbouring floristic units of the same level to increase relative levels of endemism, albeit at the cost of the numbers of floristic units. Similarly, cells that were “unplaced” in CoEs due to conflict in the majority consensus analysis between the different weighting techniques employed (Fig 1, Step 10) were investigated for placement in CoEs. The investigation was then expanded to include cells still unassigned to floristic units, but which could potentially add further endemic taxa to the floristic units (Fig 1, Step 11). In ambiguous cases, where a cell could be assigned to more than one floristic unit, the net increase in endemicity was used; if this was equal, then the “new” endemic species with the smallest distribution of the conflicting species was favoured. If this was also indecisive, the relative increase in floristic unit endemicity (i.e. the percentage increase in number of endemics in a floristic unit) was also considered. Finally, taxa entirely outside of the floristic units identified by clustering were assigned to newly created floristic units in the GIS.

The final step to bridge the gap between CoEs and BRs was the assignment of remaining CFR cells (that did not contain endemic taxa) to CoEs (Fig 1, Step 12). This was restricted to cells falling entirely within the CFR, as defined by the CoEs identified by this study. To assess the value of the additional post clustering analysis in GIS, increases in floristic unit optimality criteria (as described above) were reported. The mechanism used to assign cells to floristic units was mapped.

### Upper hierarchy of core CFR CoEs

To establish the relationships between our core CFR CoEs, and whether they clustered within the traditional CFR BRs of Weimarck [26] and Manning and Goldblatt [27], further cluster analyses were performed using the CoEs derived above as OGUs. Clustering was repeated, as deeper (more basal) level hierarchical structures of dendrograms may be more susceptible to distortion than more terminal branches [48]. Taxa received the same weightings in the cell level analysis (Bell, Inv and Int), and both Kulczinsky2 and Jaccard similarity measures were employed for comparative purposes. The consensus of the three weightings for each similarity measure was calculated. Standard second branch order cut-off levels (BOC2) were used to demarcate the boundaries between higher order BRs, as these were deemed most appropriate. The BRs of the Kulczinsky2 CoE clustering were mapped.

## Results

### Effects of weighting on the dataset matrix

**Fig 4:**
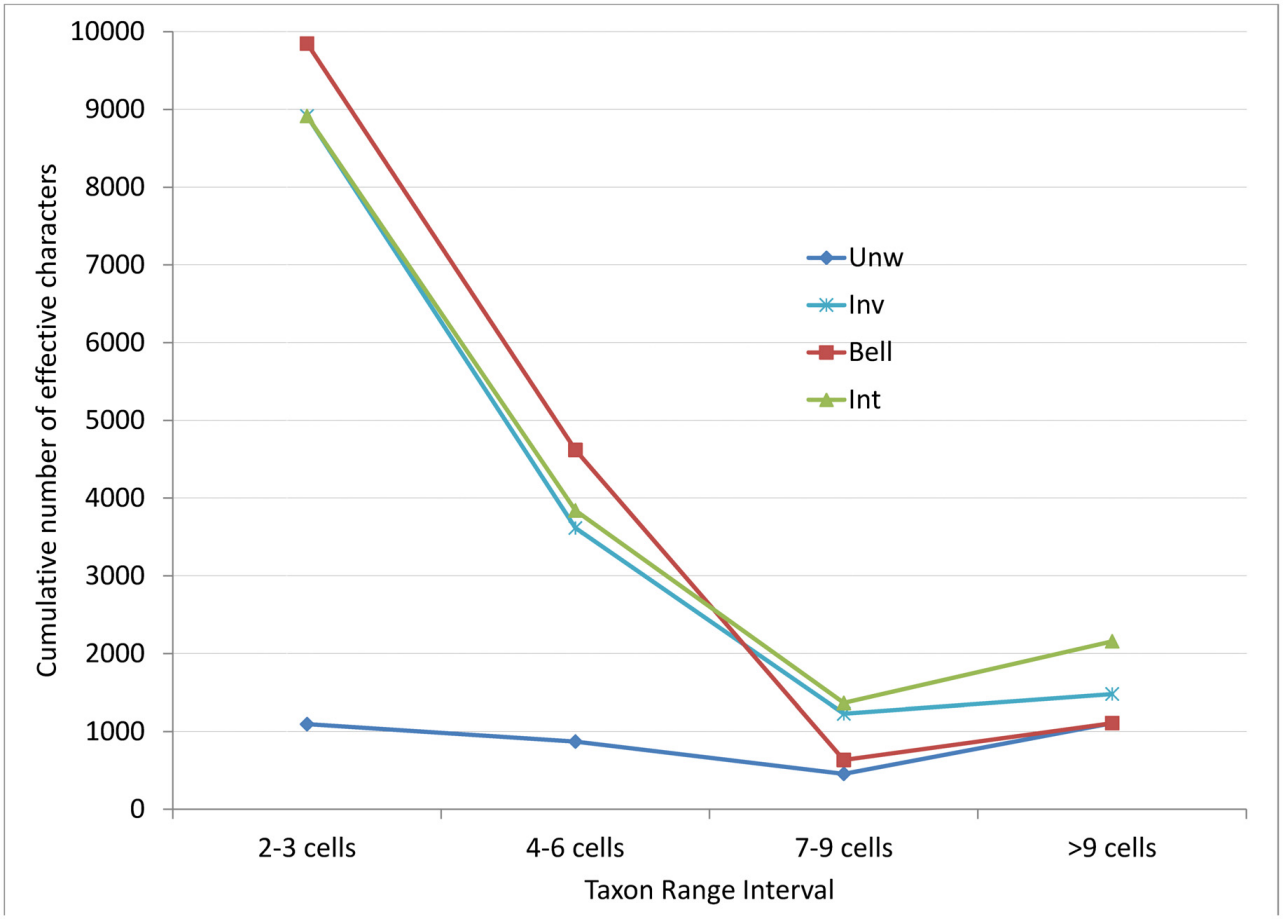
The bin frequency distributions of weighted matrix characters generated by the different weighting techniques. Taxa restricted to a single cell were excluded as they are not effective for clustering. Total site taxon matrix character numbers are reported in Table 2.

The three weighting methods resulted in similar overall numbers of matrix characters (15 987 to 17 028), compared to the much fewer 4 478 for the unweighted analysis (Table 2) for the site by taxa (character) matrices. Bell weighting displayed the sharpest relative decrease in weighting associated with increasing taxon range size, followed by Inv and Int weighting (Fig 4); however, the latter two were very similar, although Inv was arguably preferable with fewer taxa in the final lowest weighted category (> 9 cells, Fig 4). Consistent with the above, Bell generated the highest numbers of effective characters in the smallest species range category, followed closely by Inv (Fig 4).

**Table 2:** The optimality criteria of the 16 analytical (weighting and clustering) and consensus approaches employed.

Weighting	Unweighted				Inverse				Bell				Integration				Consensus CoE	Consensus Sub-CoE
Coefficient	J	K2	S	PAE	J	K2	S	PAE	J	K2	S	PAE	J	K2	S	PAE		
# Characters*	4,300	4,300	4,300	4,300	15,987	15,987	15,987	15,987	16,954	16,954	16,954	16,954	17,029	17,029	17,029	17,029		
**# CoE endemics**	463	472	299	448	490	491	395	483	496	522	404	472	466	476	418	463	1,551	1,141
**# CoE taxa**	4,144	4,169	3,909	4,112	4,199	4,218	4,156	4,193	4,212	4,244	4,200	4,179	4,187	4,217	4,173	4,177	4,347	4,089
**# CoEs**	47	57	53	38	57	67	63	57	63	66	67	59	59	68	63	60	66	57
**# of cells in CoEs**	177	197	156	162	208	241	229	203	223	256	251	201	201	237	237	199	397	112

“# Characters” = number of characters generated by a weighting technique for the site by taxon matrices; # CoE endemics = numbers of taxa endemic to the CoEs; # CoE taxa = the total number of taxa represented in the CoEs; # CoEs = the total number of CoEs retrieved; # of cells in CoEs = the total number of cells assigned to CoEs, i.e. CoE area) for the individual weighting techniques and similarity measures. J = Jaccard, K2 = Kulczinsky2, S = Simpson.

### Comparison of clustering algorithms, weighting and similarity measures

**Fig 5:**
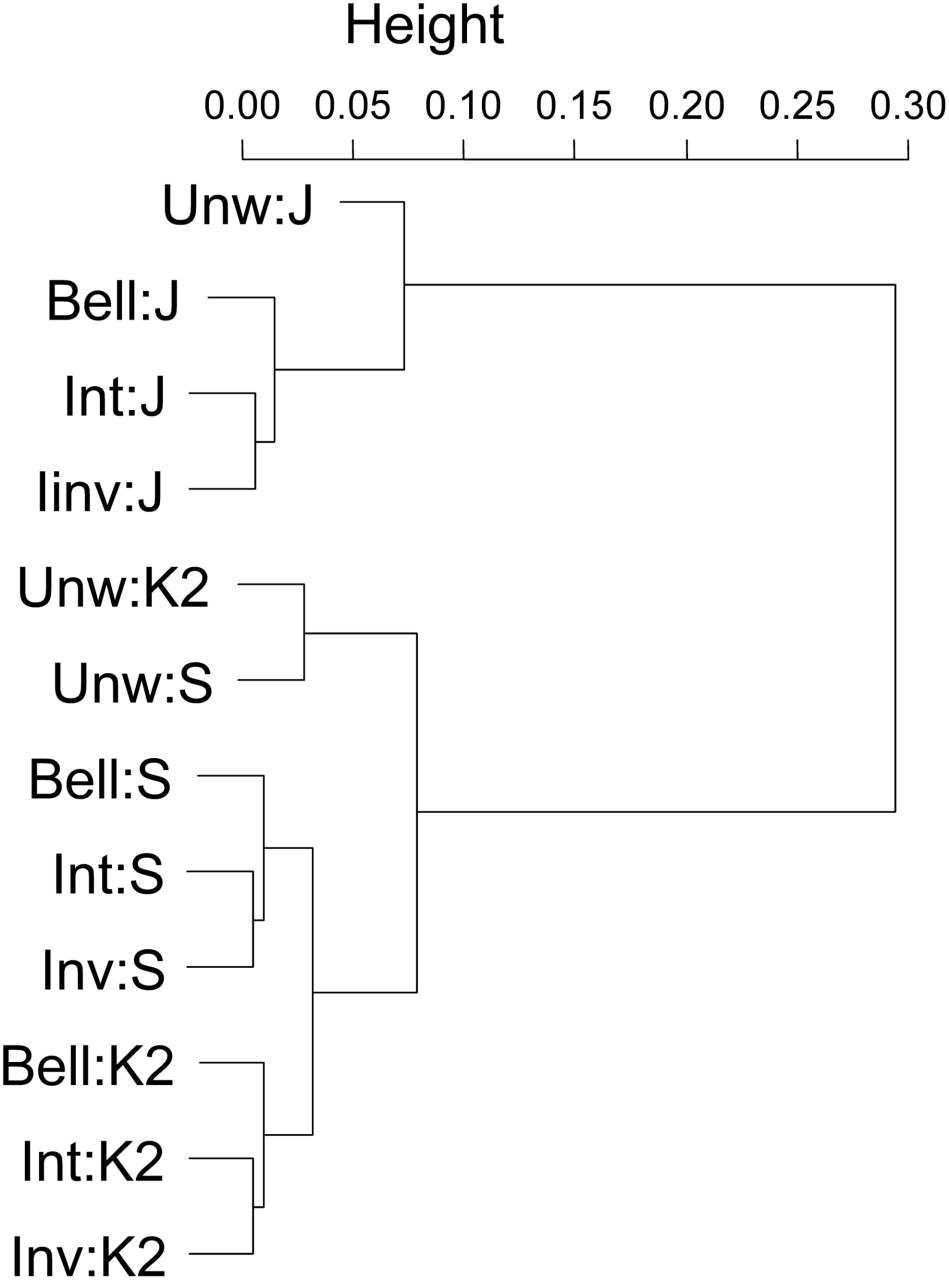
A dendrogram of the correlation between the 12 weighting-dissimilarity matrices. Mantel Tests were undertaken using Pearson correlation and 999 permutations. The actual correlation values are provided in S2 Table. All dissimilarity matrices were significantly correlated with p < 0.001. [Unw = unweighted, Bell = Bell weighting, Int = Integration weighting, Inv = Inverse weighting, K2 = Kulczinsky2; J = Jaccard; S = Simpson].

The performance criteria of the different analysis permutations (different weightings, similarity coefficients and clustering algorithms) in delimiting CoEs are summarised in Table 2. Of the top five ranked analytical approaches (all UPGMA, see Tables 2 & 3), the top three utilised the Kulczinsky2 similarity coefficient (Bell:K2 performed best, followed by Inv:K2 and Int:K2); and three techniques (1^st^, 4^th^ & 5th) employed Bell weighting function (Table 3). A summary of the optimality of the weighting techniques employed and the clustering / similarity coefficients (Table 4) indicated that overall Kulczinsky2 performed better than Jaccard, while Simpson performed the least optimally. Pair-wise Mantel Tests of the correlation between the dissimilarity matrices indicated that all dissimilarity matrices were significantly correlated (p = 0.001 for all comparisons; R^2^ values in S2 Table). Overall, dissimilarity matrices generated using the same coefficient but different weighting approaches were more similar to each other than dissimilarity matrices based on different coefficients but the same weighting approach (Fig 5). Further, the Kulczinsky2 and Simpson dissimilarity matrices are more similar to each other than to Jaccard dissimilarity matrices (Fig 5). Of the weighting techniques, Bell performed most optimally, followed by Inv, then Int weighting (Table 4), although these absolute rankings masked similar results (Tables 2 & 3). PAE ranked seventh, ninth or twelfth depending on the weighting, while the unweighted data performed poorly in almost all CoE performance measures (Tables 2 & 3). Weighted PAE performed better than unweighted matrix UPGMA.

**Table 3:** The ordered ranking of the 16 analytical (weighting and clustering) approaches employed.

Matrix	Unweighted				Inverse				Bell				Integration			
Clustering	J	K2	S	PAE	J	K2	S	PAE	J	K2	S	PAE	J	K2	S	PAE
**# CoE endemics**	10	7	16	12	4	3	15	5	2	1	14	7	9	6	13	10
**# CoE Taxa**	14	12	16	15	6	2	13	7	4	1	5	9	8	3	11	10
**# CoEs**	15	11	14	16	11	2	5	11	5	4	2	9	9	1	5	8
**# of cells in CoEs**	14	13	16	15	8	3	6	9	7	1	2	10	10	4	4	12
**Sum of Ranking Score**	53	43	62	58	29	10	39	32	18	7	23	35	36	14	33	40
**Rank**	14	13	16	15	6	**2**	11	7	**4**	**1**	**5**	9	10	**3**	8	12

Rankings were based on the optimality values in Table 2. The Consensus CoEs / Sub-CoEs were excluded from the ranking assessment. J = Jaccard, K2 = Kulczinsky2, S = Simpson.

**Table 4:** The summed ranking of the different weighting techniques and similarity / clustering techniques employed.

	1^st^	2^nd^	3^rd^	4^th^
**Weighting**	Bell = 19	Inverse = 26	Integration = 33	Unweighted = 58
**Similarity / Clustering (incl. unweighted data)**	K2 = 6 (19)	J = 20 (34)	S = 24 (40)	PAE = 28 (43)

Summed ranking values were calculated from Table 3. Lower scores (summed placements) indicates a higher ranking. J = Jaccard, K2 = Kulczinsky2, S = Simpson.

**Fig 6:**
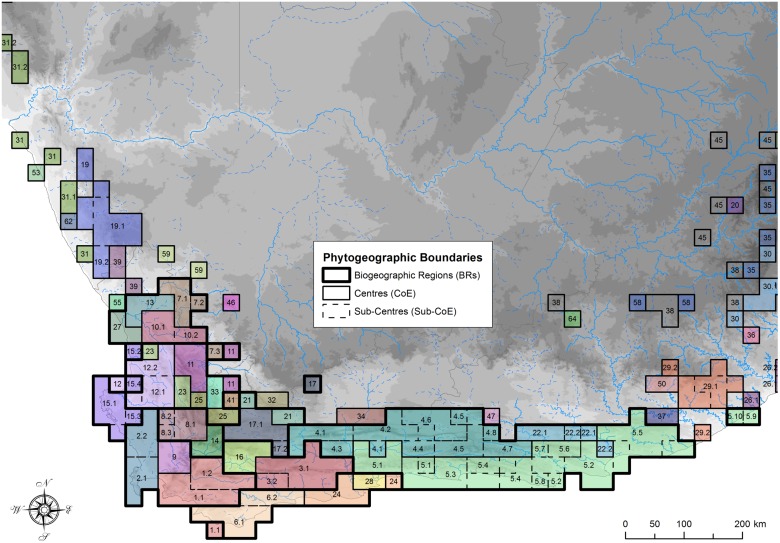
CFR centres of endemism, sub-centres of endemism and biogeographic regions retrieved. Names of CoEs, Sub-CoEs and BRs, as well as the geographic and taxonomic properties of these units are provided in S3 Table.

The Consensus analysis (Fig 1, step 7), although not ranked relative to the initial clustering analysis (Fig 1, step 3), gave the highest richness and endemism values, due to the incorporation of additional cells and the merging of overlapping CoEs from the three individual approaches (Table 3), which reduced the total numbers of CoEs (Table 2). With the increase in the number of cells assigned to CoEs following GIS interrogation, consensus CoEs approached BRs (Fig 6).

### Consensus and pattern robustness

**Fig 7:**
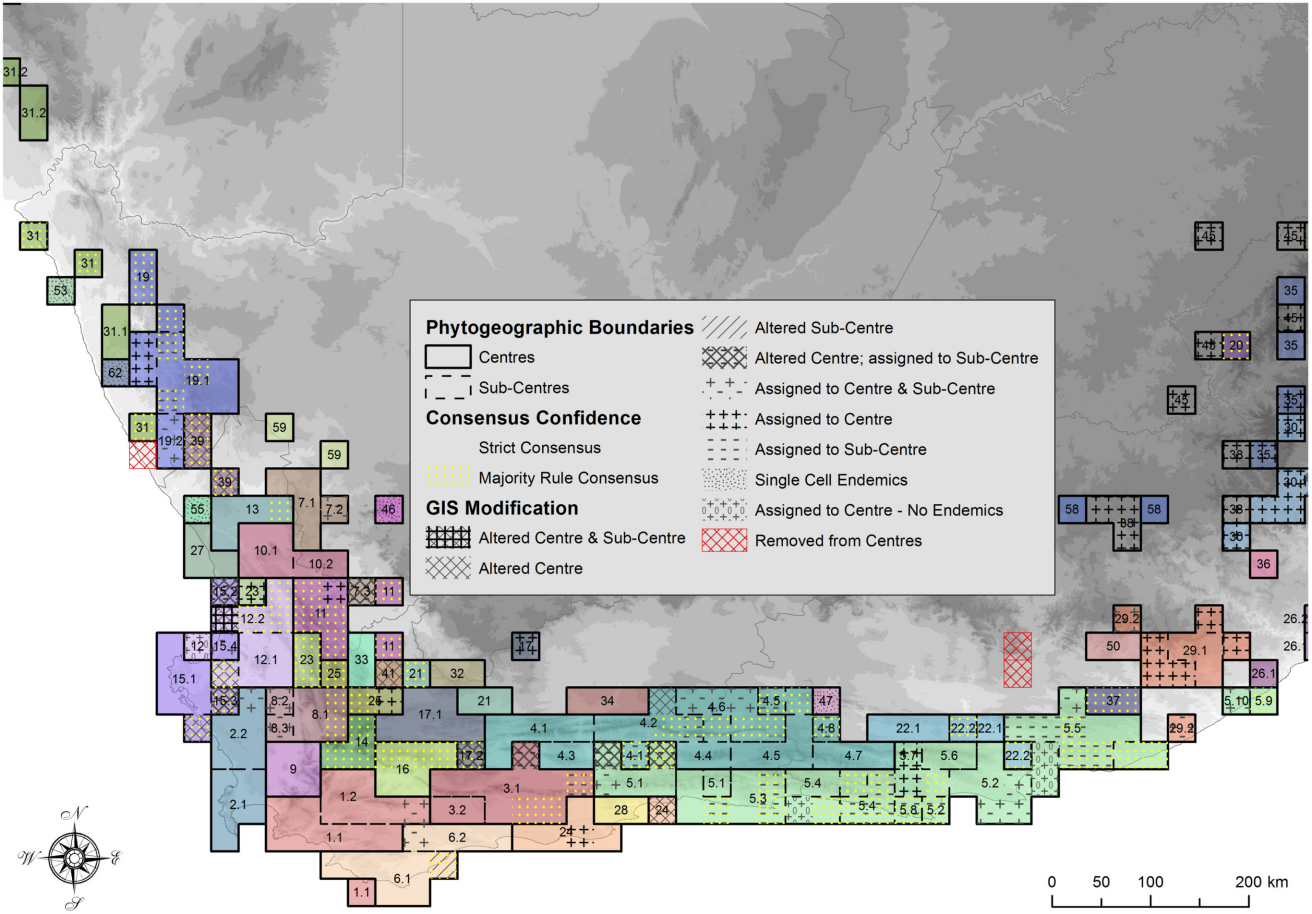
Spatial summary of the methods used for demarcating biogeographic areas. The relative robustness of the CoEs / Sub-CoEs identified in the CFR are indicated. The most robust CoEs / Sub-CoEs were retrieved by the strict consensus analysis (indicated without any patterning). These are followed in robustness by areas retrieved by the slightly less rigorous majority rule consensus (yellow dots). Post GIS modification then refined these initial candidates, altering cell assignment in CoEs (cross hatching) and Sub-CoEs (diagonal hatching). Cells not assigned due to conflict in clustering or consensus were then added to CoEs (+) or to Sub-CoEs (-), whereas single cell endemics that did not group with other cells were also indicated (fine dots). CoEs retrieved by clustering of taxa with very widespread distributions indicating uncertain collection effort were removed (red cross hatching). Finally, cells assigned to CoEs to bridge the gap between CoEs and BRs are indicated with +0, indicating no endemic taxa.

**Fig 8:**
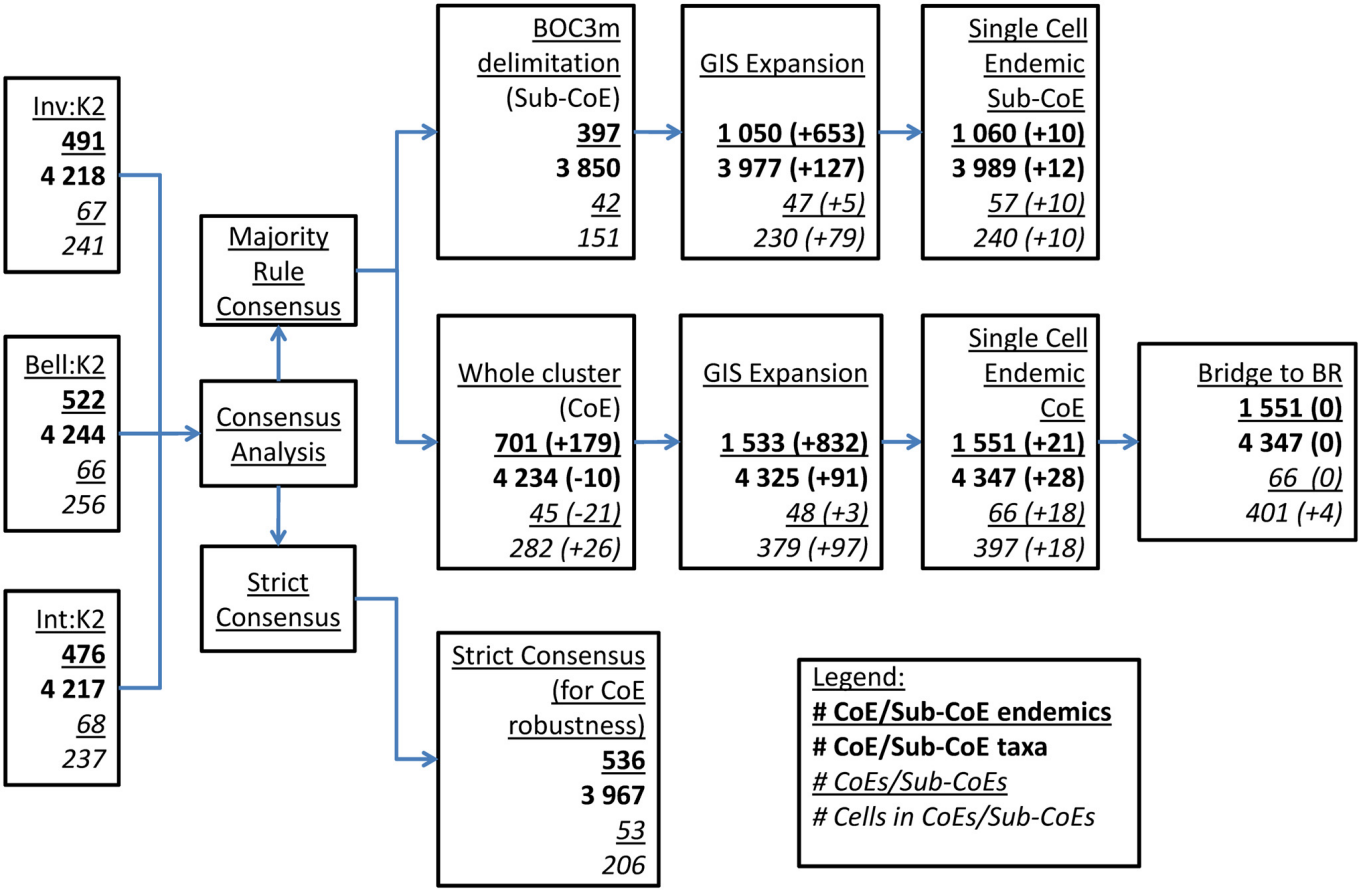
Flow chart summary of the change in CoE properties from clustering to GIS interrogation. CoE properties (endemic taxa, richness, number and size of CoEs / Sub-CoEs) of the best clustering technique results (Inv:K2, Bell:K2 & Int:K2), showing the effects of applying majority rule and strict consensus on CoE/Sub-CoE properties are depicted (the legend indicates what each number row refers to in each block). Also displayed are the improvements of CoE / Sub-CoE properties with GIS interrogation (the values in brackets indicate additional taxa or cells added to CoEs). Finally, the minimal changes required in CoE properties are reported when converting CoEs to BRs.

The spatial results of the strict and majority rule consensus trees of the Kulczinsky2 similarity coefficient clustering and GIS modifications (on Bell, Inv and Int weighting) are displayed in Fig 7. This indicates pattern robustness, where the clusters (CoEs) retained after strict consensus are most robust, followed by those retained in majority rule consensus, while CoEs retrieved from post clustering GIS interrogation are arguably the least robust. The taxonomic and spatial properties of CoEs during these intermediate steps are summarised in Fig 8. In areas with high numbers of narrow endemics, such as the southwest CFR (CoE 1, 2 & 3), CoEs were smaller and there was less conflict with phytogeographic boundaries, with most phytogeographic patterns retained in the strict consensus, indicating their robustness (Fig 7). To the east where there are fewer narrow endemics, CoEs were generally larger (CoE 4 & 5) with more variability in CoE boundaries; thus relatively fewer floristic units were retained in the strict consensus as compared to the majority rule consensus. Many of the inland central and northern CoEs of the CFR (CoE 11, 14, 16, 21, 23 & 25) showed some conflict in phytogeographic patterns, probably because they are small with lower levels of endemism and richness. Very little of this area was retrieved in the strict consensus analysis. Overall, the marginal increase in the numbers of endemics and cells in CoEs when using majority rule consensus was due to the merging of CoEs from the three individual level analyses (Fig 8), thus increasing CoE size but reducing CoE numbers.

### Implications of consensus and post-clustering GIS interrogation

There was a large increase in the numbers of endemics and size of CoEs with post clustering manual GIS manipulation and expansion of CoEs (Fig 8). GIS analysis also retrieved an appreciable number of single cell CoEs. The numbers of cells that needed to be added to bridge the gap between CoEs and BRs after clustering GIS analysis and expansion in the CFR was negligible (four cells), highlighting the abundance of endemic taxa throughout the CFR.

### Clustering hierarchy of CoE

**Fig 9:**
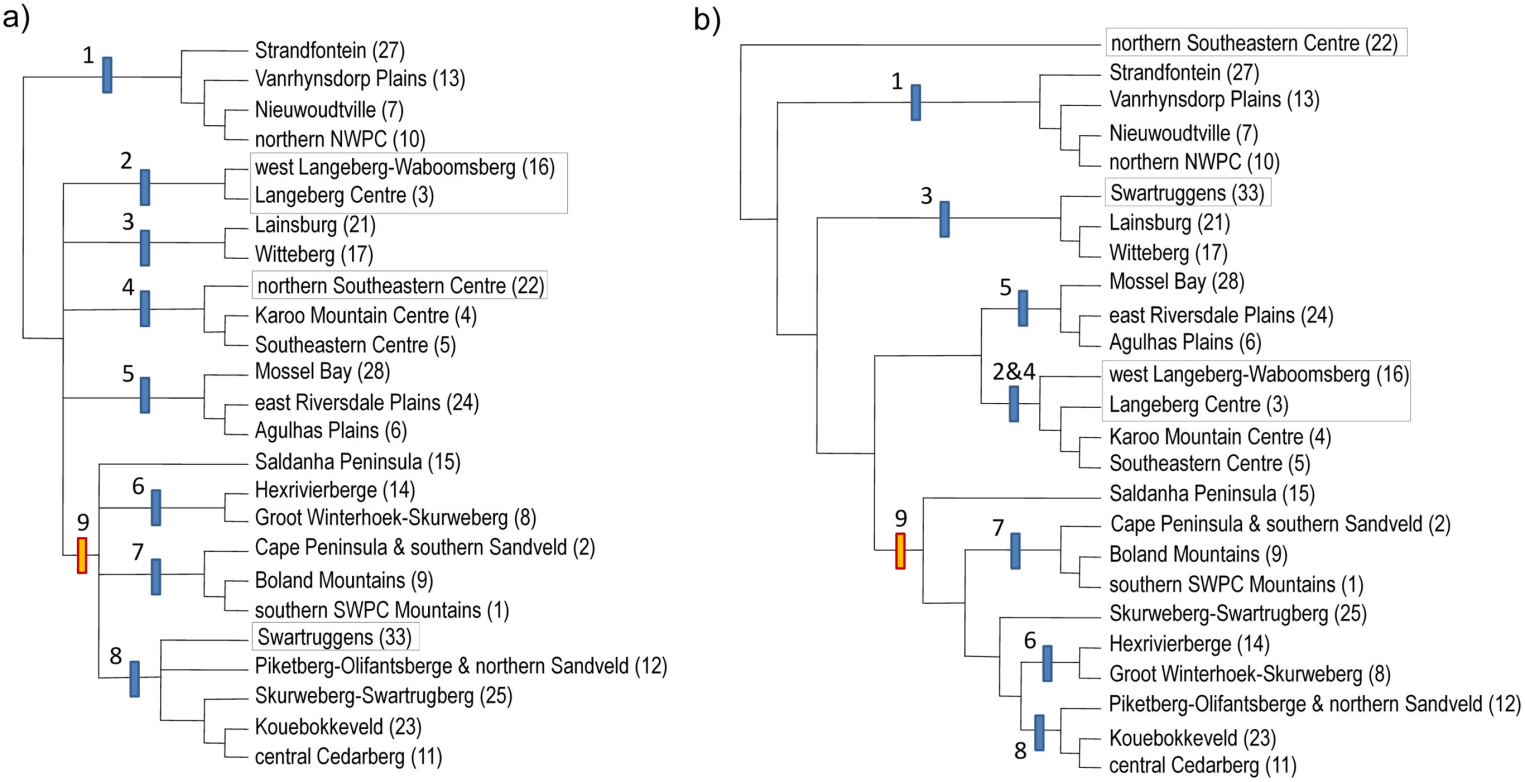
Consensus hierarchical clustering of core CFR CoE OGUs. Hierarchy clustering used Kulczinsky2 similarity (a) left pane) and Jaccard (b) right pane) each on the three weightings used in this study (Bell, Inverse and Integrated). BR numbers refer to: northern Northwest BR (1), Langeberg BR (2), Witteberg BR (3), East BR (4), Agulhas Plains BR (5), southern Northwest BR (6), Southwest BR (7), central Northwest BR (8), western CFR cluster (9). CoE whose positions differ between dendrograms are boxed. The results of Kulczinsky2 (5a) were used to delimit BRs in Fig 6.

**Fig 10:**
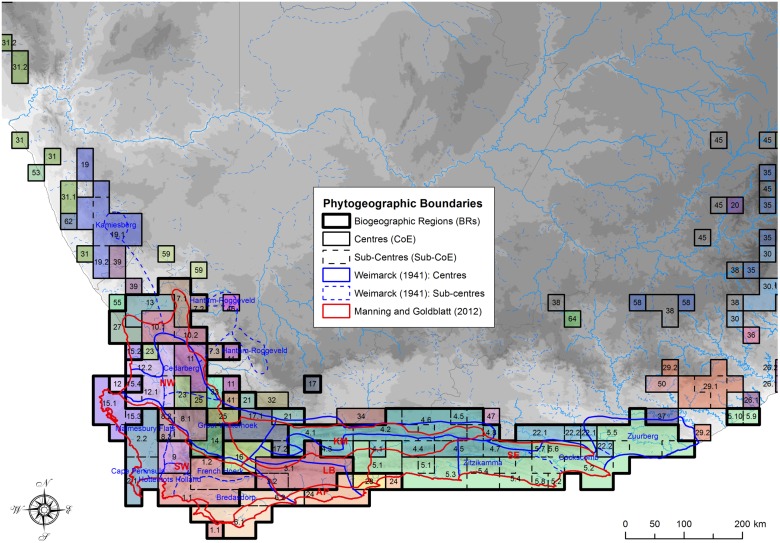
Our phytogeographic areas and those of earlier studies.

Hierarchical clustering using Jaccard and Kulczinsky2 indices produced broadly similar relationships between core CFR CoEs, indicating fairly robust patterns (Fig 9a & 9b); however, Jaccard retrieved greater internal dendrogram structure (Fig 9b). The western CoEs grouped into a larger Western area (cluster 9 in Fig 9a & 9b), while only Jaccard recorded hierarchical dendrogram structure in the east (Fig 9b). Both Kulczinsky2 and Jaccard depicted the northern Northwest BR (1) as terminal to the rest of the CFR (Fig 9a & 9b). Generally, the CoEs with lower levels of endemism and richness had the least consistent dendrogram placement, e.g. the northern Southeast CoE (22) and the Swartruggens (33), which may indicate ambiguous placement due to low taxon numbers, or being a transitional floristic area. The Agulhas Plains BR (5) and Witteberg BR (3) formed distinct BRs in both analyses. In the Kulczinsky2 analysis, the west Langeberg-Waboomsberg (16) and the Langeberg BR (3) formed a discrete cluster (Fig 9a), while in the Jaccard analysis they were sister to the Karoo Mountain BR (4) and Southeast BR cluster (5) (Fig 9b). The Saldanha Peninsula was basal to (Jaccard) or less associated with (Kulczinsky2) the Southwest BR (Figs 9a, 9b and 10).

### Phytogeographic classification of the CFR

A total of 66 CoEs and 57 Sub-CoEs were delimited in southern Africa (see S4 Table, S2 & S3 Figs), of which 25 CoEs and 44 Sub-CoEs occurred in the core area of the CFR (Fig 6, S3 Table). By comparison, Weimarck [26] demarcated six centres and 10 sub-centres, and Manning and Goldblatt [27] recognise six broad phytogeographic centres (Fig 10). There is still recognisable spatial congruence with these earlier classifications at higher hierarchical levels (Fig 10). For example, although the Saldanha Peninsula (15) did not cluster neatly with another CoE, it formed part of a nested western CFR cluster, which included most of the western CFR CoEs (SWBR, nNWBR, cNWBR & sNWBR) (Figs 9a, 9b & 10).

Whereas the Northern NWPC (CoE 10) had stronger floristic affinities to the Nieuwoudtville CoE (7) and clustered within the northern Northwest BR (BR 1) (Figs 9a, 9b and 6), it also has floristic affinities south to the Cederberg (11) and Olifantsberg (12) not apparent in this study (Fig 9a & 9b). Similarly, the Cape Peninsula (2.1) grouped more closely with the lower lying areas to the north (Sub-CoE 2.2), instead of the more montane Southern SWPC Mountains (1) (Fig 6).

## Discussion

### Weighting

We show that weighted data, irrespective of the similarity coefficient used, always performed better than unweighted data based on our optimality criteria for CoEs, corroborating earlier findings [8, 13, 32, 33]. We also show that Bell weighting was the most optimal weighting technique investigated (Table 4), followed by Inverse, then Integration weighting; however, all had relatively similar performance (Tables 2 & 3). Bell weighting [13] has been criticised for its *a posteriori* modification of variables [44], but in this study and elsewhere [83] it has been demonstrated that when optimal parameters (*a* and *p*) are already identified for a taxon (e.g. Restionaceae [13]), these parameters can be applied to other phytogeographically similar taxa. Although Int weighting is conceptually promising, adjusting to the properties of individual datasets, it did not perform as well as the Bell or Inv weighting methods. We do not know why its performance was weak. Improvements to the Int technique could potentially be realised by using more complex curves. Quantile intervals could also possibly be a simple and effective alternative frequency-based bin approach to subdivide the data for weighting, and could be explored further.

### Similarity measures

The top three performing individual analyses (Tables 2 & 3) all utilised the Kulczinsky2 similarity coefficient but with different weightings. Consequently, we infer that Kulczinsky2 performed better than the other similarity measures (Table 4), at least in our study, which focused on CoE retrieval.

Hennig and Hausdorf [114] reported that Kulczinsky2 was intermediate between Jaccard, where “the denominator is dominated by the more dispersed species” and is therefore potentially undesirable for CoE retrieval, and Simpson, which is more “extreme”, where the cell with fewer, presumably less dispersed, taxa dominates the coefficient. Both Jaccard and Kulczinsky2 are symmetric, but differ in that Jaccard calculates the overall proportion of shared taxa between two OGUs, whereas Kulczinsky2 calculates the arithmetic mean of the shared proportions of the two OGUs. Simpson is asymmetric and calculates the proportion of shared taxa of the least rich OGU. Thus, by taking the average, Kulczinsky2 may balance out differences in richness between OGUs.

While an assessment of similarity coefficient based purely on dendrogram topology may favour Jaccard over Kulczinsky2 [89], the better performance of Kulczinsky2 using our optimality criteria may indicate that more resolved dendrogram topologies may not necessarily translate into more desirable CoEs. As Kulczinsky2 takes an average of the two OGU similarity ratios, it is perhaps not too surprising that dendrogram topologies might be less resolved. Shi [48] assessed nine properties of 39 similarity measures, and reported that the suitability of Jaccard was high, Simpson moderate, and Kulczinsky2 low, contrary to Faith *et al*. [94] who found Kulczinsky2 to be robust as a compositional dissimilarity measure, as we did.

Although Simpson may account for differences in richness [2, 22, 92, 93] by using the ratio of the shared component “*a*” and the minimum of the non-shared taxon component (min(*b*,*c*)), if one OGU is completely nested in the other (*b* or *c*) a value of zero dissimilarity will be returned, even if the other OGU has many other taxa. This is potentially problematic for CoE retrieval if the less rich OGU is geographically peripheral and comprises mostly widespread taxa which it shares with more central and richer OGUs. Further, by favouring less rich OGUs, Simpson might be less optimal in the CFR where richness and endemism are highly correlated [83]. So, whereas Simpson is purely a “turnover” measure, Jaccard and Kulczinsky2 focus on taxonomic dissimilarity and may be more informative in certain biogeographic analyses [49, 115–117] such as the retrieval of CoEs. As there are noted differences in the performance of various similarity measures in the literature and in our study, we feel that it is important to apply the most optimal similarity measure for the question being addressed.

Our dendrogram comparison based on the correlation between dissimilarity matrices (Fig 5) indicated that the similarity measure employed had a more profound effect on the dissimilarity matrix than the weighting technique employed. Curiously, the fourth and fifth best performing analyses based on our optimality criteria (endemism, richness, CoE number and total CoE area), namely Bell:J and Bell:S, respectively (Table 3), are positioned relatively distantly from the top three performing Kulczinsky2 analyses, particularly Bell:J, in the correlation dendrogram (Fig 5). The reasons for the joint high ranking of dendrograms with apparently different structure are beyond the scope of this paper, but does caution against using dendrogram structure exclusively to assess performance of the similarity measure employed.

### Clustering techniques: UPGMA versus PAE

PAE was found to be the less optimal clustering algorithm for both weighted and unweighted approaches (Table 4), supporting previous findings [2, 13]. PAE’s poor performance may be due to the use of binary presence / absence of taxa to define clusters, thus there is more likely to be conflict in the placement of OGUs, resulting in a less resolved dendrogram. In contrast, the better-performing UPGMA uses similarity measures with many decimal places, making it much more sensitive to slight differences in similarity or very weak relationships between OGUs, thus giving a much more resolved dendrogram of OGU relationships. A noted inherent problem of cluster analysis is the forcing of samples into discrete groups, whether or not natural groupings exist, with increased distortion deeper in the dendrogram at lower similarity levels [48]. BOC3m, which mostly focuses on shallow or terminal clusters that have the greatest concentration of range-restricted taxa, avoids deeper dendrogram branches where distortion may be greater. Although PAE has easily definable candidate CoE [13], UPGMA with the BOC technique and GIS interrogation easily removes samples with no endemic taxa from clusters, resulting in more optimal CoEs, and is therefore recommended. Whereas previously PAE had the advantage of preferentially weighting taxa using the “WTS vector function” [109], it has been demonstrated that this can easily be replicated in UPGMA by duplicating taxa in the site by taxon matrix based on their weighting.

### Cluster identification—Branch Order Cut-off (BOC)

Using the BOC technique, candidate CoEs could be objectively and uniformly delimited in both UPGMA and PAE (i.e. dendrograms with and without similarity axes), which allowed for more objective comparisons of the performance of the different clustering algorithms.

The BOC method makes better use of the two dimensional structure of a dendrogram, which is itself a simplification of multidimensional topological relationships between OGUs, rather than subjectively reducing the complexity further by using a one dimensional phenon-line. As a result, OGUs were allocated on their individual merits, not on their relative strength in the entire dendrogram. Consequently, “weaker” clusters (lower similarity scores with lower numbers of range-restricted species) were retained, where endemic taxa were present.

The BOC method is more direct and time efficient than the L-method employed by Kreft and Jetz [2]. The BOC method is rule based but flexible; for example, standard Strahler scoring [59, 60] could be employed and order level cut-offs determined as required, as was undertaken here for BR identification (BOC2). Alternatively, order level calculations can be modified or adapted to suit particular datasets, as was employed here for CoE identification (BOC3m).

### Consensus

Although cumbersome, applying strict or majority rule consensus has the advantage of combining the differences in relationships between cells from different analytical approaches, rather than simply the optimal analysis. Furthermore, consensus of CoEs gives an indication of cluster robustness, indicating which cells are always assigned to the same cluster, which cells are assigned to a cluster the majority of the time and which cells are in conflict in all input analyses. Such conflict may be indicative of the presence of disparate biotic elements, or that the size or shape of cells are inappropriate at a given location, and highlights the need for further investigation of such areas or taxa.

### Further GIS analysis: Bridging the gap between CoEs and BRs in the CFR, and further afield

Using a GIS, cells not occurring in CoEs after majority rule consensus were assigned to CoEs, thereby increasing the size of CoEs and the numbers of taxa endemic to CoEs (Table 2 & Fig 8). This demonstrates that cluster analysis alone does not assign all possible cells to CoEs. Additionally, in the CFR, with the addition of just four cells without endemic taxa, we were able to bridge the gap between CoEs and BRs. This approach ensures that CoEs form the core of BRs, and that there is no conflict between the boundaries of CoEs and BRs.

In regions with fewer endemics, or where endemism is less widespread, CoEs could initially be established, followed by further clustering, treating the CoEs as larger OGUs together with the original unassigned cells, to investigate to which CoE the unassigned cells have the greatest affinity. Experimentation with lower endemism thresholds (e.g. taxa that are 90% endemic to a CoE) might also prove beneficial. As in this study, cells that contain no endemics could be indicated differently from those that do (Fig 7). Employing this approach can therefore delimit CoEs as a first important step, and then place these CoEs into regional biogeographic context by assigning non-CoE cells to CoEs with which they share the strongest similarity, resulting in all cells across the area of interest being allocated to BRs.

### Phytogeographic patterns in the CFR

Our phytogeographic boundaries show congruence with earlier floristic studies [26, 27] on the Cape flora (Fig 10); however, we retrieve additional phytogeographic detail and notable deviations, especially in the Northwest, Karoo Mountain, Agulhas Plains and Southeast Phytogeographic Centres. Further detail could potentially be achieved by analysing datasets partitioned by clade [83], or into different biotic elements (TMS versus shale taxa), or by utilising more natural OGUs such as eco-geographical areas [76].

### The Western CFR

The western CoEs (cluster 9 in Fig 9a & 9b) corresponding to the NWPC and SWPC of Manning and Goldblatt [27] (see Fig 6) are partitioned into numerous small CoEs. The northern and eastern boundaries of our Southwest BR (SWBR: BR 7 in Fig 9a & 9b) correspond largely with the SWPC boundary of Manning and Goldblatt [27], with boundaries along the non-sandstone intermontane valleys of the Berg and Breede Rivers. Our Southwest BR contains the highest levels of endemism and richness, and contained the two most endemic rich CoEs (Fig 6 & S3 Table). By comparison, the fairly mountainous Boland CoE (9) was not only geographically restricted in extent, but had relatively low endemism. This may indicate that the Boland CoE, while having its own endemic taxa, might also constitute a transitional area between a rich SWBR CoE (1) and a rich Northwest BR (NWBR) CoE (8). The high endemism of the Peninsula (S3 Table) may be due to its relatively large size here (*cf*. Weimarck [26]). This may be as a consequence of the relatively large cells analysed in this study (which may combine multiple habitats), resulting in the mountainous part of the Peninsula combining with the lower altitude vegetation rather than with the remaining southwest mountains, as shown by Moline and Linder [76] who used habitat specific units. Inspection of the majority consensus dendrogram reveals that the three more montane Peninsula cells form a cluster, as do the two lower altitude Sandveld cells (S1 Fig; see also Bradshaw [83]). Analysis using finer spatial input OGUs may result in a separation of the montane and lowland areas on the Peninsula, as mapped by Weimarck [26].

The Northwest BR (BR 8 in Fig 9a & 9b) is even more finely partitioned than the Southwest BR (Fig 6), with numerous CoEs centred on key landscape features, mostly mountain ranges. There is a southern group which includes the Groot Winterhoek and Hexrivier Mountains (CoEs 8 & 14), a more northerly group centred on the Cederberg (CoE 11), a group combining the Piketberg and Sandveld (12) to the west, and the Kouebokkeveld (23) to the south. The relationships of the Skurweberg-Swartrugberg (25) and the Swartruggens (33) are ambiguous between Kulczinsky2 and Jaccard (Fig 9a & 9b) and may represent transitional areas between the southern and northern NWBR groups, or between the CFR and more arid areas to the east (Fig 6).

We show, contrary to Manning and Goldblatt [27] and Weimarck [26], that the granitic Saldanha Peninsula (15) occupies an isolated position (Fig 9a & 9b), basal to both the SWBR and NWBR. Most Saldanha Peninsula endemics are geophytes with a few succulents, indicating lowland rather than mountain fynbos affinities (S1 Text).

Our data showed a distinct cluster of CoEs to the north of the CFR, the northern Northwest BR (nNWBR: BR 1 in Fig 9a & 9b), located around Nieuwoudtville. Our grouping of these as basal to the rest of the CFR has more in common with Weimarck [26], who largely excluded this area from his Northwest Centre, than with Manning and Goldblatt [27], who included the higher lying areas of this cluster into the CFR (Fig 10). It is likely a transitional area with higher altitude CFR biotic / floristic units and lower altitude Succulent Karoo geophytes and xeric elements, which cannot be differentiated at the cell resolution of our study. Similar reasoning can be applied to the Kamiesberg further north, where we retrieved no differentiation between these distinct floristic elements.

### The Eastern CFR

Southeast of the SWBR, we retrieved an Agulhas Plains BR (APBR, BR 5 in Fig 9a & 9b), similar to the Agulhas Plains Phytogeographic Centre of Manning and Goldblatt [27]. We found differentiation between the more coastal littoral, limestone areas and the more inland shale / sandstone areas (Fig 10). However, the Potberg (in the Potberg Sub-CoE 6.2), a notable coastal sandstone hill surrounded by littoral deposits on the Agulhas Plains (CoE 6), was distinct. We also retrieved a Langeberg BR (LBBR) comprising two CoE (BR 2 in Fig 9a & 9b), the easterly of which, the Langeberg (CoE 3), is spatially similar to that of both Manning and Goldblatt [27] and Weimarck [26]. The other CoE (16) extends further west than both previous studies (Fig 10). The LBBR is inconsistently grouped in the hierarchical analyses (Fig 9a & 9b), potentially indicating taxon conflict or a slightly weaker grouping. In the Kulczinsky2 analysis, the west Langeberg-Waboomsberg (16) and the Langeberg BR (3) formed a discrete cluster, while in the Jaccard analysis they were sister to the Karoo Mountain BR (4) and Southeast BR cluster (5). Our eastern boundary of the Langeberg CoE (3) on the Gourits River is closer to Weimarck [26] than the more easterly boundary of Manning and Goldblatt [27].

Two spatially large CoEs dominate the Eastern BR (EBR: BR 4 in Fig 9a & 9b). Inland, our Karoo Mountain CoE (4), centred on the Klein and Groot Swartberg, is similar to the delimitation in Manning and Goldblatt [27] and Weimarck [26], but here reduced in the west and extending in the east to include the Kammanassie and Baviaansberg, which Manning and Goldblatt [27] included in the Southeast CoE (Fig 10). However, their phytogeographic distinctiveness is partially retained by occurring in separate Sub-CoEs (Fig 10). We also retrieve additional phytogeographic detail at the Sub-CoE level not previously described in the KMBR, with intervals between montane floristic units caused by deeply incised river courses and intermontane basins.

Our Witteberg BR (BR 3 in Fig 9a & 9b), comprising the Witteberg CoE (17) and the Laingsburg CoE (21), might be transitional between the NWBR and KMBR, which may account for the discrepancies in phytogeographic boundaries between Manning and Goldblatt [27] and Weimarck [26] in this area (Fig 10).

Our Southeast CoE (CoE 5) was larger in extent than that of Manning and Goldblatt [27], extending from the Gourits River in the west to the Great Fish (with Suurberg Sub-CoE 5.5) in the east, covering the Knysna Interval and Southeast Centres of Weimarck [26] (Fig 10). Our sub-centre delimitations are loosely indicative of the sub-centres of Weimarck [26], but include additional phytogeographic detail (Fig 10). However, unlike Weimarck [26], we not only recorded Cape endemics in his Knysna Interval, but also retrieved distinct floristic development at sub-centre level—the West Outeniekwaberg (5.1) and the East Outeniekwaberg (5.3)—although with relatively few Cape (*sensu* Linder [66]) endemics (S1 Text). Further analysis with a more comprehensive list of Albany Thicket Biome [118] or Albany Centre [119] taxa may result in a westerly shift in the boundary for the eastern extent of the Southeast CoE (5).

### Implications for the CFR

Geographically smaller, more numerous CoEs in the western CFR (Fig 6) bear testimony to Levyns’ characteristic pattern of the highest levels of local endemism and richness in the southwest of the CFR, tapering off to the north and east [82] (Fig 6 & S3 Table), and to the high levels of beta and gamma diversity, together with congruent distributions of taxa there. Although this richness pattern is not immediately apparent when viewing the rankings of the CoEs and their levels of endemism, it emerges when CoE endemism is corrected for by CoE area. Thus, although the easterly Karoo Mountain Centre (CoE 4) is ranked fourth and the Southeast Centre (5) is ranked fifth, they are more than double and triple the size of the Southern SWPC Mountains CoE (1), respectively (Fig 6 & S3 Table). This corroborates the findings that species richness accumulates faster with increasing area in the western than eastern CFR [110, 120], indicative of higher beta and gamma diversity in the west [120].

## Conclusions

Weighted datasets outperformed unweighted datasets for CoE retrieval, with Bell weighting most optimal. The Kulczinsky2 coefficient outperformed Jaccard, and Simpson performed poorly. BOC is an objective technique to delimit CoE on dendrograms as well as phenograms, and UPGMA outperformed PAE. Strict and majority rule consensus established a robustness hierarchy of cells in CoEs. GIS manipulation enlarged CoE and included more endemic taxa, and helped bridge the step between CoE and BRs. Due to the success of the techniques employed, we recommend their consideration in regionalisation studies, either on their own or in conjunction with other disparate approaches (such as NDM or null models).

The successful retrieval of additional phytogeographic detail at various scales (BR, CoE & Sub-CoE) in the highly endemic rich CFR, and to a lesser extent their nestedness within recognised BRs (of Weimarck [26] and Manning and Goldblatt [27]), indicates the robustness and suitability of the techniques employed here. Therefore we recommend that existing phytogeographic boundaries of the CFR be revised in light of the current analysis.

## Supporting Information

S1 Fig Majority rule consensus tree of the three best individual trees (K2:Bell; K2:Inv & K2:Int). (PDF)

S2 Fig The location of all CoEs and Sub-CoEs retrieved in the study. CoE and Sub-CoE identified outside the CFR should be interpreted cautiously, as the dataset was biased to Cape clades (*sensu* Linder, 2003). To be used in conjunction with S4 Table.
(TIF)

S3 Fig The robustness of all CoEs and Sub-CoEs retrieved in the study. CoE and Sub-CoE identified outside the CFR should be interpreted cautiously, as the dataset was biased to Cape clades (*sensu* Linder, 2003). To be used in conjunction with S4 Table.
(TIF)

S1 Table Contact details of dataset owners. Please note that some of the datasets have been cleaned and updated by the authors during the analysis, and some datasets may have been by the source since the analysis was undertaken, and if requested from the source, may not be identical to the datasets analysed in the study. However, if permission can be obtained from the listed source, the specific data analysed here could be provided on request.
(DOCX)

S2 Table The correlation values between the 12 dissimilarity matrices. A pair-wise Mantel Test (with Pearson correlation and 999 permutations) of the 12 different dissimilarity matrices indicated that all correlations were significant with p = 0.001.
(DOCX)

S3 Table The taxonomic and geographic properties of CoEs and Sub-CoEs occurring in the core CFR. Labels correspond to map CoE / Sub-CoE labels (Fig 6). “Remainder” Sub-CoEs refer to CoE cells not assigned to Sub-CoEs, and are not necessarily geographically continuous. Cells = size of the CoE (number of cells); Taxa = the total number of taxa represented in a CoE; 50% End = number of taxa with at least half their ranges within the CoE; Ends = total number of taxa endemic to a CoE/Sub-CoE; BR = Biogeographic Regions* indentified in this study (Fig 6). Biogeographic Regions (BR) abbreviations are as follows: SWBR = Southwest BR, LBBR = Langeberg BR, EBR = East BR, APBR = Agulhas Plains BR, nNWBR = northern Northwest BR, sNWBR = southern Northwest BR, cNWBR = central Northwest BR, WBBR = Witteberg BR. Extra-CFR CoEs retrieved for the entire dataset analysed can be found in the Supporting Information (S2 Table, S2 & S3 Figs).
(DOCX)

S4 Table The complete list of CoEs and Sub-CoEs and their properties retrieved in the study. As the dataset was biased to Cape clades (*sensu* Linder, 2003), interpretation of extra-CFR CoE should be limited to where potential extra-Cape CoEs for Cape taxa might be located, as the dataset examined does not comprise the entire compliment of Cape Clades. Further, interpretation of national CoEs in a national context would require a floristically unbiased national dataset. To be used in conjunction with S2 Fig.
(DOCX)

S1 Text The list of taxa endemic to all the CoEs and Sub-CoEs identified. (DOCX)
