# Supplementary material for: Optimising Regionalisation Techniques: Identifying Centres of Endemism in the Extraordinarily Endemic-Rich Cape Floristic Region
Source: PLoS One. 2015 Jul 6;10(7):e0132538. doi: 10.1371/journal.pone.0132538 (PMC4493007; doi:10.1371/journal.pone.0132538)

S1 Fig. Majority rule consensus tree of the three best performing individual dendrograms (K2:Bell, K2:Inv & K2:Int).

Legend

| indicates centre demarcation

| indicates sub-centre demarcation based on BOC3m method

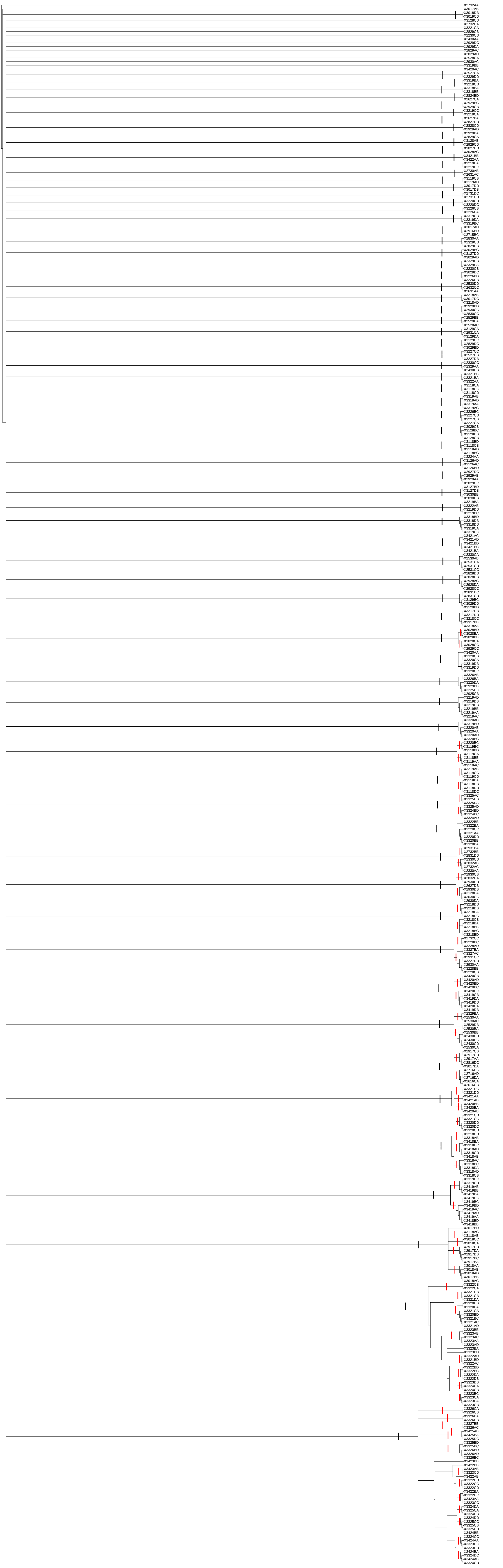

Supplement: S1 Fig — (PDF) [file pone.0132538.s001.pdf]
