# Supplementary figures and images for: Optimising Regionalisation Techniques: Identifying Centres of Endemism in the Extraordinarily Endemic-Rich Cape Floristic Region
Source: PLoS One. 2015 Jul 6;10(7):e0132538. doi: 10.1371/journal.pone.0132538 (PMC4493007; doi:10.1371/journal.pone.0132538)

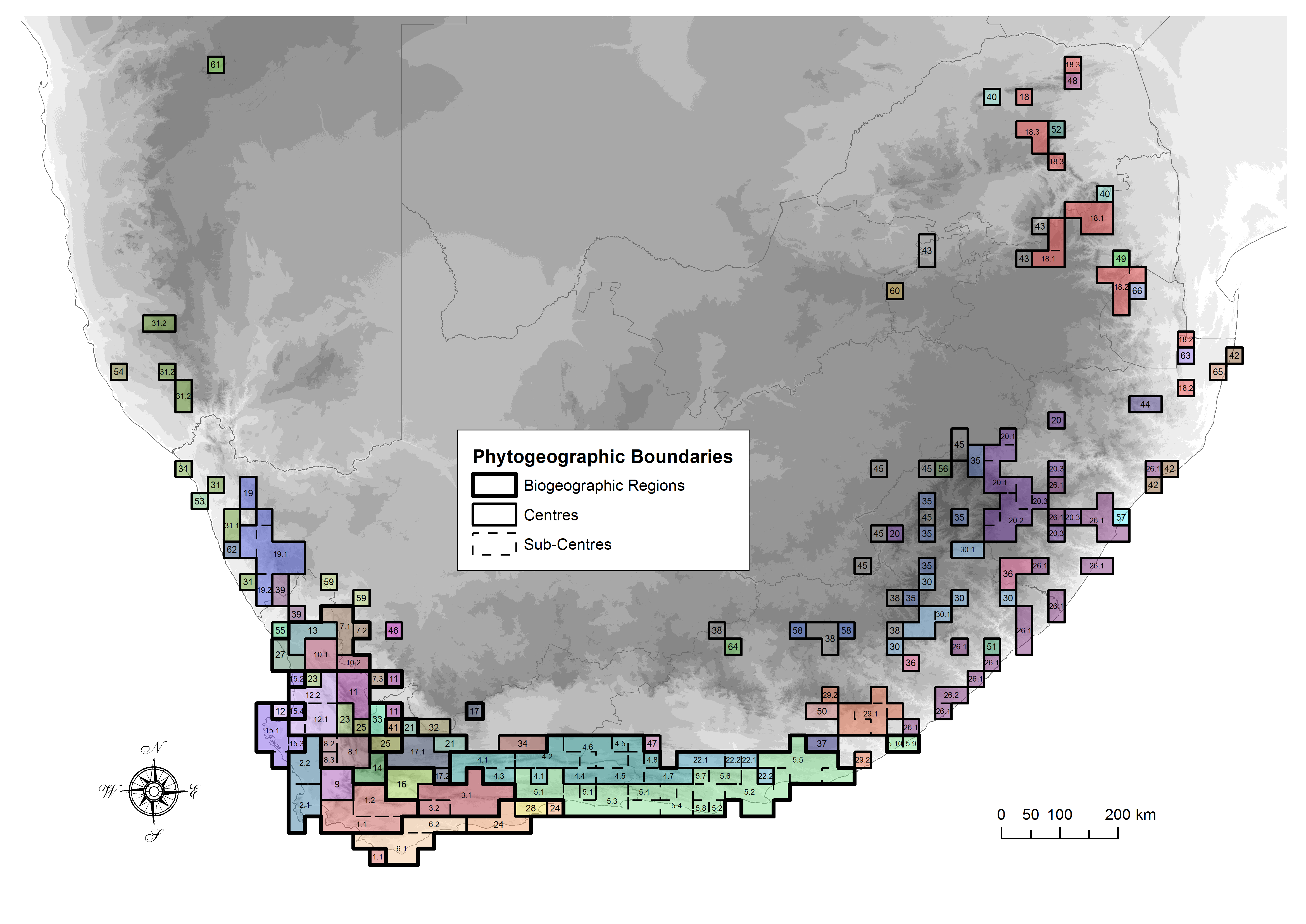

Supplement: S2 Fig — CoE and Sub-CoE identified outside the CFR should be interpreted cautiously, as the dataset was biased to Cape clades (sensu Linder, 2003). To be used in conjunction with S4 Table. (TIF) [file pone.0132538.s002.tif]

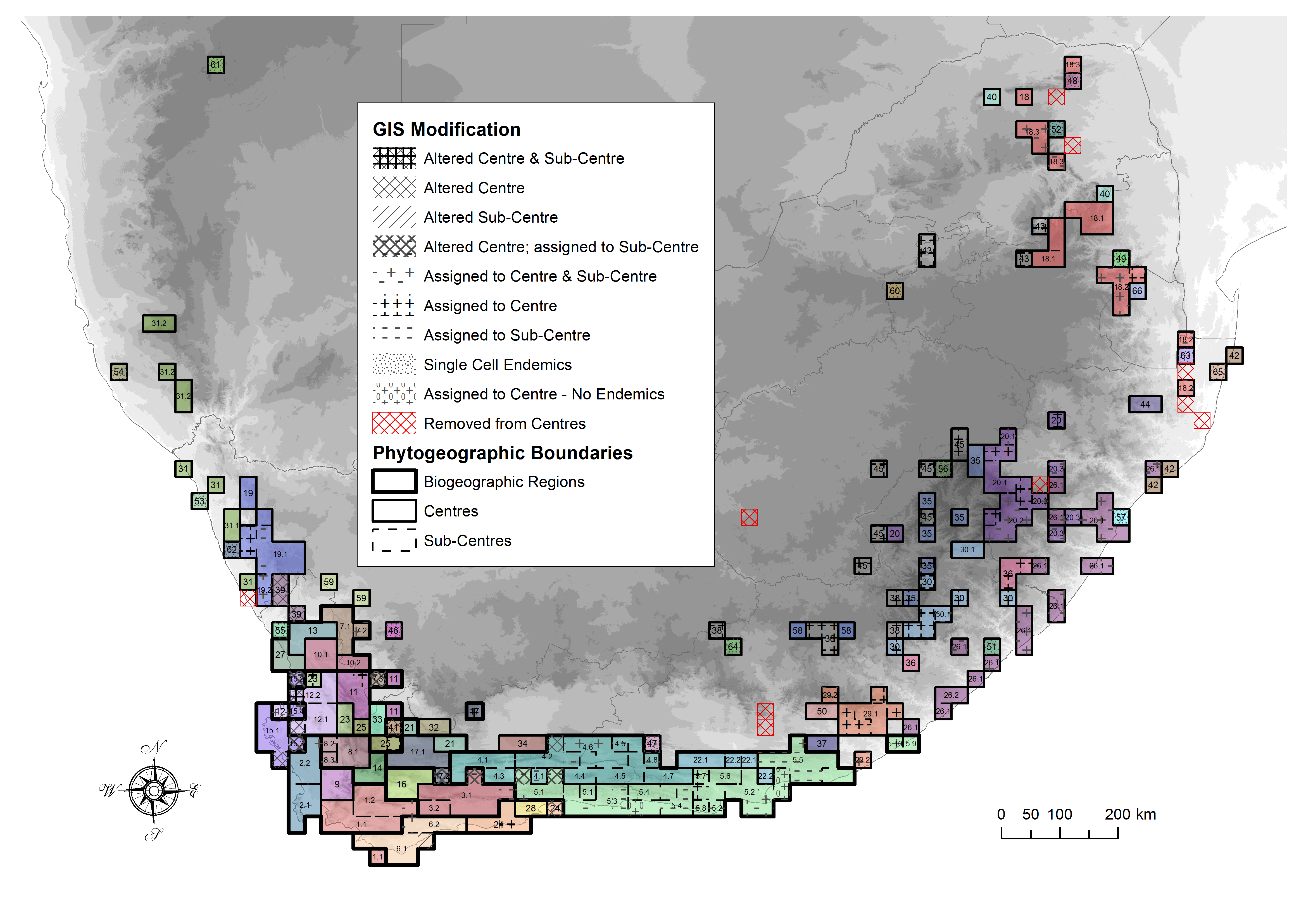

Supplement: S3 Fig — CoE and Sub-CoE identified outside the CFR should be interpreted cautiously, as the dataset was biased to Cape clades (sensu Linder, 2003). To be used in conjunction with S4 Table. (TIF) [file pone.0132538.s003.tif]
