# Supplementary material for: Optimising Regionalisation Techniques: Identifying Centres of Endemism in the Extraordinarily Endemic-Rich Cape Floristic Region
Source: PLoS One. 2015 Jul 6;10(7):e0132538. doi: 10.1371/journal.pone.0132538 (PMC4493007; doi:10.1371/journal.pone.0132538)
Supplement: S1 Table — Please note that some of the datasets have been cleaned and updated by the authors during the analysis, and some datasets may have been by the source since the analysis was undertaken, and if requested from the source, may not be identical to the datasets analysed in the study. However, if permission can be obtained from the listed source, the specific data analysed here could be provided on request. (DOCX) [file pone.0132538.s004.docx]

**S1 Table. Contact details of dataset owners.** Please note that some of the datasets have been cleaned and updated by the authors during the analysis, and some datasets may have been by the source since the analysis was undertaken, and if requested from the source, may not be identical to the datasets analysed in the study. However, if permission can be obtained from the listed source, the specific data analysed here could be provided on request.

| Family | Taxon | Source | email |
| --- | --- | --- | --- |
| Asteraceae | Endemic genera  Red Data List taxa | Peter Bradshaw  Red Data List | [peter.bradshaw@nmmu.ac.za](mailto:peter.bradshaw@nmmu.ac.za)  <http://redlist.sanbi.org/index.php> |
| Bruniaceae | Various | OLR^a^, Ted Oliver | [eoliver@sun.ac.za](mailto:eoliver@sun.ac.za) |
| Ericaceae | *Erica* | Ted Oliver | [eoliver@sun.ac.za](mailto:eoliver@sun.ac.za) |
| Fabaceae | *Aspalathus*  Red Data List taxa | OLR^a^, Ted Oliver  Red Data List | [eoliver@sun.ac.za](mailto:eoliver@sun.ac.za)  <http://redlist.sanbi.org/index.php> |
| Geophytes | Red Data List taxa  *Ferraria*, *Haemanthus* | Red Data List  Peter Bradshaw | [peter.bradshaw@nmmu.ac.za](mailto:peter.bradshaw@nmmu.ac.za) |
| Minor near endemic Families | various (e.g. Geissolomataceae, Grubbiaceae, Roridulaceae, and Stilbaceae) | OLR^a^, Ted Oliver | [eoliver@sun.ac.za](mailto:eoliver@sun.ac.za) |
| Orchidaceae | Various | Peter Linder | [peter.linder@systbot.uzh.ch](mailto:peter.linder@systbot.uzh.ch) |
| Poaceae | Various | Peter Linder | [peter.linder@systbot.uzh.ch](mailto:peter.linder@systbot.uzh.ch) |
| Proteaceae | Various | Protea Atlas Project, Tony Rebelo | [T.Rebelo@sanbi.org.za](mailto:T.Rebelo@sanbi.org.za) |
| Red Data list Taxa | Various | <http://redlist.sanbi.org/index.php> | <http://redlist.sanbi.org/index.php> |
| Restionaceae | African Restionaceae | Peter Linder | [peter.linder@systbot.uzh.ch](mailto:peter.linder@systbot.uzh.ch) |
| Rosaceae | *Cliffortia* | Peter Linder, Christopher Whitehouse | [peter.linder@systbot.uzh.ch](mailto:peter.linder@systbot.uzh.ch) |
| Rutaceae | *Agathosma*  Smaller genera (*sans* *Agathosma)* | Terry Trinder Smith  Peter Bradshaw | [terry.trinder-smith@uct.ac.za](mailto:terry.trinder-smith@uct.ac.za)  [peter.bradshaw@nmmu.ac.za](mailto:peter.bradshaw@nmmu.ac.za) |

**References**

^a^ OLR = Oliver EGH, Linder HP, Rourke JP (1983) Geographical distribution of present-day Cape taxa and their phytogeographical significance. *Bothalia* 14: 427-440.
