# Supplementary material for: Optimising Regionalisation Techniques: Identifying Centres of Endemism in the Extraordinarily Endemic-Rich Cape Floristic Region
Source: PLoS One. 2015 Jul 6;10(7):e0132538. doi: 10.1371/journal.pone.0132538 (PMC4493007; doi:10.1371/journal.pone.0132538)
Supplement: S2 Table — A pair-wise Mantel Test (with Pearson correlation and 999 permutations) of the 12 different dissimilarity matrices indicated that all correlations were significant with p = 0.001. (DOCX) [file pone.0132538.s005.docx]

**S2 Table. The correlation values between the 12 dissimilarity matrices.** A pair-wise Mantel Test (with Pearson correlation and 999 permutations) of the 12 different dissimilarity matrices indicated that all correlations were significant with p = 0.001.

|  | Bell:J | Bell:K2 | Bell:S | Int:J | Int:K2 | Int:S | Inv:J | Inv:K2 | Inv:S | Unw:J | Unw:K2 | Unw:S |
| --- | --- | --- | --- | --- | --- | --- | --- | --- | --- | --- | --- | --- |
| Bell:J | - | - | - | - | - | - | - | - | - | - | - | - |
| Bell:K2 | 0.62 | - | - | - | - | - | - | - | - | - | - | - |
| Bell:S | 0.4 | 0.95 | - | - | - | - | - | - | - | - | - | - |
| Int:J | 0.96 | 0.6 | 0.39 | - | - | - | - | - | - | - | - | - |
| Int:K2 | 0.61 | 0.97 | 0.92 | 0.63 | - | - | - | - | - | - | - | - |
| Int:S | 0.4 | 0.93 | 0.97 | 0.41 | 0.95 | - | - | - | - | - | - | - |
| Inv:J | 0.98 | 0.61 | 0.4 | 0.99 | 0.62 | 0.4 | - | - | - | - | - | - |
| Inv:K2 | 0.61 | 0.99 | 0.94 | 0.62 | 0.99 | 0.94 | 0.62 | - | - | - | - | - |
| Inv:S | 0.4 | 0.94 | 0.99 | 0.4 | 0.94 | 0.99 | 0.4 | 0.95 | - | - | - | - |
| Unw:J | 0.83 | 0.58 | 0.38 | 0.88 | 0.6 | 0.4 | 0.87 | 0.6 | 0.4 | - | - | - |
| Unw:K2 | 0.56 | 0.86 | 0.79 | 0.58 | 0.89 | 0.82 | 0.58 | 0.89 | 0.82 | 0.69 | - | - |
| Unw:S | 0.38 | 0.83 | 0.85 | 0.39 | 0.85 | 0.88 | 0.39 | 0.85 | 0.87 | 0.47 | 0.95 | - |
