# Supplementary material for: Optimising Regionalisation Techniques: Identifying Centres of Endemism in the Extraordinarily Endemic-Rich Cape Floristic Region
Source: PLoS One. 2015 Jul 6;10(7):e0132538. doi: 10.1371/journal.pone.0132538 (PMC4493007; doi:10.1371/journal.pone.0132538)
Supplement: S3 Table — Labels correspond to map CoE / Sub-CoE labels (Fig 6). “Remainder” Sub-CoEs refer to CoE cells not assigned to Sub-CoEs, and are not necessarily geographically continuous. Cells = size of the CoE (number of cells); Taxa = the total number of taxa represented in a CoE; 50% End = number of taxa with at least half their ranges within the CoE; Ends = total number of taxa endemic to a CoE/Sub-CoE; BR = Biogeographic Regions* indentified in this study (Fig 6). Biogeographic Regions (BR) abbreviations are as follows: SWBR = Southwest BR, LBBR = Langeberg BR, EBR = East BR, APBR = Agulhas Plains BR, nNWBR = northern Northwest BR, sNWBR = southern Northwest BR, cNWBR = central Northwest BR, WBBR = Witteberg BR. Extra-CFR CoEs retrieved for the entire dataset analysed can be found in the Supporting Information (S2 Table, S2 & S3 Figs). (DOCX) [file pone.0132538.s006.docx]

S3 Table. The taxonomic and geographic properties of CoEs and Sub-CoEs occurring in the core CFR. Labels correspond to map CoE / Sub-CoE labels (Fig 6). “Remainder” Sub-CoEs refer to CoE cells not assigned to Sub-CoEs, and are not necessarily geographically continuous. Cells = size of the CoE (number of cells); Taxa = the total number of taxa represented in a CoE; 50% End = number of taxa with at least half their ranges within the CoE; Ends = total number of taxa endemic to a CoE/Sub-CoE; BR = Biogeographic Regions* indentified in this study (Fig 6). Biogeographic Regions (BR) abbreviations are as follows: SWBR = Southwest BR, LBBR = Langeberg BR, EBR = East BR, APBR = Agulhas Plains BR, nNWBR = northern Northwest BR, sNWBR = southern Northwest BR, cNWBR = central Northwest BR, WBBR = Witteberg BR. Extra-CFR CoEs retrieved for the entire dataset analysed can be found in the Supporting Information (S4 Table, S2 Fig & S3 Fig).

| **Label** | **CoE name** | **Cells** | **Taxa** | **50% End** | **Ends** | **BR*** |
| --- | --- | --- | --- | --- | --- | --- |
| **1** | Southern SWPC Mountains | 14 | 1717 | 769 | 301 | SWBR |
| **1.1** | Hottentots-Holland - Kleinrivierberg | 8 | 1383 | 1383 | 160 | SWBR |
| **1.2** | Riviersonderendberg | 6 | 1061 | 1061 | 79 | SWBR |
| **2** | Cape Peninsula and Southern Sandveld | 10 | 981 | 295 | 137 | SWBR |
| **2.1** | Cape Peninsula | 5 | 891 | 891 | 99 | SWBR |
| **2.2** | Southern Sandveld | 5 | 354 | 354 | 22 | SWBR |
| **3** | Langeberg Centre | 12 | 868 | 230 | 135 | LBBR |
| **3.1** | Langeberg | 10 | 806 | 806 | 112 | LBBR |
| **3.2** | West Riversdale Plains | 2 | 283 | 283 | 7 | LBBR |
| **4** | Karoo Mountain Centres | 31 | 720 | 251 | 130 | EBR |
| **4.1** | Klein Swartberg-Touwsberg | 6 | 366 | 366 | 34 | EBR |
| **4.2** | Groot Swartberg | 5 | 324 | 324 | 23 | EBR |
| **4.3** | Rooiberg | 2 | 168 | 168 | 13 | EBR |
| **4.4** | Kammanassieberg | 2 | 168 | 168 | 10 | EBR |
| **4.5** | Kougaberg-West Baviaanskloof | 5 | 238 | 238 | 7 | EBR |
| **4.6** | Slypsteenberg-Antoniesberg | 5 | 103 | 103 | 6 | EBR |
| **4.7** | East Baviaansberg | 3 | 152 | 152 | 4 | EBR |
| **4.8** | North Baviaanskloof | 1 | 21 | 21 | 1 | EBR |
| **4.9** | Remainder | 2 | 43 | 0 | 0 | EBR |
| **5** | Southeastern Centre | 48 | 805 | 285 | 122 | EBR |
| **5.1** | West Outeniekwaberg | 6 | 461 | 461 | 24 | EBR |
| **5.2** | Port Elizabeth Peninsula | 7 | 306 | 306 | 24 | EBR |
| **5.3** | East Outeniekwaberg | 7 | 379 | 379 | 12 | EBR |
| **5.4** | Tsitsikammaberg | 5 | 337 | 337 | 10 | EBR |
| **5.5** | West Albany Centre | 8 | 167 | 167 | 7 | EBR |
| **5.6** | Groot-Winterhoekberge | 2 | 156 | 156 | 4 | EBR |
| **5.7** | Cockscomb | 1 | 60 | 60 | 3 | EBR |
| **5.8** | Oesterbaai | 1 | 64 | 64 | 1 | EBR |
| **5.9** | East London | 1 | 15 | 15 | 1 | EBR |
| **5.10** | Kiwane | 1 | 7 | 7 | 1 | EBR |
| **5.11** | Remainder (includes 3 cells: CoE to BR) | 9 | 219 | 2 | 0 | EBR |
| **6** | Agulhas Plains | 11 | 758 | 224 | 101 | APBR |
| **6.1** | West Agulhas Plains | 7 | 624 | 624 | 55 | APBR |
| **6.2** | Potberg | 4 | 349 | 349 | 28 | APBR |
| **7** | Nieuwoudtville | 6 | 275 | 103 | 74 | nNWBR |
| **7.1** | Nieuwoudtville Core | 4 | 264 | 98 | 69 | nNWBR |
| **7.2** | Rooiberg | 1 | 12 | 4 | 2 | nNWBR |
| **7.3** | Central Tankwa | 1 | 7 | 1 | 1 | nNWBR |
| **8** | Groot-Winterhoek - Skurweberg | 6 | 924 | 155 | 70 | sNWBR |
| **8.1** | Groot-Winterhoek - Skurweberg Core | 4 | 888 | 888 | 64 | sNWBR |
| **8.2** | Heuningberg | 1 | 78 | 3 | 1 | sNWBR |
| **8.3** | Kasteelberg | 1 | 86 | 1 | 1 | sNWBR |
| **9** | Boland Mountains | 4 | 1072 | 152 | 50 | SWBR |
| **10** | Northern NWPC | 6 | 281 | 87 | 48 | nNWBR |
| **10.1** | Gifberg-Matsikammaberg | 4 | 256 | 256 | 41 | nNWBR |
| **10.2** | Boegoeberge | 2 | 61 | 61 | 6 | nNWBR |
| **11** | Central Cederberg | 7 | 596 | 119 | 44 | cNWBR |
| **12** | Piketberg/Olifantsberge and Northern Sandveld | 10 | 540 | 105 | 40 | cNWBR |
| **12.1** | Piketberg | 4 | 379 | 379 | 24 | cNWBR |
| **12.2** | North Sandveld | 4 | 324 | 324 | 9 | cNWBR |
| **12.3** | Remainder (includes 1 cell: CoE to BR) | 2 | 29 | 0 | 0 | cNWBR |
| **13** | Vanrhynsdorp Plains | 4 | 80 | 43 | 33 | nNWBR |
| **14** | Hexrivierberge | 3 | 661 | 65 | 25 | sNWBR |
| **15** | Saldanha Peninsula | 9 | 220 | 40 | 25 | In wCFR |
| **15.1** | Saldanha Core | 5 | 100 | 100 | 14 | In wCFR |
| **15.2** | Lambert's Bay | 1 | 42 | 8 | 3 | In wCFR |
| **15.3** | Hopefield | 1 | 110 | 4 | 1 | In wCFR |
| **15.4** | Aurora | 1 | 42 | 1 | 1 | In wCFR |
| **15.5** | Remainder | 1 | 37 | 0 | 0 | In wCFR |
| **16** | West Langeberg - Waboomsberg | 5 | 362 | 41 | 21 | LBBR |
| **17** | Witteberg | 8 | 364 | 43 | 17 | WBBR |
| **17.1** | Witteberg Core | 6 | 336 | 336 | 14 | WBBR |
| **17.2** | Towerkop | 1 | 87 | 2 | 1 | WBBR |
| **17.3** | Remainder | 1 | 4 | 1 | 0 | WBBR |
| **21** | Laingsberg | 3 | 99 | 21 | 13 | WBBR |
| **22** | Northern Southeastern Centre | 6 | 80 | 18 | 13 | EBR |
| **22.1** | Wolwefontein | 4 | 58 | 58 | 9 | EBR |
| **22.2** | Sunday's River Valley | 2 | 31 | 31 | 3 | EBR |
| **23** | Kouebokkeveld | 3 | 532 | 46 | 12 | cNWBR |
| **24** | East Riversdale Plains | 6 | 263 | 37 | 12 | APBR |
| **25** | Skurweberg - Swartrugberg | 3 | 333 | 28 | 9 | cNWBR |
| **27** | Strandfontein | 3 | 34 | 14 | 7 | nNWBR |
| **28** | Mossel Bay | 2 | 135 | 8 | 4 | APBR |
| **33** | Swartruggens | 2 | 87 | 6 | 2 | cNWBR |
