# Supplementary material for: Optimising Regionalisation Techniques: Identifying Centres of Endemism in the Extraordinarily Endemic-Rich Cape Floristic Region
Source: PLoS One. 2015 Jul 6;10(7):e0132538. doi: 10.1371/journal.pone.0132538 (PMC4493007; doi:10.1371/journal.pone.0132538)
Supplement: S4 Table — As the dataset was biased to Cape clades (sensu Linder, 2003), interpretation of extra-CFR CoE should be limited to where potential extra-Cape CoEs for Cape taxa might be located, as the dataset examined does not comprise the entire compliment of Cape Clades. Further, interpretation of national CoEs in a national context would require a floristically unbiased national dataset. To be used in conjunction with S2 Fig. (DOCX) [file pone.0132538.s007.docx]

**S4 Table. The complete list of CoEs and Sub-CoEs and their properties retrieved in the study.** As the dataset was biased to Cape clades (*sensu* Linder, 2003), interpretation of extra-CFR CoE should be limited to where potential extra-Cape CoEs for Cape taxa might be located, as the dataset examined does not comprise the entire compliment of Cape Clades. Further, interpretation of national CoEs in a national context would require a floristically unbiased national dataset. To be used in conjunction with S2 Fig.

| **Label** | **CoE** | **Cells** | **Taxa** | **50** | **Ends** |
| --- | --- | --- | --- | --- | --- |
| **1** | Southern SWPC Mountains | 14 | 1717 | 769 | 301 |
| **1.1** | Hottentots-Holland - Kleinrivierberg | 8 | 1383 | 1383 | 160 |
| **1.2** | Riviersonderendberg | 6 | 1061 | 1061 | 79 |
| **2** | Cape Peninsula and Southern Sandveld | 10 | 981 | 295 | 137 |
| **2.1** | Cape Peninsula | 5 | 891 | 891 | 99 |
| **2.2** | Southern Sandveld | 5 | 354 | 354 | 22 |
| **3** | Langeberg Centre | 12 | 868 | 230 | 135 |
| **3.1** | Langeberg | 10 | 806 | 806 | 112 |
| **3.2** | West Riversdale Plains | 2 | 283 | 283 | 7 |
| **4** | Karoo Mountain Centres | 31 | 720 | 251 | 130 |
| **4.1** | Klein Swartberg-Touwsberg | 6 | 366 | 366 | 34 |
| **4.2** | Groot Swartberg | 5 | 324 | 324 | 23 |
| **4.3** | Rooiberg | 2 | 168 | 168 | 13 |
| **4.4** | Kammanassieberg | 2 | 168 | 168 | 10 |
| **4.5** | Kougaberg-West Baviaanskloof | 5 | 238 | 238 | 7 |
| **4.6** | Slypsteenberg-Antoniesberg | 5 | 103 | 103 | 6 |
| **4.7** | East Baviaansberg | 3 | 152 | 152 | 4 |
| **4.8** | North Baviaanskloof | 1 | 21 | 21 | 1 |
| **4.9** | Remainder | 2 | 43 | 0 | 0 |
| **5** | Southeastern Centre | 48 | 805 | 285 | 122 |
| **5.1** | West Outeniekwaberg | 6 | 461 | 461 | 24 |
| **5.2** | Port Elizabeth Peninsula | 7 | 306 | 306 | 24 |
| **5.3** | East Outeniekwaberg | 7 | 379 | 379 | 12 |
| **5.4** | Tsitsikammaberg | 5 | 337 | 337 | 10 |
| **5.5** | West Albany Centre | 8 | 167 | 167 | 7 |
| **5.6** | Groot-Winterheokberge | 2 | 156 | 156 | 4 |
| **5.7** | Cockscomb | 1 | 60 | 60 | 3 |
| **5.8** | Oosterbaai | 1 | 64 | 64 | 1 |
| **5.9** | East London | 1 | 15 | 15 | 1 |
| **5.10** | Kiwane | 1 | 7 | 7 | 1 |
| **5.11** | Remainder (includes 3 cells: CoE to BR) | 9 | 219 | 2 | 0 |
| **6** | Agulhas Plains | 11 | 758 | 224 | 101 |
| **6.1** | West Agulhas Plains | 7 | 624 | 624 | 55 |
| **6.2** | Potberg | 4 | 349 | 349 | 28 |
| **7** | Nieuwoudtville | 6 | 275 | 103 | 74 |
| **7.1** | Nieuwoudtville Core | 4 | 264 | 98 | 69 |
| **7.2** | Rooiberg | 1 | 12 | 4 | 2 |
| **7.3** | Central Tankwa | 1 | 7 | 1 | 1 |
| **8** | Groot-Winterhoek - Skurweberg | 6 | 924 | 155 | 70 |
| **8.1** | Groot-Winterhoek - Skurweberg Core | 4 | 888 | 888 | 64 |
| **8.2** | Heuningberg | 1 | 78 | 3 | 1 |
| **8.3** | Kasteelberg | 1 | 86 | 1 | 1 |
| **9** | Boland Mountains | 4 | 1072 | 152 | 50 |
| **10** | Northern NWPC | 6 | 281 | 87 | 48 |
| **10.1** | Gifberg-Matsikammaberg | 4 | 256 | 256 | 41 |
| **10.2** | Boegoeberge | 2 | 61 | 61 | 6 |
| **11** | Central Cederberg | 7 | 596 | 119 | 44 |
| **12** | Piketberg/Olifantsberge and Northern Sandveld | 10 | 540 | 105 | 40 |
| **12.1** | Piketberg | 4 | 379 | 379 | 24 |
| **12.2** | North Sandveld | 4 | 324 | 324 | 9 |
| **12.3** | Remainder (includes 1 cell: CoE to BR) | 2 | 29 | 0 | 0 |
| **13** | Vanrhynsdorp Plains | 4 | 80 | 43 | 33 |
| **14** | Hexrivierberge | 3 | 661 | 65 | 25 |
| **15** | Saldanha Peninsula | 9 | 220 | 40 | 25 |
| **15.1** | Saldanha Core | 5 | 100 | 100 | 14 |
| **15.2** | Lambert's Bay | 1 | 42 | 8 | 3 |
| **15.3** | Hopefield | 1 | 110 | 4 | 1 |
| **15.4** | Aurora | 1 | 42 | 1 | 1 |
| **15.5** | Remainder | 1 | 37 | 0 | 0 |
| **16** | West Langeberg - Waboomsberg | 5 | 362 | 41 | 21 |
| **17** | Witteberg | 8 | 364 | 43 | 17 |
| **17.1** | Witteberg Core | 6 | 336 | 336 | 14 |
| **17.2** | Towerkop | 1 | 87 | 2 | 1 |
| **17.3** | Remainder | 1 | 4 | 1 | 0 |
| **18** | NE Escarpment | 22 | 138 | 23 | 17 |
| **18.1** | NE Escarpment Core | 7 | 68 | 68 | 6 |
| **18.2** | Barberton-Lebombo | 6 | 87 | 8 | 6 |
| **18.3** | Wolkeberg-Soutpansberg | 5 | 55 | 3 | 2 |
| **18.5** | Remainder | 4 | 42 | 1 | 0 |
| **19** | Kamiesberg | 14 | 99 | 23 | 17 |
| **19.1** | Kamiesberg Core | 7 | 89 | 18 | 13 |
| **19.2** | Garies | 2 | 6 | 1 | 1 |
| **19.3** | Remainder | 7 | 40 | 6 | 0 |
| **20** | Eastern Drakensberg | 20 | 163 | 25 | 13 |
| **20.1** | Eastern Drakensberg Core | 4 | 106 | 6 | 3 |
| **20.2** | Southern Drakensberg | 5 | 109 | 4 | 3 |
| **20.3** | Natal Midlands | 4 | 45 | 1 | 1 |
| **20.4** | Remainder | 7 | 70 | 1 | 0 |
| **21** | Lainsberg | 3 | 99 | 21 | 13 |
| **22** | Northern Southeastern Centre | 6 | 80 | 18 | 13 |
| **22.1** | Wolwefontein | 4 | 58 | 58 | 9 |
| **22.2** | Sunday's River Valley | 2 | 31 | 31 | 3 |
| **23** | Kouebokkeveld | 3 | 532 | 46 | 12 |
| **24** | East Riversdale Plains | 6 | 263 | 37 | 12 |
| **25** | Skurweberg - Swartrugberg | 3 | 333 | 28 | 9 |
| **26** | Natal Coastal Centre | 23 | 143 | 12 | 8 |
| **26.1** | Natal Coastal Centre | 21 | 140 | 140 | 7 |
| **26.2** | Ngunduza | 2 | 9 | 9 | 1 |
| **27** | Strandfontein | 3 | 34 | 14 | 7 |
| **28** | West Outeniekwaberg | 2 | 135 | 8 | 4 |
| **29** | Amathole Mountains | 10 | 147 | 6 | 3 |
| **29.1** | Amathole Mountains Core | 3 | 97 | 97 | 1 |
| **29.2** | Western Amathole | 2 | 44 | 1 | 1 |
| **29.3** | Remainder | 5 | 74 | 1 | 0 |
| **30** | Southern Drakensberg Centre | 10 | 102 | 5 | 3 |
| **30.1** | Southern Drakensberg | 3 | 70 | 70 | 2 |
| **30.2** | Remainder | 7 | 60 | 2 | 0 |
| **31** | Great Namaqualand Coastal Centre | 10 | 22 | 3 | 3 |
| **31.1** | Sonnikwa Namaqualand | 2 | 13 | 13 | 1 |
| **31.2** | Rosh Pina | 5 | 7 | 7 | 1 |
| **31.3** | Remainder | 3 | 8 | 1 | 0 |
| **32** | Karookop | 2 | 8 | 3 | 3 |
| **33** | Swartruggens | 2 | 87 | 6 | 2 |
| **34** | Southern Great Karoo | 3 | 46 | 5 | 2 |
| **35** | Western Drakensberg | 7 | 82 | 4 | 2 |
| **36** | Kokstad | 4 | 77 | 4 | 2 |
| **37** | Southeast Great Karoo | 2 | 36 | 2 | 2 |
| **38** | Northern Eastern Cape Escarpment | 6 | 22 | 2 | 1 |
| **39** | Garies-Nuwerus | 3 | 21 | 2 | 1 |
| **40** | Northern NE Escarpment | 2 | 20 | 2 | 1 |
| **41** | Central Tankwa | 1 | 8 | 2 | 2 |
| **42** | Natal North Coast | 3 | 8 | 2 | 2 |
| **43** | Transvaal Highveld | 4 | 31 | 1 | 1 |
| **44** | Kwaceza | 2 | 27 | 1 | 1 |
| **45** | Western Drakensberg2 | 7 | 27 | 1 | 1 |
| **46** | Hantamsberg | 1 | 21 | 1 | 1 |
| **47** | East Grootrivierberg | 1 | 15 | 1 | 1 |
| **48** | East Soutpansberg | 1 | 12 | 1 | 1 |
| **49** | North Barberton | 1 | 11 | 1 | 1 |
| **50** | Adelaide | 2 | 11 | 1 | 1 |
| **51** | Bhakaneni | 1 | 8 | 1 | 1 |
| **52** | NE Wolkeberg | 1 | 7 | 1 | 1 |
| **53** | McDougall's Bay | 1 | 6 | 1 | 1 |
| **54** | Swartkop | 1 | 6 | 1 | 1 |
| **55** | Landplaas | 1 | 5 | 1 | 1 |
| **56** | Sekameng | 1 | 5 | 1 | 1 |
| **57** | Tongaat | 1 | 5 | 1 | 1 |
| **58** | Stormsberg | 2 | 5 | 1 | 1 |
| **59** | Kubiskouberge-Langeberg | 2 | 4 | 1 | 1 |
| **60** | Brandvlei | 1 | 3 | 1 | 1 |
| **61** | Windhoek | 1 | 3 | 1 | 1 |
| **62** | Koingnaas | 1 | 2 | 1 | 1 |
| **63** | Lebombo | 1 | 2 | 1 | 1 |
| **64** | Joubertsberge | 1 | 1 | 1 | 1 |
| **65** | Mbazwana | 1 | 1 | 1 | 1 |
| **66** | SE Barberton | 1 | 1 | 1 | 1 |
| **67** | Outside | 653 | 434 | 73 | 9 |
