# Supplementary material for: Optimising Regionalisation Techniques: Identifying Centres of Endemism in the Extraordinarily Endemic-Rich Cape Floristic Region
Source: PLoS One. 2015 Jul 6;10(7):e0132538. doi: 10.1371/journal.pone.0132538 (PMC4493007; doi:10.1371/journal.pone.0132538)
Supplement: S1 Text — (DOCX) [file pone.0132538.s008.docx]

**S1 Text. The list of taxa endemic to the CoE and Sub-CoE identified.**

1. Southern SWPC Mountains
*Acmadenia candida (1), Acmadenia faucitincta (1), Acmadenia nivea (2), Adenandra brachyphylla (6), Adenandra gracilis (2), Adenandra multiflora (7), Agathosma hirsuta (1), Agathosma leptospermoides (3), Agathosma parva (2), Agathosma parvipetala (1), Agathosma rosmarinifolia (2), Agathosma stokoei (4), Alciope sp 1 (1), Anaxeton brevipes (3), Anaxeton hirsutum (2), Anaxeton lundgrenii (1), Arctotis dregei (1), Arctotis schlechteri (1), Aristea recisa (2), Askidiosperma rugosum (2), Aspalathus chenopoda gracilis (4), Aspalathus concavifolia (2), Aspalathus ferox (1), Aspalathus globosa (4), Aspalathus intervallaris (4), Aspalathus ramulosa (6), Aspalathus stokoei (3), Aspalathus subulata (2), Aspalathus taylorii (1), Aspalathus vacciniifolia (2), Athanasia imbricata (1), Athanasia scarbra (1), Babiana foliosa (1), Berzelia dregeana (5), Brachysiphon rupestris (2), Brunia albiflora (6), Calopsis clandestina (2), Calopsis sparsa (2), Chondropetalum decipiens (2), Cliffortia geniculata (1), Cliffortia graminea var elegens (1), Cliffortia heterophylla (3), Corymbium laxum ssp. bolusii (1), Cotula paradoxa (1), Crassula multiflora ssp leucantha (1), Cullumia selago (1), Cullumia setosa var. microcephala (2), Diastella fraterna (4), Diastella thymelaeoides subsp. meridiana (2), Diastella thymelaeoides subsp. thymelaeoides (2), Dimorphotheca walliana (1), Diosma pilosa (1), Diosma thyrsophora (1), Disa brevipetala (1), Disa pillansii (3), Elegia atratiflora (3), Elegia fucata (2), Endonema retzioides (3), Erepsia oxysepala (4), Erepsia steylerae (2), Erepsia villiersii (1), Erica accomodata var. ebracteata (1), Erica ampullaceae var. oblata (2), Erica atricha (2), Erica barbata (3), Erica bibax (4), Erica boucheri (2), Erica bruniifolia var. stellata (1), Erica cincta (1), Erica coccinea var. inflata (1), Erica colorans var. breviflora (1), Erica colorans var. hispidula (1), Erica comptonii (6), Erica corydalis (8), Erica crassifolia (1), Erica crateriformis (1), Erica curvifolia var. zeyheri (1), Erica cygnea (1), Erica daphniflora var. pedicellata (4), Erica diotiflora (2), Erica dulcis (2), Erica ecklonii (3), Erica eglandulosa (2), Erica embrothiifolia var. longiflora (2), Erica extrusa (4), Erica fascicularis var. imperialis (4), Erica fastigata var. immaculata (1), Erica fastigiata var. conventryana (2), Erica fervida (4), Erica foliacea (8), Erica foliaceae var. fulgens (1), Erica galgebergensis (1), Erica glabripes (1), Erica globiceps subsp. gracilis (5), Erica haemastoma (2), Erica hameriana (1), Erica hendricksei (1), Erica hendricksei var. hendricksei (1), Erica holosericea var. parviflora (4), Erica intonsa (2), Erica involvens (2), Erica jacksoniana (2), Erica jasminiflora (2), Erica jonasiana (1), Erica karwyderi (2), Erica kraussiana (1), Erica laevigata var. decurrens (1), Erica lanuginosa (4), Erica leucotrachela (4), Erica longifolia var. amplica (1), Erica lowryensis (2), Erica lowryensis var. lowryensis (2), Erica macroloma (3), Erica massonii (9), Erica nana (5), Erica octonaria (1), Erica oliveri (3), Erica pageana (5), Erica pannosa (3), Erica parviflora var. exigua (2), Erica parviflora var. hispida (1), Erica paucifolia subsp. ciliata (3), Erica paucifolia subsp. paucifolia (4), Erica paucifolia subsp. squarrosa (2), Erica permutata (3), Erica perplexa (1), Erica pillansii (6), Erica pillarkopensis (1), Erica pogonanthera (6), Erica porteri (1), Erica pulchelliflora (1), Erica pustulata (1), Erica pyrantha (1), Erica remota (2), Erica retorta (8), Erica rhodopis (3), Erica rufescens (4), Erica serratifolia (4), Erica sessiliflora var. oblanceolata (1), Erica stokoeanthus (1), Erica stokoei (5), Erica suffulta (6), Erica tenax (6), Erica trichophylla (2), Erica truncata (4), Erica tubercularis (4), Erica turrisbabylonica (1), Erica ustulescens (6), Erica vallis-aranearum (3), Erica vallis-gratiae (2), Erica vinacea (2), Erica viscaria (var. hispida) (1), Erica williamsiorum (2), Erica xeranthemifolia (3), Euchaetis glabra (3), Euryops indecorus (1), Euryops lasiocladus (1), Euryops tenuilobus (3), Felicia nigrescens (1), Galaxia barnardii (4), Geissorhiza lithicola (1), Gibbaeum esterhuyseniae (2), Gladiolus acuminatus (4), Grammitis poeppigiana (1), Gymnostephium angustifolium (1), Gymnostephium ciliare (3), Gymnostephium corymbosum (1), Gymnostephium hirsutum (1), Haemanthus canaliculatus (2), Helichrysum marifolium (3), Helichrysum rotundatum (3), Heliophila tricuspidata (2), Hesperantha juncifolia (1), Hippia sp 1 (1), Homeria comptonii (5), Homeria elegans (5), Indigofera superba (1), Ischyrolepis feminea (1), Ischyrolepis festuciformis (2), Ixia collina (1), Lachenalia moniliformis (1), Lachnaea laxa (2), Leucadendron elimense subsp. vyeboomense (2), Leucadendron globosum (2), Leucospermum bolusii (3), Leucospermum cordatum (2), Leucospermum harpagonatum (1), Lobelia laurentioides (1), Lobostemon capitatus (1), Lobostemon hottentoticus (1), Lotononis involucrata sub (1), Metalasia alfredii (1), Metalasia confusa (4), Metalasia cymbifolia (1), Metalasia humilis (1), Metalasia lichtensteinii (4), Metalasia oligocephala (1), Metalasia quinqueflora (4), Metalasia riparia (3), Metalasia seriphiifolia (1), Metalasia tenius (5), Metalasia tenuifolia (3), Mimetes arboreus (2), Mimetes capitulatus (6), Mimetes hottentoticus (4), Mimetes integrus (2), Mimetes palustris (2), Mimetes stokoei (1), Monadenia macrostachya (1), Moraea atropunctata (1), Moraea insolens (2), Muraltia aspalathoides (4), Muraltia caledonensis (4), Muraltia capensis (1), Muraltia chamaepitys (1), Muraltia concava (2), Muraltia guthriei (1), Muraltia hirsuta (3), Muraltia minuta (2), Muraltia mutabilis (1), Muraltia occidentalis (2), Muraltia paludosa (3), Muraltia schlechteri (3), Muraltia stokoei (2), Nebelia laevis (4), Nevillea singularis (1), Nivenia concinna (1), Nivenia dispar (1), Nivenia stokoei (6), Ornithogalum esterhuyseniae (1), Orothamnus zeyheri (5), Osmitopsis glabra (3), Osmitopsis parvifolia (5), Osteospermum rotundifolium (3), Paranomus bolusii (9), Paranomus sp. (2), Passerina burchellii (1), Pelargonium caledonicum (1), Pentameris longiglumis ssp gymnocolea (1), Phylica anomala (1), Phylica apiculata (2), Phylica burchellii (1), Phylica diosmoides (1), Phylica laevis (2), Phylica linifolia (1), Phylica lucens (1), Platycaulos cascadensis (4), Polyarrhena reflexa ssp brachyphylla/reflexa (1), Polyarrhena stricta (3), Prismatocarpus cordifolius (1), Prismatocarpus lycioides (1), Pseudobaeckea stokoei (2), Raspalia globosa (2), Raspalia phylicoides (2), Restio exilis (1), Restio fusiformis (4), Restio ingens (3), Restio involutus (2), Restio nuwebergensis (1), Restio pumilis (5), Restio scaber (1), Restio verrucosus (1), Serruria altiscapa (2), Serruria deluvialis (4), Serruria flagellifolia (5), Serruria heterophylla (6), Serruria meisneriana (3), Serruria rebeloi (1), Serruria stellata (2), Serruria viridifolia (2), Serruria williamsii (6), Sonderothamnus petraeus (4), Sonderothamnus speciosus (3), Sorocephalus crassifolius (2), Sorocephalus palustris (3), Sorocephalus pinifolius (3), Sorocephalus tenuifolius (4), Sparaxis fragrans (3), Sparaxis maculosa (1), Spatalla mollis (5), Spatalla prolifera (5), Spatalla racemosa (6), Staavia brownii (2), Staavia zeyheri (2), Stoebe humilis (3), Stoebe salteri (1), Stylapterus barbatus (1), Stylapterus micranthus (2), Thaminophyllum mundii (4), Thesium bathyschistum (2), Tritoniopsis flexuosa (1), Ursinia coronopifolia (1), Wahlenbergia effusa (1), Watsonia rogersii (6), Zyrphelis ciliaris ssp angustifolia (1), Zyrphelis ciliaris ssp hirsuta (1), Zyrphelis corymbosa (3), Zyrphelis glandulosa (1), Zyrphelis macrocarpa (1), Zyrphelis nervosa (3), Zyrphelis spathulata (6)*1.1. Hottentots-Holland - Kleinrivierberg
*Acmadenia candida (1), Acmadenia nivea (2), Adenandra brachyphylla (6), Agathosma hirsuta (1), Agathosma parvipetala (1), Agathosma rosmarinifolia (2), Agathosma stokoei (4), Alciope sp 1 (1), Anaxeton lundgrenii (1), Arctotis schlechteri (1), Askidiosperma rugosum (2), Aspalathus globosa (4), Aspalathus intervallaris (4), Aspalathus stokoei (3), Brachysiphon rupestris (2), Brunia albiflora (6), Calopsis clandestina (2), Calopsis sparsa (2), Chondropetalum decipiens (2), Cliffortia geniculata (1), Cliffortia graminea var elegens (1), Cliffortia heterophylla (3), Corymbium laxum ssp. bolusii (1), Cotula paradoxa (1), Crassula multiflora ssp leucantha (1), Diastella fraterna (4), Diastella thymelaeoides subsp. meridiana (2), Diastella thymelaeoides subsp. thymelaeoides (2), Dimorphotheca walliana (1), Disa brevipetala (1), Disa pillansii (3), Elegia atratiflora (3), Erepsia steylerae (2), Erica accomodata var. ebracteata (1), Erica atricha (2), Erica bruniifolia var. stellata (1), Erica cincta (1), Erica coccinea var. inflata (1), Erica colorans var. breviflora (1), Erica colorans var. hispidula (1), Erica comptonii (6), Erica crassifolia (1), Erica crateriformis (1), Erica curvifolia var. zeyheri (1), Erica cygnea (1), Erica dulcis (2), Erica ecklonii (3), Erica extrusa (4), Erica fastigata var. immaculata (1), Erica fastigiata var. conventryana (2), Erica fervida (4), Erica foliaceae var. fulgens (1), Erica glabripes (1), Erica haemastoma (2), Erica hendricksei (1), Erica hendricksei var. hendricksei (1), Erica holosericea var. parviflora (4), Erica intonsa (2), Erica jacksoniana (2), Erica karwyderi (2), Erica lanuginosa (4), Erica leucotrachela (4), Erica lowryensis (2), Erica lowryensis var. lowryensis (2), Erica macroloma (3), Erica nana (5), Erica octonaria (1), Erica oliveri (3), Erica parviflora var. hispida (1), Erica paucifolia subsp. ciliata (3), Erica paucifolia subsp. paucifolia (4), Erica perplexa (1), Erica porteri (1), Erica pulchelliflora (1), Erica pustulata (1), Erica rhodopis (3), Erica sessiliflora var. oblanceolata (1), Erica stokoeanthus (1), Erica stokoei (5), Erica tenax (6), Erica truncata (4), Erica tubercularis (4), Erica turrisbabylonica (1), Erica vallis-aranearum (3), Erica vinacea (2), Erica viscaria (var. hispida) (1), Erica williamsiorum (2), Erica xeranthemifolia (3), Euchaetis glabra (3), Euryops indecorus (1), Euryops lasiocladus (1), Geissorhiza lithicola (1), Grammitis poeppigiana (1), Haemanthus canaliculatus (2), Hesperantha juncifolia (1), Indigofera superba (1), Ischyrolepis feminea (1), Ischyrolepis festuciformis (2), Leucadendron elimense subsp. vyeboomense (2), Leucadendron globosum (2), Leucospermum bolusii (3), Leucospermum cordatum (2), Lobelia laurentioides (1), Lobostemon capitatus (1), Lobostemon hottentoticus (1), Metalasia confusa (4), Metalasia cymbifolia (1), Metalasia humilis (1), Metalasia lichtensteinii (4), Metalasia quinqueflora (4), Metalasia riparia (3), Metalasia seriphiifolia (1), Mimetes arboreus (2), Mimetes capitulatus (6), Mimetes hottentoticus (4), Mimetes integrus (2), Mimetes stokoei (1), Monadenia macrostachya (1), Muraltia capensis (1), Muraltia chamaepitys (1), Muraltia guthriei (1), Muraltia minuta (2), Muraltia mutabilis (1), Muraltia paludosa (3), Muraltia stokoei (2), Nivenia concinna (1), Ornithogalum esterhuyseniae (1), Osmitopsis glabra (3), Osteospermum rotundifolium (3), Paranomus sp. (2), Pentameris longiglumis ssp gymnocolea (1), Phylica diosmoides (1), Phylica linifolia (1), Platycaulos cascadensis (4), Polyarrhena reflexa ssp brachyphylla/reflexa (1), Polyarrhena stricta (3), Prismatocarpus cordifolius (1), Pseudobaeckea stokoei (2), Raspalia globosa (2), Restio fusiformis (4), Restio involutus (2), Restio nuwebergensis (1), Restio verrucosus (1), Serruria deluvialis (4), Serruria flagellifolia (5), Serruria meisneriana (3), Serruria rebeloi (1), Sonderothamnus petraeus (4), Sonderothamnus speciosus (3), Sorocephalus palustris (3), Sorocephalus tenuifolius (4), Staavia brownii (2), Stoebe humilis (3), Stoebe salteri (1), Stylapterus barbatus (1), Stylapterus micranthus (2), Thesium bathyschistum (2), Tritoniopsis flexuosa (1), Ursinia coronopifolia (1), Zyrphelis corymbosa (3)*1.2. Riviersonderendberg
*Acmadenia faucitincta (1), Adenandra gracilis (2), Agathosma leptospermoides (3), Agathosma parva (2), Anaxeton brevipes (3), Anaxeton hirsutum (2), Arctotis dregei (1), Aspalathus concavifolia (2), Aspalathus ferox (1), Aspalathus taylorii (1), Athanasia imbricata (1), Athanasia scarbra (1), Babiana foliosa (1), Cullumia selago (1), Diosma pilosa (1), Diosma thyrsophora (1), Elegia fucata (2), Endonema retzioides (3), Erepsia villiersii (1), Erica ampullaceae var. oblata (2), Erica boucheri (2), Erica diotiflora (2), Erica eglandulosa (2), Erica embrothiifolia var. longiflora (2), Erica galgebergensis (1), Erica globiceps subsp. gracilis (5), Erica hameriana (1), Erica jonasiana (1), Erica kraussiana (1), Erica laevigata var. decurrens (1), Erica longifolia var. amplica (1), Erica paucifolia subsp. squarrosa (2), Erica permutata (3), Erica pillarkopensis (1), Erica pyrantha (1), Erica rufescens (4), Erica trichophylla (2), Erica vallis-gratiae (2), Felicia nigrescens (1), Gibbaeum esterhuyseniae (2), Gymnostephium angustifolium (1), Gymnostephium corymbosum (1), Gymnostephium hirsutum (1), Helichrysum rotundatum (3), Heliophila tricuspidata (2), Hippia sp 1 (1), Ixia collina (1), Lachenalia moniliformis (1), Leucospermum harpagonatum (1), Lotononis involucrata sub (1), Metalasia alfredii (1), Metalasia oligocephala (1), Metalasia tenius (5), Moraea atropunctata (1), Moraea insolens (2), Nebelia laevis (4), Nevillea singularis (1), Nivenia dispar (1), Passerina burchellii (1), Pelargonium caledonicum (1), Phylica anomala (1), Phylica apiculata (2), Phylica burchellii (1), Phylica lucens (1), Prismatocarpus lycioides (1), Restio exilis (1), Restio ingens (3), Restio scaber (1), Serruria stellata (2), Serruria viridifolia (2), Sorocephalus crassifolius (2), Sorocephalus pinifolius (3), Sparaxis maculosa (1), Staavia zeyheri (2), Wahlenbergia effusa (1), Zyrphelis ciliaris ssp angustifolia (1), Zyrphelis ciliaris ssp hirsuta (1), Zyrphelis glandulosa (1), Zyrphelis macrocarpa (1)*

2. Cape Peninsula and Southern Sandveld
*Anaxeton arborescenes (2), Anthochortus capensis (2), Aspalathus barbata (2), Aspalathus borboniifolia (1), Aspalathus glabrata (1), Aspalathus horizontalis (3), Aspalathus lotiflora (1), Aspalathus rycroftii (1), Aspalathus variegata (3), Babiana leipoldtii (2), Bobartia gladiata subsp. major (1), Brachystelma occidentale (1), Calopsis gracilis (2), Cenia duckittiae (2), Chasmanthe floribunda var. duckittii (1), Cliffortia discolor (1), Cliffortia ericifolia (5), Cotula myriophylloides (2), Cynanchum zeyheri (1), Diascia heterandra (1), Diastella divaricata subsp. divaricata (2), Disa tenella ssp tenella (2), Drimia minor (2), Elegia intermedia (1), Elegia tenuis? (2), Erepsia forficata (2), Erepsia hallii (1), Erepsia patula (2), Erica acockii (1), Erica alexandri subsp. acockii (1), Erica amoena (3), Erica annectens (1), Erica blancheana (2), Erica clavisepala (2), Erica crucistigmatica (3), Erica cyrilliflora (1), Erica decora (2), Erica diosmifolia (2), Erica eburnea (2), Erica empetrina (2), Erica fairii (2), Erica gilva (3), Erica haematocodon (2), Erica halicaba (2), Erica halicacaba (2), Erica heleogena (2), Erica limosa (1), Erica malmesburiensis (2), Erica nevillei (3), Erica oxycoccifolia (2), Erica paludicola (1), Erica physodes (3), Erica pyxidiflora (2), Erica quadrisulcata (3), Erica salteri (1), Erica sociorum (1), Erica subcapitata (2), Erica urna-viridis (2), Erica viscaria var. decora (2), Erica woodii (1), Galenia fruticosa var. prostrata (1), Gastrodia sesamoides (2), Geissorhiza darlingensis (1), Geissorhiza eurystigma (3), Geissorhiza humilis (3), Gerbera wrightii (3), Gladiolus aureus (1), Gladiolus bonaespei (3), Gladiolus jonquilliodorus (2), Gladiolus monticola (2), Gladiolus quadrangulus (5), Helichrysum fruticans (2), Helichrysum grandiflorum (2), Heliophila cinerea (2), Heliophila tabularis (2), Hermannia micrantha (2), Hermannia procumbens ssp procumbens (3), Herschelia barbata (2), Herschelianthe barbata (3), Ixia maculata var. maculata (2), Ixia tenuifolia (4), Lachenalia purpureocaerulea (3), Leucadendron macowanii (3), Leucadendron strobilinum (2), Leucospermum conocarpodendron subsp. conocarpodendron (1), Macrostylis cassiopoides ssp dregeana (4), Marasmodes dummeri (2), Metalasia compacta (3), Metalasia distans (2), Metalasia divergens ssp. fusca (1), Metalasia pulchella (4), Mimetes fimbriifolius (3), Monadenia ecalcarata (1), Moraea amissa (1), Moraea aristata (1), Moraea villosa subsp. elandsmontana (1), Muraltia acipetala (2), Muraltia comptonii (1), Muraltia curvipetala (1), Muraltia demissa (2), Muraltia diabolica (2), Muraltia mixta (2), Muraltia orbicularis (1), Osmitopsis denata (2), Oxalis fragilis var. fragilis (1), Oxalis levis (1), Oxalis perineson (1), Pentameris longiglumis ssp longiglumis (1), Phylica schlechteri (1), Psoralea glaucina (3), Restio acockii (3), Restio communis (1), Romulea eximia (4), Romulea papyracea (1), Ruschia filamentosa (2), Saphesia flaccida (2), Scopelogena verruculata (2), Senecio crispus (Form 1) (1), Senecio verbascifolius (1), Serruria collina (2), Serruria decumbens (2), Serruria foeniculacea (4), Serruria vallaris (3), Serruria villosa (2), Staavia dodii (2), Steirodiscus speciosus (2), Stoebe gomphrenoides (4), Stoebe rosea (1), Tetraria brachyphylla (1), Tetraria compacta (1), Thamnochortus levynsiae (2), Thamnochortus nutans (2), Thesium litoreum (1), Wahlenbergia clavatula (1), Wahlenbergia dunantii (1), Wahlenbergia rotundifolia (1), Wahlenbergia saxifragoides (1)*2.1. Cape Peninsula
*Anaxeton arborescenes (2), Anthochortus capensis (2), Aspalathus barbata (2), Aspalathus borboniifolia (1), Aspalathus lotiflora (1), Aspalathus variegata (3), Bobartia gladiata subsp. major (1), Brachystelma occidentale (1), Calopsis gracilis (2), Cliffortia discolor (1), Cotula myriophylloides (2), Cynanchum zeyheri (1), Diascia heterandra (1), Diastella divaricata subsp. divaricata (2), Drimia minor (2), Elegia intermedia (1), Elegia tenuis? (2), Erepsia forficata (2), Erepsia patula (2), Erica acockii (1), Erica alexandri subsp. acockii (1), Erica amoena (3), Erica annectens (1), Erica blancheana (2), Erica clavisepala (2), Erica cyrilliflora (1), Erica diosmifolia (2), Erica eburnea (2), Erica empetrina (2), Erica fairii (2), Erica gilva (3), Erica haematocodon (2), Erica halicaba (2), Erica halicacaba (2), Erica heleogena (2), Erica limosa (1), Erica nevillei (3), Erica oxycoccifolia (2), Erica paludicola (1), Erica physodes (3), Erica pyxidiflora (2), Erica quadrisulcata (3), Erica salteri (1), Erica sociorum (1), Erica subcapitata (2), Erica urna-viridis (2), Erica viscaria var. decora (2), Gastrodia sesamoides (2), Geissorhiza humilis (3), Gerbera wrightii (3), Gladiolus aureus (1), Gladiolus bonaespei (3), Gladiolus jonquilliodorus (2), Gladiolus monticola (2), Helichrysum fruticans (2), Helichrysum grandiflorum (2), Heliophila cinerea (2), Heliophila tabularis (2), Hermannia micrantha (2), Leucadendron macowanii (3), Leucadendron strobilinum (2), Leucospermum conocarpodendron subsp. conocarpodendron (1), Metalasia compacta (3), Metalasia divergens ssp. fusca (1), Metalasia pulchella (4), Mimetes fimbriifolius (3), Monadenia ecalcarata (1), Moraea aristata (1), Moraea villosa subsp. elandsmontana (1), Muraltia acipetala (2), Muraltia comptonii (1), Muraltia curvipetala (1), Muraltia demissa (2), Muraltia diabolica (2), Muraltia mixta (2), Muraltia orbicularis (1), Osmitopsis denata (2), Pentameris longiglumis ssp longiglumis (1), Phylica schlechteri (1), Psoralea glaucina (3), Restio communis (1), Romulea papyracea (1), Ruschia filamentosa (2), Scopelogena verruculata (2), Senecio crispus (Form 1) (1), Senecio verbascifolius (1), Serruria collina (2), Serruria decumbens (2), Serruria villosa (2), Staavia dodii (2), Stoebe rosea (1), Tetraria brachyphylla (1), Tetraria compacta (1), Thamnochortus levynsiae (2), Thamnochortus nutans (2), Wahlenbergia clavatula (1), Wahlenbergia dunantii (1), Wahlenbergia rotundifolia (1), Wahlenbergia saxifragoides (1)*2.2. Southern Sandveld
*Aspalathus glabrata (1), Aspalathus horizontalis (3), Aspalathus rycroftii (1), Babiana leipoldtii (2), Cenia duckittiae (2), Chasmanthe floribunda var. duckittii (1), Erepsia hallii (1), Erica malmesburiensis (2), Erica woodii (1), Galenia fruticosa var. prostrata (1), Geissorhiza darlingensis (1), Geissorhiza eurystigma (3), Ixia tenuifolia (4), Lachenalia purpureocaerulea (3), Moraea amissa (1), Oxalis fragilis var. fragilis (1), Oxalis levis (1), Oxalis perineson (1), Romulea eximia (4), Saphesia flaccida (2), Steirodiscus speciosus (2), Thesium litoreum (1)*

3. Langeberg Centre
*Acmadenia burchellii (2), Acmadenia latifolia (1), Acmadenia laxa (2), Acmadenia nivenii (1), Acmadenia trigona (2), Adenandra fragrans (5), Adromischus humilis (1), Agathosma gnidiiflora (1), Agathosma ionii (1), Agathosma linifolia (3), Agathosma serratifolia (4), Agathosma umbonata (2), Anderbergia fallax (1), Anisodontea pseudocapensis (2), Arctotis incisa (1), Argyrolobium muirii (1), Aspalathus acanthes (3), Aspalathus cordicarpa (1), Aspalathus hypnoides (2), Aspalathus inops (4), Aspalathus longifolia (2), Aspalathus vulpina (5), Aspalathus willdenowiana (eastern form (3), Aster bowiei (2), Athanasia inopinata (1), Athanasia inopnata (1), Berzelia burchellii (3), Berzelia galpinii (3), Bobartia parva (4), Calopsis monostylis (3), Chrysocoma flava (1), Cliffortia alata (3), Cliffortia densa (4), Cliffortia lanceolata (6), Coleonema pulchrum (1), Coleonema virgatum (4), Cyrtanthus herrei (1), Diascia dielsiana (1), Dicoma fruticosa (1), Diosma fallax (1), Diosma strumosa (1), Disa subtenuicornis (1), Disa tripetaloides ssp aurata (3), Empleurum fragrans (1), Erepsia dubia (1), Erepsia polita (1), Erica blenna var. grandiflora (4), Erica burchelliana (1), Erica chlorosepala (6), Erica dysantha (2), Erica granulatifolia (1), Erica heterophylla (1), Erica lageniformis (1), Erica langebergensis (1), Erica macrophylla (5), Erica miniscula (2), Erica minutissima (1), Erica obconica (1), Erica oophylla (2), Erica oxyndra (2), Erica podophylla (3), Erica pubigera (5), Erica rhodantha (2), Erica tradouwensis (6), Erica vallis-fluminis (3), Erica winteri (1), Euchaetis avisylvana (2), Euchaetis longicornus (1), Ficinia quinquangularis (1), Freesia sparrmannii (2), Gibbaeum angulipes (2), Gibbaeum schwantesii (1), Gladiolus bilineatus (3), Gladiolus engysiphon (3), Gymnostephium fruiticosum (1), Haworthia serrata (1), Herschelia hians (1), Herschelia schlechteriana (1), Hippia hutchinsonii (2), Hippia integrifolia (4), Holothrix lindleyana (1), Hypodiscus montanus (1), Ischyrolepis affinis (1), Ischyrolepis leeurivierberg? (1), Ixia gloriosa (1), Langebergia canescens (1), Laurentia longitubus (1), Leucadendron radiatum (4), Leucadendron tradouwense (4), Leucospermum mundii (7), Leucospermum saxatile (3), Leucospermum winteri (3), Lobelia dasyphylla (1), Lobelia hypsibata (1), Lobostemon muirii (2), Lotononis lamprifolia (1), Mairia petiolata (1), Metalasia galpinii (1), Mniothamnea callunoides (4), Muiria hortenseae (2), Muraltia langebergensis (2), Nivenia fruticosa (3), Oedera muirii (1), Osteospermum imbricatum var microcephalum (1), Osteospermum pyrifolium (1), Osteospermum reticulatum ssp/var dregei? (1), Oxalis anomala (2), Pachites appressa (3), Pentameris sp nov (1), Petalacte canescens? (3), Phylica longimontana (1), Phylica recurvifolia (1), Platycaulos acutus (1), Polhillia connata (2), Polygala langebergensis (1), Pteronia beckioides (1), Pteronia hirsuta var cephalotes (1), Raspalia schlechteri (3), Restio arcuatus (2), Restio fragilis (1), Restio implicatus (1), Restio peculiaris (1), Restio perseverans (2), Rhodocoma foliosus (1), Senecio muirii (1), Spatalla nubicola (1), Stylapterus dubius (2), Stylapterus ericifolius (1), Thamnochortus amoena (1), Thamnochortus ellipticus (1), Tylecodon albiflorus (1), Wahlenbergia oligantha (1), Zeuktophyllum suppositum (1), Zyrphelis fruiticosa (4), Zyrphelis gracilis (2)*3.1. Langeberg
*Acmadenia burchellii (2), Acmadenia latifolia (1), Acmadenia nivenii (1), Acmadenia trigona (2), Adenandra fragrans (5), Adromischus humilis (1), Agathosma gnidiiflora (1), Agathosma ionii (1), Agathosma serratifolia (4), Agathosma umbonata (2), Anderbergia fallax (1), Anisodontea pseudocapensis (2), Arctotis incisa (1), Argyrolobium muirii (1), Aspalathus acanthes (3), Aspalathus cordicarpa (1), Aspalathus longifolia (2), Aspalathus vulpina (5), Aspalathus willdenowiana (eastern form (3), Aster bowiei (2), Athanasia inopinata (1), Athanasia inopnata (1), Berzelia burchellii (3), Berzelia galpinii (3), Bobartia parva (4), Calopsis monostylis (3), Chrysocoma flava (1), Cliffortia alata (3), Cliffortia densa (4), Coleonema pulchrum (1), Coleonema virgatum (4), Cyrtanthus herrei (1), Diascia dielsiana (1), Dicoma fruticosa (1), Diosma strumosa (1), Disa subtenuicornis (1), Empleurum fragrans (1), Erepsia polita (1), Erica burchelliana (1), Erica dysantha (2), Erica granulatifolia (1), Erica heterophylla (1), Erica lageniformis (1), Erica langebergensis (1), Erica miniscula (2), Erica minutissima (1), Erica obconica (1), Erica podophylla (3), Erica rhodantha (2), Erica vallis-fluminis (3), Erica winteri (1), Euchaetis avisylvana (2), Euchaetis longicornus (1), Ficinia quinquangularis (1), Gibbaeum angulipes (2), Gibbaeum schwantesii (1), Gymnostephium fruiticosum (1), Herschelia hians (1), Herschelia schlechteriana (1), Hippia integrifolia (4), Hypodiscus montanus (1), Ischyrolepis affinis (1), Ischyrolepis leeurivierberg? (1), Ixia gloriosa (1), Langebergia canescens (1), Laurentia longitubus (1), Leucadendron radiatum (4), Leucadendron tradouwense (4), Leucospermum mundii (7), Leucospermum saxatile (3), Leucospermum winteri (3), Lobelia dasyphylla (1), Lobelia hypsibata (1), Lobostemon muirii (2), Lotononis lamprifolia (1), Mairia petiolata (1), Metalasia galpinii (1), Mniothamnea callunoides (4), Muiria hortenseae (2), Muraltia langebergensis (2), Nivenia fruticosa (3), Oedera muirii (1), Osteospermum imbricatum var microcephalum (1), Osteospermum pyrifolium (1), Osteospermum reticulatum ssp/var dregei? (1), Oxalis anomala (2), Pachites appressa (3), Pentameris sp nov (1), Petalacte canescens? (3), Phylica longimontana (1), Platycaulos acutus (1), Polhillia connata (2), Polygala langebergensis (1), Pteronia hirsuta var cephalotes (1), Raspalia schlechteri (3), Restio arcuatus (2), Restio fragilis (1), Restio implicatus (1), Restio peculiaris (1), Restio perseverans (2), Rhodocoma foliosus (1), Senecio muirii (1), Spatalla nubicola (1), Stylapterus dubius (2), Stylapterus ericifolius (1), Thamnochortus amoena (1), Thamnochortus ellipticus (1), Tylecodon albiflorus (1), Wahlenbergia oligantha (1), Zeuktophyllum suppositum (1), Zyrphelis fruiticosa (4), Zyrphelis gracilis (2)*3.2. West Riversdale Plains
*Acmadenia laxa (2), Diosma fallax (1), Erepsia dubia (1), Haworthia serrata (1), Holothrix lindleyana (1), Phylica recurvifolia (1), Pteronia beckioides (1)*

4. Karoo Mountain Centres
*Acmadenia fruticosa (3), Acmadenia sheilae (7), Agathosma ovalifolia (4), Agathosma purpurea (2), Agathosma zwartbergense (4), Anderbergia elsiae (1), Anderbergia epaleata (1), Anderbergia rooibergensis (1), Anisodontea theronii (1), Anisothrix integra (1), Aspalathus incana (2), Aspalathus lamarckiana (3), Aspalathus oliveri (2), Aspalathus patens (4), Aspalathus vermiculata (3), Astroloba herrei (3), Berkheya francisci (2), Bobartia paniculata (2), Calotesta alba (2), Ceropegia barbata (1), Chasmatophyllum willowmorense (1), Chrysocoma acicularis (1), Cliffortia aculeata (4), Cliffortia concinna (2), Cliffortia crassinervis (1), Cliffortia nivenioides (1), Cliffortia robusta (5), Cliffortia setifolia (3), Coelidium vlokii (1), Cullumia rigida (1), Cyclopia burtonii (2), Cyphia longilobata (1), Diascia ramosa (1), Dicoma relhanioides (2), Diosma rourkei (3), Elegia altigena (1), Erica andreaei (2), Erica angulosa (6), Erica atromontana (5), Erica blesbergensis (1), Erica costatisepala (2), Erica esterhuyseniae var. tetramera (1), Erica flocciflora (2), Erica gossypioides (4), Erica granulosa (3), Erica inamoena (2), Erica inordinata (3), Erica ionii (2), Erica kammanassieae (1), Erica montis-hominis (1), Erica pallens (1), Erica phaeocarpa (4), Erica saxigena (2), Erica singularis (2), Erica syngenesia (1), Erica toringbergensis (2), Erica umbonata (1), Erica wendlandiana (3), Erica zwartbergensis (1), Eriospermum aequilibre (2), Eriospermum bruynsii (1), Eriospermum crispum (1), Eriospermum rhizomatum (2), Euchaetis vallis-simiae (2), Euphorbia decepta (1), Euphorbia symmetrica (1), Euryops comptonii (2), Euryops glutinosus (2), Euryops integrifolius (2), Euryops zeyheri (1), Felicia esterhuyseniae (1), Gasteria rawlinsonii (2), Geissorhiza elsiae (1), Geissorhiza nigromontana (1), Gladiolus nigromontanus (1), Gladiolus robustus (1), Haworthia comptoniana (1), Haworthia graminifolia (1), Helichrysum fourcadei (1), Heliophila rimicola (1), Herschelia spathulata ssp tripartita (2), Hesperantha truncatula (1), Huernia thudichumii (2), Hypodiscus ramosus (2), Hypodiscus sp nov (1), Lachenalia haarlemensis (5), Leucadendron rourkei (8), Leucospermum secundifolium (3), Lotononis acocksii (1), Lotononis dahlgrenii (2), Metalasia tricolor (1), Muraltia carnosa (1), Muraltia elsieae (1), Ornithogalum sardienii (1), Osteospermum asperulum (1), Otholobium lucens (2), Otholobium rubicundum (3), Othonna osteopermoides (2), Oxalis dichotoma (2), Oxalis fourcadei (2), Paranomus centaureoides (5), Pentameris swartbergensis (1), Phylica nigromontana (2), Phylica sericea (1), Phyllopodium dolomiticum (1), Pleiospilos nelii (4), Polygala gracilipes (1), Protea aristata (4), Protea intonsa (14), Protea montana (8), Protea pruinosa (4), Protea subvestita (2), Psoralea swartbergensis (1), Pteronia stricta var longifolia (1), Relhania decussata (5), Restio papyraceus (3), Restio rarus (1), Rhadamanthus urantherus (1), Roggeveldia montana (1), Romulea vlokii (1), Staberoha stokoei (2), Syringodea derustensis (1), Syringodea saxatilis (1), Thamnochortus papyraceus (2), Thamnochortus sp nov (1), Trichodiadema aureum (1), Trichodiadema burgeri (1), Trichodiadema hallii (2), Watsonia emiliae (1), Widdringtonia schwarzii (2)*4.1. Klein Swartberg-Touwsberg
*Acmadenia fruticosa (3), Anderbergia epaleata (1), Anisothrix integra (1), Aspalathus incana (2), Aspalathus lamarckiana (3), Calotesta alba (2), Chrysocoma acicularis (1), Cliffortia crassinervis (1), Cullumia rigida (1), Cyphia longilobata (1), Erica costatisepala (2), Erica inamoena (2), Erica syngenesia (1), Erica toringbergensis (2), Erica umbonata (1), Eriospermum rhizomatum (2), Euryops comptonii (2), Euryops glutinosus (2), Heliophila rimicola (1), Hesperantha truncatula (1), Leucospermum secundifolium (3), Muraltia elsieae (1), Ornithogalum sardienii (1), Othonna osteopermoides (2), Pentameris swartbergensis (1), Phylica sericea (1), Protea aristata (4), Protea subvestita (2), Restio papyraceus (3), Restio rarus (1), Rhadamanthus urantherus (1), Syringodea saxatilis (1), Thamnochortus papyraceus (2), Trichodiadema hallii (2)*4.2. Groot Swartberg
*Aspalathus oliveri (2), Berkheya francisci (2), Cliffortia aculeata (4), Cliffortia nivenioides (1), Erica blesbergensis (1), Erica pallens (1), Erica singularis (2), Erica zwartbergensis (1), Geissorhiza nigromontana (1), Gladiolus nigromontanus (1), Haworthia graminifolia (1), Huernia thudichumii (2), Muraltia carnosa (1), Osteospermum asperulum (1), Otholobium lucens (2), Phylica nigromontana (2), Phyllopodium dolomiticum (1), Psoralea swartbergensis (1), Pteronia stricta var longifolia (1), Staberoha stokoei (2), Syringodea derustensis (1), Trichodiadema burgeri (1), Watsonia emiliae (1)*4.3. Rooiberg
*Anderbergia elsiae (1), Anderbergia rooibergensis (1), Anisodontea theronii (1), Ceropegia barbata (1), Cliffortia concinna (2), Diascia ramosa (1), Eriospermum bruynsii (1), Eriospermum crispum (1), Euryops zeyheri (1), Lotononis acocksii (1), Lotononis dahlgrenii (2), Metalasia tricolor (1), Polygala gracilipes (1)*4.4. Kammanassieberg
*Bobartia paniculata (2), Elegia altigena (1), Erica esterhuyseniae var. tetramera (1), Erica kammanassieae (1), Erica montis-hominis (1), Felicia esterhuyseniae (1), Geissorhiza elsiae (1), Hypodiscus sp nov (1), Romulea vlokii (1), Thamnochortus sp nov (1)*4.5. Kougaberg-West Baviaanskloof
*Coelidium vlokii (1), Erica flocciflora (2), Euphorbia symmetrica (1), Helichrysum fourcadei (1), Herschelia spathulata ssp tripartita (2), Hypodiscus ramosus (2), Widdringtonia schwarzii (2)*4.6. Slypsteenberg-Antoniesberg
*Chasmatophyllum willowmorense (1), Erica saxigena (2), Euphorbia decepta (1), Haworthia comptoniana (1), Pleiospilos nelii (4), Roggeveldia montana (1)*4.7. East Baviaansberg
*Diosma rourkei (3), Euchaetis vallis-simiae (2), Gasteria rawlinsonii (2), Gladiolus robustus (1)*4.8. North Baviaanskloof
*Trichodiadema aureum (1)*

5. Southeastern Centre
*Acmadenia alternifolia (5), Acmadenia gracilis (1), Acmadenia kiwanensis (1), Acmadenia maculata (2), Acmadenia rupicola (1), Adromischus mammillaris (1), Agathosma acutissima (3), Agathosma alaris (1), Agathosma citriodora (1), Agathosma clavisepala (4), Agathosma planifolia (5), Agathosma stenopetala (4), Anisodontea alexandri (1), Aspalathus argyrophanes (2), Aspalathus cerrantha (9), Aspalathus cliffortiifolia (2), Aspalathus digitifolia (2), Aspalathus glabrescens (2), Aspalathus hirta stellaris (1), Aspalathus intermedia (5), Aspalathus lanceicarpa (2), Aspalathus nivea (8), Aspalathus recurvispina (4), Aspalathus teres (11), Aspalathus teres thodei (2), Bobartia gracilis (1), Bobartia macrocarpa (4), Brachystelma comptum (2), Brachystelma schoenlandianum (1), Brunsvigia litoralis (1), Ceratandra grandiflora (18), Coleonema pulchellum (6), Cyclopia filiformis (1), Cyclopia longifolia (1), Cyrtanthus flammosus (1), Cyrtanthus spiralis (1), Cyrtanthus staadensis (1), Diascia pentheri (1), Disa arida (1), Elegia thyrsoidea (6), Encephalartos longifolius (1), Erica adaequata (1), Erica aneimena (3), Erica beatricis (1), Erica chloroloma (11), Erica cordata var. arachnoidea (3), Erica coronanthera (4), Erica curviflora var. splendens (1), Erica curviflora var. sulcata (1), Erica deflexa (6), Erica etheliae (1), Erica glandulosa var. bondiae (3), Erica humansdorpensis (3), Erica inflaticalyx (2), Erica jeppei (1), Erica keetii (2), Erica laevigata var. elongata (1), Erica lanata (10), Erica lehmannii (3), Erica onusta (3), Erica outeniquae (3), Erica scabriuscula (14), Erica varderi (1), Erica velatiflora (2), Erica zeyheriana (6), Erica zitzikammensis (2), Euchaetis cristagalli (2), Euphorbia meloformis (1), Euryops polythricoides (2), Euryops ursinoides (1), Faurea macnaughtonii (3), Felicia jourbertinae ssp joubertinae (1), Felicia tsitsikamae (1), Gasteria ellaphieae (1), Gasteria glomerata (1), Gladiolus alatus var. algoensis (1), Gladiolus sempervirens (3), Helichrysum outeniquense (1), Herschelia excelsa (3), Herschelia newdigatae (1), Herschelianthe newdigateae (1), Huernia longii (2), Lachnaea glomerata (2), Leucadendron elens (2), Leucadendron olens (2), Leucadendron orientale (5), Leucospermum glabrum (8), Leucospermum hamatum (1), Lobelia dichroma (1), Lobelia montaguensis (1), Lobelia zwartkopensis (1), Manulea derustiana (1), Merxmuellera papposa (4), Mimetes chrysanthus (2), Muraltia lancifolia (2), Muraltia macroseras (4), Oldenburgia grandis (6), Orthopterum coegana (1), Osteospermum pterigoideum (4), Othonna membranifolia (3), Oxalis stellata var. gracilior (1), Oxylaena acicularis (5), Paranomus spathulatus type gamka (1), Penaea acutifolia (3), Pentameris uniflora (1), Pentaschistis angustifolia (1), Pentaschistis barbata ssp orientalis (1), Pentaschistis heptamera (7), Pentaschistis longipes (1), Polygala bowkerae (1), Prismatocarpus hispidus (2), Psoralea keetii (2), Pterygodium newdigitatae (5), Restio fourcadei (6), Rhodocoma gracilis (2), Senecio hirtifolius (1), Senecio serrurioides (1), Stapelia obducta (1), Stoebe ensorii (2), Syringodea flanaganii (1), Tetraria robusta (1), Trichodiadema rupicolum (1)*5.1. West Outeniekwaberg
*Acmadenia gracilis (1), Acmadenia maculata (2), Acmadenia rupicola (1), Adromischus mammillaris (1), Anisodontea alexandri (1), Aspalathus digitifolia (2), Aspalathus glabrescens (2), Diascia pentheri (1), Disa arida (1), Erica curviflora var. splendens (1), Erica inflaticalyx (2), Erica velatiflora (2), Leucadendron elens (2), Leucadendron olens (2), Leucospermum hamatum (1), Lobelia dichroma (1), Lobelia montaguensis (1), Manulea derustiana (1), Mimetes chrysanthus (2), Oxalis stellata var. gracilior (1), Paranomus spathulatus type gamka (1), Pentameris uniflora (1), Prismatocarpus hispidus (2), Rhodocoma gracilis (2)*5.2. Port Elizabeth Peninsula
*Agathosma stenopetala (4), Aspalathus cliffortiifolia (2), Aspalathus lanceicarpa (2), Aspalathus recurvispina (4), Brachystelma schoenlandianum (1), Brunsvigia litoralis (1), Cyclopia filiformis (1), Cyclopia longifolia (1), Cyrtanthus spiralis (1), Cyrtanthus staadensis (1), Erica etheliae (1), Euphorbia meloformis (1), Euryops ursinoides (1), Gladiolus alatus var. algoensis (1), Huernia longii (2), Lachnaea glomerata (2), Lobelia zwartkopensis (1), Orthopterum coegana (1), Othonna membranifolia (3), Polygala bowkerae (1), Senecio hirtifolius (1), Senecio serrurioides (1), Syringodea flanaganii (1), Trichodiadema rupicolum (1)*5.3. East Outeniekwaberg
*Acmadenia alternifolia (5), Agathosma alaris (1), Aspalathus hirta stellaris (1), Erica coronanthera (4), Erica curviflora var. sulcata (1), Erica keetii (2), Erica laevigata var. elongata (1), Faurea macnaughtonii (3), Herschelia newdigatae (1), Herschelianthe newdigateae (1), Pentaschistis barbata ssp orientalis (1), Tetraria robusta (1)*5.4. Tsitsikammaberg
*Aspalathus teres thodei (2), Erica adaequata (1), Erica beatricis (1), Erica humansdorpensis (3), Erica jeppei (1), Erica zitzikammensis (2), Felicia jourbertinae ssp joubertinae (1), Felicia tsitsikamae (1), Helichrysum outeniquense (1), Stoebe ensorii (2)*5.5. West Albany Centre
*Agathosma clavisepala (4), Aspalathus argyrophanes (2), Erica varderi (1), Euryops polythricoides (2), Muraltia lancifolia (2), Oldenburgia grandis (6), Pentaschistis angustifolia (1)*5.6. Groot-Winterheokberge
*Agathosma citriodora (1), Encephalartos longifolius (1), Euchaetis cristagalli (2), Stapelia obducta (1)*5.7. Cockscomb
*Cyrtanthus flammosus (1), Gasteria ellaphieae (1), Gasteria glomerata (1)*5.8. Oosterbaai
*Pentaschistis longipes (1)*5.9. East London
*Bobartia gracilis (1)*5.10. Kiwane
*Acmadenia kiwanensis (1)*

6. Agulhas Plains
*Acmadenia mundiana (2), Adenandra gummifera (2), Adenandra obtusa (5), Adenandra odoratissima (1), Adenandra odoratissima ssp tenuis (1), Adenandra rotundifolia (5), Adenandra schlechteri (2), Agathosma abrupta (2), Agathosma collina (7), Agathosma florulenta (1), Agathosma haelkraalensis (2), Agathosma joubertina (1), Agathosma paralia (2), Agathosma rotundifolia (1), Agathosma sedifolia (4), Aspalathus aciloba (4), Aspalathus barbigera (3), Aspalathus potbergensis (2), Berzelia cordifolia (3), Bobartia longicyma subsp. microflora (3), Calopsis pulchra (4), Ceratocaryum pulchrum (1), Cliffortia burgersii (2), Cliffortia curvifolia (2), Cliffortia incana (2), Cyrtanthus guthrieae (2), Delosperma mariae (1), Diosma arenicola (4), Diosma awilana (2), Diosma guthriei (6), Diosma haelkraalensis (1), Diosma parvula (3), Erepsia polypetala (2), Erica aghilliana (3), Erica albertyniae (5), Erica bredasiana (4), Erica brownii (3), Erica bruniifolia var. subglabra (1), Erica calcareophila (1), Erica capillaris var. compacta (1), Erica casta var. breviflora (1), Erica filipendula var. major (2), Erica flavicoma (2), Erica globulifera (5), Erica globuliflora (5), Erica innovans (2), Erica interrupta (2), Erica oblongiflora (4), Erica occulta (2), Erica regia var. williana (1), Erica uysii (1), Erica venustiflora subsp. glandulosa (1), Erica venustiflora subsp. venustiflora (1), Euchaetis diosmoides (5), Euchaetis intonsa (1), Euchaetis laevigata (2), Euchaetis longibracteata (6), Euchaetis meridionalis (6), Euchaetis scabricosta (5), Felicia canaliculata (3), Felicia nordenstamii (3), Freesia elimensis (1), Galium bredasdorpense (1), Hypodiscus procurrens (3), Ischyrolepis anomala (2), Lachenalia dehoopensis (2), Lachenalia sargeantii (1), Leucadendron stelligerum (2), Leucospermum fulgens (2), Metalasia umbelliformis (3), Mimetes saxatilis (6), Muraltia calycina (4), Muraltia lewisiae (3), Muraltia pottebergensis (1), Osteospermum elsieae (1), Osteospermum hafstroemii (1), Osteospermum subulatum (3), Otholobium pungens (1), Phylica amoena (1), Phylica floribunda (2), Phylica incurvata (1), Phylica laevifolia (1), Phylica lasiantha (1), Polhillia canescens (1), Polygala dasyphylla (2), Polygala pottebergensis (2), Prismatocarpus spinosus (1), Protea denticulata (2), Protea pudens (3), Protea triandra (1), Psoralea pungens (1), Pteronia diosmifolia (2), Restio dodii var purpureus (3), Roella cuspidata var hispida (1), Roella rhodantha (1), Serruria ventricosa (1), Stoebe cyathuloides (4), Stoebe schultzii (2), Trichodiadema pygmaeum (1), Wahlenbergia microphylla (1), Xiphotheca guthriei (1)*6.1. West Agulhas Plains
*Adenandra obtusa (5), Adenandra odoratissima (1), Adenandra odoratissima ssp tenuis (1), Adenandra schlechteri (2), Agathosma abrupta (2), Agathosma florulenta (1), Agathosma haelkraalensis (2), Agathosma joubertina (1), Agathosma sedifolia (4), Aspalathus aciloba (4), Calopsis pulchra (4), Ceratocaryum pulchrum (1), Cliffortia curvifolia (2), Cyrtanthus guthrieae (2), Diosma arenicola (4), Diosma awilana (2), Diosma guthriei (6), Diosma haelkraalensis (1), Erepsia polypetala (2), Erica aghilliana (3), Erica bredasiana (4), Erica brownii (3), Erica bruniifolia var. subglabra (1), Erica calcareophila (1), Erica capillaris var. compacta (1), Erica casta var. breviflora (1), Erica filipendula var. major (2), Erica flavicoma (2), Erica innovans (2), Erica interrupta (2), Erica occulta (2), Erica regia var. williana (1), Erica venustiflora subsp. venustiflora (1), Felicia canaliculata (3), Freesia elimensis (1), Hypodiscus procurrens (3), Ischyrolepis anomala (2), Lachenalia sargeantii (1), Leucadendron stelligerum (2), Metalasia umbelliformis (3), Mimetes saxatilis (6), Osteospermum hafstroemii (1), Osteospermum subulatum (3), Phylica amoena (1), Phylica floribunda (2), Phylica incurvata (1), Phylica laevifolia (1), Polygala dasyphylla (2), Protea pudens (3), Protea triandra (1), Restio dodii var purpureus (3), Roella cuspidata var hispida (1), Stoebe schultzii (2), Wahlenbergia microphylla (1), Xiphotheca guthriei (1)*6.2. Potberg
*Acmadenia mundiana (2), Adenandra gummifera (2), Agathosma paralia (2), Agathosma rotundifolia (1), Aspalathus barbigera (3), Aspalathus potbergensis (2), Bobartia longicyma subsp. microflora (3), Cliffortia burgersii (2), Cliffortia incana (2), Delosperma mariae (1), Erica uysii (1), Erica venustiflora subsp. glandulosa (1), Euchaetis intonsa (1), Galium bredasdorpense (1), Lachenalia dehoopensis (2), Leucospermum fulgens (2), Muraltia pottebergensis (1), Osteospermum elsieae (1), Otholobium pungens (1), Phylica lasiantha (1), Polhillia canescens (1), Polygala pottebergensis (2), Prismatocarpus spinosus (1), Protea denticulata (2), Psoralea pungens (1), Roella rhodantha (1), Serruria ventricosa (1), Trichodiadema pygmaeum (1)*

7. Nieuwoudtville
*Adromischus subviridis (1), Aloe buhrii (1), Aloe khamiesensis (1), Amphisiphon stylosa (1), Androsiphon capense (1), Aspalathus isolata (2), Aspalathus obliqua (1), Aspalathus proboscidea (1), Athanasia hirsuta (1), Babiana pauciflora (3), Chamarea snijmaniae (1), Coelidium minimum (1), Conophytum acutum (1), Conophytum swanepoelianum subsp. swanepoelianum (1), Corycium ingeanum (2), Cotula loganii (1), Crassula pellucida ssp spongiosa (1), Cyanella aquatica (1), Diascia insignis (1), Diascia lewisiae (1), Eriospermum erinum (2), Eriospermum glaciale (2), Euphorbia brakdamensis (1), Euphorbia cylindrica (1), Euryops marlothii (1), Euryops mirus (1), Euryops rosulatus (2), Euryops vaginatus (2), Euryops virgatus (1), Geissorhiza inaequalis (1), Geissorhiza splendidissima (1), Geissorhiza subrigida (1), Gladiolus mostertiae (1), Gladiolus pritzelii var. sufflavus (3), Gnidia leipoldtii (1), Gynandriris hesperantha (1), Hesperantha rivulicola (1), Hessea pusilla (1), Homeria odorata (1), Homeria spiralis (2), Ixia brunneobracteata (1), Lachenalia dasybotrya (1), Lachenalia macgregoriorum (1), Lapeirousia oreogena (1), Leucadendron meyerianum (1), Leucadendron remotum (3), Lithops comptonii var. weberi (1), Lotononis carnea (1), Moraea macgregorii (1), Moraea verecunda (1), Nemesia chrysolopha (2), Ornithogalum pilosum subsp. pullatum (1), Othonna rechingeri (2), Oxalis lasiorrhiza (1), Oxalis melanosticta var. latifolia (1), Oxalis pulvinata (1), Pelargonium connivens (1), Phylica agathosmoides (1), Prionanthium dentatum (1), Romulea monadelpha (1), Romulea sanguinalis (1), Romulea vanzyliae (2), Sparaxis elegans (1), Sparaxis pillansii (1), Staavia phylicoides (1), Strumaria discifera subsp. bulbifera (1), Strumaria perryae (1), Strumaria picta (2), Sutera longipedicellata (1), Trachyandra prolifera (1), Vellereophyton gracillium (1), Xiphotheca canescens (1), Zaluzianskya acrobareia (1), Zantedeschia odorata (1)*7.1. Nieuwoudtville Core
*Adromischus subviridis (1), Aloe buhrii (1), Aloe khamiesensis (1), Amphisiphon stylosa (1), Androsiphon capense (1), Aspalathus isolata (2), Aspalathus obliqua (1), Aspalathus proboscidea (1), Athanasia hirsuta (1), Chamarea snijmaniae (1), Coelidium minimum (1), Conophytum acutum (1), Conophytum swanepoelianum subsp. swanepoelianum (1), Corycium ingeanum (2), Cotula loganii (1), Crassula pellucida ssp spongiosa (1), Cyanella aquatica (1), Diascia insignis (1), Diascia lewisiae (1), Eriospermum erinum (2), Eriospermum glaciale (2), Euphorbia brakdamensis (1), Euphorbia cylindrica (1), Euryops mirus (1), Euryops rosulatus (2), Euryops vaginatus (2), Euryops virgatus (1), Geissorhiza inaequalis (1), Geissorhiza splendidissima (1), Geissorhiza subrigida (1), Gladiolus mostertiae (1), Gladiolus pritzelii var. sufflavus (3), Gnidia leipoldtii (1), Gynandriris hesperantha (1), Hesperantha rivulicola (1), Hessea pusilla (1), Homeria odorata (1), Homeria spiralis (2), Ixia brunneobracteata (1), Lachenalia dasybotrya (1), Lachenalia macgregoriorum (1), Lapeirousia oreogena (1), Leucadendron meyerianum (1), Leucadendron remotum (3), Lotononis carnea (1), Moraea macgregorii (1), Moraea verecunda (1), Ornithogalum pilosum subsp. pullatum (1), Othonna rechingeri (2), Oxalis lasiorrhiza (1), Oxalis melanosticta var. latifolia (1), Oxalis pulvinata (1), Phylica agathosmoides (1), Prionanthium dentatum (1), Romulea monadelpha (1), Romulea sanguinalis (1), Romulea vanzyliae (2), Sparaxis elegans (1), Sparaxis pillansii (1), Staavia phylicoides (1), Strumaria discifera subsp. bulbifera (1), Strumaria perryae (1), Strumaria picta (2), Sutera longipedicellata (1), Trachyandra prolifera (1), Vellereophyton gracillium (1), Xiphotheca canescens (1), Zaluzianskya acrobareia (1), Zantedeschia odorata (1)*7.2. Rooiberg
*Euryops marlothii (1), Pelargonium connivens (1)*7.3. Central Tankwa
*Lithops comptonii var. weberi (1)*

8. Groot-Winterhoek - Skurweberg
*Acmadenia macradenia (1), Agathosma alligans (1), Agathosma concava (1), Agathosma insignis (2), Agathosma tulbaghensis (2), Alciope lanata (3), Anaxeton angustifolium (1), Anthochortus insignis (2), Aspalathus amoena (1), Aspalathus compacta (1), Aspalathus corniculata (2), Aspalathus densifolia (3), Aspalathus erythrodes (1), Aspalathus fasciculata (1), Aspalathus juniperina gracilifolia (2), Aspalathus lenticula (1), Aspalathus secunda (1), Aspalathus spinosissima tenuiflora (1), Aspalathus suaveolens (1), Aspalathus sulphurea (3), Aspalathus tulbaghensis (1), Aspalathus ulicina ulicina (3), Athanasia elsiae (1), Bulbine monophylla (1), Comborhiza longipes (2), Diastella myrtifolia (2), Elegia fastigiata (1), Erepsia babiloniae (1), Erica articularis var. implexa (1), Erica auriculata (1), Erica cupuliflora (1), Erica cuscutiformis (1), Erica daphniflora var. latisepala (1), Erica daphniflora var. retusa (5), Erica denticulata var. grandiloba (1), Erica haemantha (1), Erica irrorata (3), Erica longifolia var. viridis (4), Erica lucida var. pauciflora (1), Erica micrandra (2), Erica orculiflora (1), Erica sessiliflora var. sceptriformis (2), Geissorhiza esterhuyseniae (1), Geissorhiza rupicola (1), Geissorhiza silenoides (1), Haplocarpha parvifolia (1), Huernia witzenbergensis (1), Ischyrolepis coactilis (1), Lachenalia polyphylla (2), Leucadendron gydoense (4), Lobelia humifusa (1), Lonchostoma myrtoides (3), Metalasia rogersii (2), Metalasia serrulata (2), Moraea incurva (1), Muraltia angustiflora (1), Oxalis involuta (2), Pentaschistis caulescens (2), Pentaschistis dregeana (1), Pentaschistis involuta (1), Phylica ampliata (1), Phylica trachyphylla (1), Protea mucronifolia (1), Rafnia crispa (1), Raspalia sp. (1), Steirodiscus gamolepis (1), Stoebe montana (1), Thamnea hirtella (2), Ursinia merxmuelleri (1), Watsonia dubia (1)*8.1. Groot-Winterhoek - Skurweberg Core
*Acmadenia macradenia (1), Agathosma alligans (1), Agathosma concava (1), Agathosma insignis (2), Agathosma tulbaghensis (2), Alciope lanata (3), Anaxeton angustifolium (1), Anthochortus insignis (2), Aspalathus amoena (1), Aspalathus compacta (1), Aspalathus corniculata (2), Aspalathus densifolia (3), Aspalathus erythrodes (1), Aspalathus fasciculata (1), Aspalathus juniperina gracilifolia (2), Aspalathus lenticula (1), Aspalathus spinosissima tenuiflora (1), Aspalathus suaveolens (1), Aspalathus sulphurea (3), Aspalathus tulbaghensis (1), Aspalathus ulicina ulicina (3), Athanasia elsiae (1), Comborhiza longipes (2), Diastella myrtifolia (2), Elegia fastigiata (1), Erepsia babiloniae (1), Erica articularis var. implexa (1), Erica auriculata (1), Erica cupuliflora (1), Erica cuscutiformis (1), Erica daphniflora var. latisepala (1), Erica denticulata var. grandiloba (1), Erica haemantha (1), Erica irrorata (3), Erica lucida var. pauciflora (1), Erica micrandra (2), Erica orculiflora (1), Erica sessiliflora var. sceptriformis (2), Geissorhiza esterhuyseniae (1), Geissorhiza rupicola (1), Geissorhiza silenoides (1), Haplocarpha parvifolia (1), Huernia witzenbergensis (1), Ischyrolepis coactilis (1), Lachenalia polyphylla (2), Leucadendron gydoense (4), Lobelia humifusa (1), Lonchostoma myrtoides (3), Metalasia rogersii (2), Moraea incurva (1), Muraltia angustiflora (1), Pentaschistis caulescens (2), Pentaschistis dregeana (1), Pentaschistis involuta (1), Phylica ampliata (1), Phylica trachyphylla (1), Protea mucronifolia (1), Rafnia crispa (1), Raspalia sp. (1), Steirodiscus gamolepis (1), Stoebe montana (1), Thamnea hirtella (2), Ursinia merxmuelleri (1), Watsonia dubia (1)*8.2. Heuningberg
*Bulbine monophylla (1)*8.3. Kasteelberg
*Aspalathus secunda (1)*

9. Boland Mountains
*Agathosma decurrens (1), Agathosma propinqua (2), Agathosma stenosepala (1), Berzelia incurva (2), Cliffortia gracilis (1), Cliffortia pilifera (1), Cliffortia polygonifolia var membranifolia (1), Cliffortia rigida (1), Cliffortia strigosa (1), Cliffortia subdura (1), Diastella buekii (2), Disa brendae (1), Drosera regia (1), Erepsia insignis (1), Erepsia lacera (3), Erica alexandri (2), Erica calycina var. viscidiflora (3), Erica cymosa (2), Erica hippurus (1), Erica lerouxiae (2), Erica pinea var. argentiflora (1), Erica tenuipes (2), Gladiolus citrinus (2), Gladiolus nerineoides (1), Haemanthus pumilio (2), Heliophila cuneata (1), Ixia cochlearis (2), Leucadendron comosum subsp. homaeophyllum (1), Macrostylis villosa ssp minor (1), Marasmodes undulata (1), Metalasia schlechteri (= Planea schlechteri) (1), Nemesia picta (1), Osmitopsis pinnitifida ssp pinnatifida (3), Osmitopsis pinnitifida ssp serrata (1), Osmitopsis tenuis (1), Osteospermum hispidum var viride (1), Phylica guthriei (1), Phylica nodosa (1), Planea schlechteri (2), Pteronia centauroides (1), Rafnia ericifolia (2), Restio alticola (2), Restio montanus (1), Restio obscurus (2), Restio singularis (2), Scilla plumbea (1), Senecio anapetes (2), Stylapterus ericoides ssp pallidus (1), Thamnea uniflora (1), Ursinia filipes (3)*

10. Northern NWPC
*Agathosma dregeana (1), Aloe falcata (1), Athanasia leptocephala (1), Babiana klaverensis (1), Babiana mucronata var. minor (2), Centella cochlearia (1), Chlorophytum lewisiae (1), Elytropappus hispidus (1), Erica sonora (4), Eriospermum eriophorum (1), Eriospermum minutipustulatum (1), Euphorbia hallii (1), Euphorbia pedemontana (1), Gethyllis latifolia (1), Gladiolus comptonii (1), Haemanthus amarylloides subsp. toximontanus (1), Haemanthus lanceifolius (3), Haemanthus pubescens subsp. leipoldtii (3), Haworthia nortieri var. globosiflora (2), Hessea undosa (3), Laurentia giftbergensis (1), Leucadendron roodii (4), Manulea ramulosa (1), Ornithogalum inclusum (1), Ornithoglossum parviflorum var namaquense (1), Othonna papaveroides (3), Oxalis blastorrhiza (1), Oxalis creaseyi (1), Oxalis oligophylla (1), Oxalis oreithala (1), Oxalis tenuis (1), Pelargonium crassipes (1), Pelargonium nephrophyllum (2), Polycarena nardouwensis (1), Polygala lasiosepala (1), Quaqua framesii (2), Rhynchosia arida (1), Romulea sladenii (2), Serruria lacunosa (2), Sparaxis caryophyllacea (1), Steirodiscus schlechteri (1), Strumaria unguiculata (1), Strumaria watermeyeri subsp. botterkloofensis (1), Trachyandra zebrina (2), Vellereophyton pulvinatum (1), Wahlenbergia asperifolia (1), Wahlenbergia longisepala (1), Wiborgia humilis (2)*10.1. Gifberg-Matsikammaberg
*Agathosma dregeana (1), Aloe falcata (1), Athanasia leptocephala (1), Babiana klaverensis (1), Babiana mucronata var. minor (2), Centella cochlearia (1), Elytropappus hispidus (1), Erica sonora (4), Eriospermum eriophorum (1), Eriospermum minutipustulatum (1), Euphorbia pedemontana (1), Gethyllis latifolia (1), Gladiolus comptonii (1), Haemanthus amarylloides subsp. toximontanus (1), Haemanthus pubescens subsp. leipoldtii (3), Hessea undosa (3), Laurentia giftbergensis (1), Leucadendron roodii (4), Manulea ramulosa (1), Ornithoglossum parviflorum var namaquense (1), Othonna papaveroides (3), Oxalis blastorrhiza (1), Oxalis creaseyi (1), Oxalis oligophylla (1), Oxalis oreithala (1), Oxalis tenuis (1), Pelargonium crassipes (1), Pelargonium nephrophyllum (2), Polycarena nardouwensis (1), Polygala lasiosepala (1), Quaqua framesii (2), Rhynchosia arida (1), Romulea sladenii (2), Serruria lacunosa (2), Sparaxis caryophyllacea (1), Steirodiscus schlechteri (1), Trachyandra zebrina (2), Vellereophyton pulvinatum (1), Wahlenbergia asperifolia (1), Wahlenbergia longisepala (1), Wiborgia humilis (2)*10.2. Boegoeberge
*Chlorophytum lewisiae (1), Euphorbia hallii (1), Haworthia nortieri var. globosiflora (2), Ornithogalum inclusum (1), Strumaria unguiculata (1), Strumaria watermeyeri subsp. botterkloofensis (1)*

11. Central Cedarberg
*Acmadenia bodkinii (1), Acmadenia rourkeana (1), Agathosma conferta (1), Agathosma distans (1), Agathosma pattisoniae (1), Agathosma pubigera (4), Agathosma rubricaulis (2), Agathosma viviersii (1), Androcymbium scabromarginatum (1), Aspalathus bidouwensis (2), Aspalathus comptonii (2), Aspalathus polycephala lanatofolia (1), Aspalathus polycephala polycephala (1), Athanasia bremeri (2), Athanasia calophylla (1), Athanasia microphylla (2), Athanasia pachycephala ssp. eriopoda = Athanasia eriopoda (3), Babiana geniculata (1), Crassula elsieae (2), Cyphia ranunculifolia (1), Disa cedarbergensis (1), Erica consobrina (1), Erica eremioides subsp. eglandula (3), Erica longipedunculata var. setifera (1), Erica oresigena var. mollipila (1), Euryops wageneri (4), Gladiolus buckerveldii (1), Helichrysum aureofolium (2), Heliophila cedarbergensis (2), Lachenalia maximiliani (4), Metalasia albescence (1), Oxalis porphyriosiphon (1), Pentaschistis pyrophilla (1), Phylica alpina (1), Phylica altigena (1), Phylica barbata (1), Phylica maximilianii (1), Prismatocarpus decurrens (1), Pteronia ambrariifolia (1), Romulea sulphurea (1), Spiloxene umbraticola (1), Tritoniopsis latifolia (1), Vellereophyton lasianthum (1), Wahlenbergia adamsonii (1)*

12. Piketberg/Olifantsberge and Northern Sandveld
*Agathosma involucrata (3), Aspalathus chrysantha (1), Aspalathus complicata (3), Aspalathus glossoides (1), Aspalathus latifolia (3), Aspalathus pendula (2), Athanasia pubescens (2), Bobartia orientalis subsp. occidentalis (3), Cephalophyllum parvulum (1), Corymbium theileri (1), Cullumia floccosa (4), Cullumia micracantha (2), Diplosoma retroversum (4), Elytropappus intricata (2), Erepsia pillansii (5), Erica dregei (3), Erica piquetbergensis (2), Euchaetis tricarpellata (1), Euryops pectinatus ssp lobulatus (3), Geissorhiza brevituba (1), Hermannia cordifolia (2), Hermannia hispidula (2), Hesperantha pallescens (1), Ixia splendida (1), Lachenalia thomasiae (1), Leucospermum profugum (5), Lotononis racemiflora (1), Macrostylis cassiopoides ssp cassiopoides (2), Macrostylis hirta (2), Moraea gigandra (1), Pentaschistis rosea ssp purpurascens (1), Romulea tortilis var. dissecta (1), Schizodium antenniferum (1), Sparaxis roxburghii (1), Stapelia immelmaniae (2), Wahlenbergia constricta (1), Wahlenbergia distincta (1), Wahlenbergia massonii (1), Wahlenbergia subtilis (1), Wahlenbergia tomentosula (1)*12.1. Piketberg
*Aspalathus chrysantha (1), Aspalathus complicata (3), Aspalathus glossoides (1), Aspalathus latifolia (3), Athanasia pubescens (2), Bobartia orientalis subsp. occidentalis (3), Corymbium theileri (1), Elytropappus intricata (2), Erica piquetbergensis (2), Euchaetis tricarpellata (1), Euryops pectinatus ssp lobulatus (3), Geissorhiza brevituba (1), Hermannia cordifolia (2), Hermannia hispidula (2), Hesperantha pallescens (1), Ixia splendida (1), Moraea gigandra (1), Pentaschistis rosea ssp purpurascens (1), Romulea tortilis var. dissecta (1), Schizodium antenniferum (1), Stapelia immelmaniae (2), Wahlenbergia massonii (1), Wahlenbergia subtilis (1), Wahlenbergia tomentosula (1)*12.2. North Sandveld
*Agathosma involucrata (3), Cephalophyllum parvulum (1), Lachenalia thomasiae (1), Lotononis racemiflora (1), Macrostylis cassiopoides ssp cassiopoides (2), Macrostylis hirta (2), Sparaxis roxburghii (1), Wahlenbergia constricta (1), Wahlenbergia distincta (1)*

13. Vanrhynsdorp Plains
*Argyroderma framesii subsp. framesii (1), Argyroderma ringens (1), Argyroderma subalbum (2), Argyroderma testiculare (1), Babiana lewisiana (2), Babiana stenomera (1), Bulbine haworthioides (2), Bulbine louwii (1), Bulbine margarethae (1), Bulbine wiesei (1), Cephalophyllum pulchellum (2), Conophytum uviforme subsp. subincanum (1), Eriospermum arachnoideum (1), Eriospermum attenuatum (1), Eriospermum calcareum (2), Eriospermum fragile (1), Eriospermum titanopsoides (1), Euphorbia lumbricalis (1), Homeria ramosissima (1), Ixia acaulis (1), Lasiopogon minutus (2), Limonium teretifolium (2), Lithops divergens var. divergens (1), Oedera silicicola (1), Ornithogalum hallii (1), Ornithogalum naviculum (2), Othonna cakilifolia (1), Othonna hallii (2), Oxalis senecta (2), Strumaria pygmaea (1), Tylecodon suffultus (1), Tylecodon tenuis (2), Zygophyllum teretifolium (2)*

14. Hexrivierberge
*Anderbergia ustulata (1), Aspalathus keeromsbergensis (1), Aspalathus orbiculata (1), Aspalathus pachyloda rugulicarpa (1), Aspalathus pilantha (1), Aspalathus shawii longispica (1), Athanasia alba (1), Cliffortia lanata (1), Disparago barbata (1), Drosanthemum bellum (1), Drosanthemum hallii (1), Drosanthemum thudichumii var. gracilius (1), Erica curviflora var. versatilis (1), Erica keeromsbergensis (1), Heliophila filicaulis (1), Ixia pumilio (1), Moraea worcesterensis (1), Muraltia serrata (2), Nerine sp. (1), Oxalis henrici (1), Pelargonium lanceolatum (2), Phylica reversa (1), Protea holosericea (1), Restio rupicola (1), Tenicroa planifolia (1)*

15. Saldanha Peninsula
*Aloe arenicola (2), Aloe distans (2), Aspalathus gerrardii (1), Empodium occidentale (5), Felicia elongata (3), Freylinia visseri (1), Gladiolus gracilis var. latifolius (3), Hesperantha saldanhae (1), Ixia purpureorosea (3), Lachenalia viridiflora (2), Limonium acuminatum (5), Limonium capense (4), Manulea augei (1), Moraea calcicola (1), Moraea loubseri (1), Oncosiphon schlechteri (1), Ornithogalum rupestre (3), Oxalis burtoniae (2), Pauridia longituba (3), Pelargonium appendiculatum (1), Phylica greyii (1), Romulea barkerae (1), Romulea elliptica (1), Wahlenbergia umbellata (1), Watsonia hysterantha (2)*15.1. Saldanha Core
*Aspalathus gerrardii (1), Felicia elongata (3), Gladiolus gracilis var. latifolius (3), Hesperantha saldanhae (1), Lachenalia viridiflora (2), Moraea calcicola (1), Moraea loubseri (1), Ornithogalum rupestre (3), Oxalis burtoniae (2), Pauridia longituba (3), Phylica greyii (1), Romulea barkerae (1), Romulea elliptica (1), Watsonia hysterantha (2)*15.2. Lambert's Bay
*Oncosiphon schlechteri (1), Pelargonium appendiculatum (1), Wahlenbergia umbellata (1)*15.3. Hopefield
*Manulea augei (1)*15.4. Aurora
*Freylinia visseri (1)*

16. West Langeberg - Waboomsberg
*Agathosma subteretifolia (2), Albuca scabra (1), Anderbergia vlokii (1), Anisothrix kuntze (2), Arctotis stoechadifolia (1), Aspalathus aemula (deviating forms) (1), Aspalathus rostrata (2), Aspalathus shawii globripetala (1), Crassula subulata var hispida (1), Erica glandulipila (1), Felicia comptonii (1), Gladiolus stefaniae (1), Haworthia poellnitziana (1), Homeria radians (2), Ixia stolonifera (1), Oxalis ciliaris var. pageae (1), Oxalis microdonta (3), Pentaschistis rigidissima ssp horridus (1), Romulea malaniae (1), Vexatorella latebrosa (1), Wurmbea compacta (1)*

17. Witteberg
*Acmadenia argillophila (1), Adenandra dahlgrenii (2), Agathosma acocksii (2), Aspalathus intricata anthospermoides (2), Cliffortia conifera (1), Crotalaria lebeckioides (1), Erica loganii (1), Euryops microphyllus (2), Hypodiscus sulcatus (5), Lotononis gracilifolia (1), Muraltia bondii (1), Nenax sp (1), Ornithogalum diluculum (1), Othonna lepidocaulis (1), Phylica retorta (3), Romulea syringodeoflora (1), Senecio wittebergensis (2)*17.1. Witteberg Core
*Acmadenia argillophila (1), Agathosma acocksii (2), Aspalathus intricata anthospermoides (2), Cliffortia conifera (1), Crotalaria lebeckioides (1), Erica loganii (1), Hypodiscus sulcatus (5), Lotononis gracilifolia (1), Nenax sp (1), Ornithogalum diluculum (1), Othonna lepidocaulis (1), Phylica retorta (3), Romulea syringodeoflora (1), Senecio wittebergensis (2)*17.2. Towerkop
*Muraltia bondii (1)*

18. NE Escarpment
*Bonatea liparophylla (1), Bonatea saundersioides (2), Bonatea transvaalensis (4), Disa alticola (2), Disa amoena (1), Disa aristata (2), Disa extinctoria (5), Disa intermedia (1), Erica atherstonei (5), Erica cerinthoides var. barbertona (2), Eulophia chlorantha (2), Habenaria barbertonii (1), Pentaschistis chippindalliae (5), Schizochilus cecili ssp culveri (4), Schizochilus cecili ssp transvaalensis (3), Schizochilus crenulatus (2), Schizochilus lilacinus (2)*18.1. NE Escarpment Core
*Disa alticola (2), Disa amoena (1), Erica atherstonei (5), Schizochilus cecili ssp transvaalensis (3), Schizochilus crenulatus (2), Schizochilus lilacinus (2)*18.2. Barberton-Lebombo
*Bonatea liparophylla (1), Bonatea saundersioides (2), Disa intermedia (1), Eulophia chlorantha (2), Habenaria barbertonii (1), Schizochilus cecili ssp culveri (4)*18.3. Wolkeberg-Soutpansberg
*Bonatea transvaalensis (4), Disa aristata (2)*

19. Kamiesberg
*Agathosma namaquensis (4), Amellus alternifolius ssp. alternifolius (5), Amphiglossa celans (2), Antithrixia flaviconia (3), Aspalathus angustifolia robusta (2), Chrysocoma tomentosa (3), Disa macrostachya (1), Erica dilatata (1), Felicia diffusa ssp. khamiesbergensis (1), Ferraria uncinata macrochlamys (6), Haemanthus amarylloides polyanthus (6), Haemanthus ganiticus (2), Ischyrolepis vilis (2), Pentaschistis filifolia (1), Pentaschistis lima (2), Protea namaquana (1), Vexatorella alpina (1)*19.1. Kamiesberg Core
*Agathosma namaquensis (4), Antithrixia flaviconia (3), Aspalathus angustifolia robusta (2), Chrysocoma tomentosa (3), Disa macrostachya (1), Erica dilatata (1), Felicia diffusa ssp. khamiesbergensis (1), Haemanthus ganiticus (2), Ischyrolepis vilis (2), Pentaschistis filifolia (1), Pentaschistis lima (2), Protea namaquana (1), Vexatorella alpina (1)*19.2. Garies
*Amphiglossa celans (2)*

20. Eastern Drakensberg
*Athanasia grandiceps (2), Disa cephalotes ssp frigida (2), Erica aestiva (6), Erica ebracteata (4), Erica lasiocarpa (2), Erica straussiana (9), Erica symonsii (2), Erica tricholada (4), Erica wyliei (4), Merxmuellera aureocephala (4), Pentaschistis merxmuelleri (2), Pentaschistis pilosogluma (7), Pentaschistis praecox (3)*20.1. Eastern Drakensberg Core
*Disa cephalotes ssp frigida (2), Erica lasiocarpa (2), Pentaschistis merxmuelleri (2)*20.2. Southern Drakensberg
*Athanasia grandiceps (2), Erica symonsii (2), Pentaschistis praecox (3)*20.3. Natal Midlands
*Erica tricholada (4)*

21. Lainsberg
*Adromischus liebenbergii (2), Aloinopsis loganii (1), Cleretum lyratifolium (1), Geissorhiza karooica (1), Haworthia pehlemanniae (1), Hesperantha flava (1), Homeria fenestrata (1), Pelargonium torulosum (1), Polycarena comptonii (1), Quaqua multiflora (1), Strumaria pubescens (1), Tanquana archeri (2), Trachyandra thyrsoidea (2)*

22. Northern Southeastern Centre
*Ceropegia cancellata (2), Crassula arborescens ssp undulatifolia (1), Euphorbia albipollinifera (2), Euphorbia bruynsii (1), Euphorbia jansenvillensis (1), Euphorbia valida (1), Euryops latifolius (1), Haworthia bruynsii (2), Haworthia springbokvlakensis (3), Pelargonium exhibens (1), Pleiospilos compactus subsp. minor (1), Senecio scaposus var. addoensis (1), Stapeliopsis pillansii (2)*22.1. Wolwefontein
*Euphorbia albipollinifera (2), Euphorbia bruynsii (1), Euphorbia jansenvillensis (1), Euphorbia valida (1), Euryops latifolius (1), Haworthia bruynsii (2), Haworthia springbokvlakensis (3), Pelargonium exhibens (1), Pleiospilos compactus subsp. minor (1)*22.2. Sunday's River Valley
*Ceropegia cancellata (2), Crassula arborescens ssp undulatifolia (1), Senecio scaposus var. addoensis (1)*

23. Kouebokkeveld
*Agathosma maculata (1), Aspalathus spinosissima (intermediate (1), Aspalathus ulicina kardouwensis (1), Crassula alcicornis (1), Geissorhiza louisabolusiae (3), Gladiolus delpierrei (1), Heterorhachis sp 1 (1), Macrostylis barbigera (2), Macrostylis ramulosa (1), Manulea psilostoma (1), Phylica salteri (1), Romulea tortilis var. tortilis (1)*

24. East Riversdale Plains
*Agathosma muirii (4), Agathosma pallens (1), Agathosma robusta (1), Aspalathus candicula (2), Aspalathus sanguinea foliosa (3), Athanasia quinquedentata ssp. ringens (1), Euphorbia corymbosa (1), Euryops muirii (1), Metalasia luteola (1), Oedera steyniae (2), Phylica laevigata (1), Relhania steyniae (3)*

25. Skurweberg - Swartrugberg
*Agathosma digitata (1), Eriospermum algiferum (1), Homeria fuscomontana (1), Metalasia phillipsii ssp. incurva = Metalasia incurva (2), Oedera foveolata (1), Relhania foveolata (1), Rhodocoma vleibergensis? (2), Strumaria karoopoortensis (1), Zaluzianskya lanigera (1)*

26. Natal Coastal Centre
*Cynorkis compacta (3), Diaphananthe millarii (3), Disperis woodii (5), Erica aspalatifolia (12), Habenaria pseudociliosa (4), Helichrysum isolepsis (2), Mystacidium pusillum (2), Stenoglottis longifolia (2)*26.1. Natal Coastal Centre
*Cynorkis compacta (3), Diaphananthe millarii (3), Disperis woodii (5), Erica aspalatifolia (12), Habenaria pseudociliosa (4), Mystacidium pusillum (2), Stenoglottis longifolia (2)*26.2. Ngunduza
*Helichrysum isolepsis (2)*

27. Strandfontein
*Ceropegia occidentalis (2), Prismatocarpus fastigiatus (1), Relhania silicicola (1), Romulea sinispinosensis (1), Sutera multiramosa (2), Tylecodon fragilis (3), Wahlenbergia polyclada (1)*

28. West Outeniekwaberg
*Diosma aristata (1), Euphorbia bayeri (1), Haworthia kingiana (2), Haworthia parksiana (1)*

29. Amathole Mountains
*Aspalathus katbergensis (2), Disa sanguinea (3), Erica brownleeae (9)*29.1. Amathole Mountains Core
*Disa sanguinea (3)*29.2. Western Amathole
*Aspalathus katbergensis (2)*

30. Southern Drakensberg Centre
*Disa galpinii (3), Disa montana (2), Erica cooperi var. missionis (8)*30.1. Southern Drakensberg
*Disa galpinii (3), Disa montana (2)*

31. Great Namaqualand Coastal Centre
*Amellus coilopodius (2), Haemanthus pubescens subsp. arenicolus (5), Merxmuellera rangei (5)*31.1. Sonnikwa Namaqualand
*Amellus coilopodius (2)*31.2. Rosh Pina
*Merxmuellera rangei (5)*

32. Karookop
*Lapeirousia montana (1), Lotononis venosa (1), Pectinaria longipes (2)*

33. Swartruggens
*Acmadenia tetracarpellata (1), Amphiglossa susannae (2)*

34. Southern Great Karoo
*Euphorbia albertensis (1), Tritonia tugwelliae (3)*

35. Western Drakensberg
*Corycium alticola (3), Erica flanaganii (5)*

36. Kokstad
*Corycium tricuspidatum (3), Erica caffrorum var. glomerata (2)*

37. Southeast Great Karoo
*Agathosma bicornuta (2), Euryops gracilipes (1)*

38. Northern Eastern Cape Escarpment

*Pentaschistis microphylla (6)*

39. Garies-Nuwerus
*Ferraria brevifolia (3)*

40. Northern NE Escarpment
*Erica merxmuelleri (2)*

41. Central Tankwa
*Braunsia stayneri (1), Haemanthus tristis (1)*

42. Natal North Coast
*Didymoplexix verrucosa (2), Habenaria woodii (2)*

43. Transvaal Highveld
*Brachycorthis conicosa ssp transvaalensis (4)*

44. Kwaceza
*Schizochilus gerrardii* (2)

45. Western Drakensberg2
*Pentaschistis basutorum* (7)

**46. Hantamsberg***Trogophyton acocksianum (1)*

47. East Grootrivierberg
*Encephalartos lehmannii (1)*

48. East Soutpansberg
*Mystacidium brayboniae (1)*

49. North Barberton
*Holothrix culveri (1)*

50. Adelaide
*Euryops ciliatus (2)*

51. Bhakaneni
*Habenaria ciliosa (1)*

52. NE Wolkeberg
*Polystachya albescens ssp imbricata (1)*

53. McDougall's Bay
*Arctotis Merxmuelleri (1)*

54. Swartkop
*Chaetobromus involucratus ssp sericeus (1)*

55. Landplaas
*Crassula multiceps (1)*

56. Sekameng
*Pentaschistis insulare (1)*

57. Tongaat
*Bonatea saundersiae (1)*

58. Stormsberg
*Euryops calvescens (2)*

59. Kubiskouberge-Langeberg
*Haemanthus dasyphyllus (2)*

60. Brandvlei
*Erica alopecurus var. glabrifolia (1)*

61. Windhoek
*Haemanthus avasmontanus (1)*

62. Koingnaas
*Felicia microsperma (1)*

63. Lebombo
*Restio zuluensis (1)*

64. Joubertsberge
*Aspalathus acicularis planifolia (1)*

65. Mbazwana
*Bonatea lamprophylla (1)*

66. SE Barberton
*Polystachya zuluensis (1)*

67. Not assigned to CoE (widespread or distant from the CFR)
*Elytrophorus globularis (28), Elytrophorus spicatus (6), Haemanthus montanus (26), Pentaschistis borussica (1), Pentaschistis minor (1)*
